# Rapid dynamics allow the low-abundance RTEL1 helicase to promote telomere replication

**DOI:** 10.1093/nar/gkaf177

**Published:** 2025-03-13

**Authors:** Guanhui Wu, Erin Taylor, Daniel T Youmans, Nausica Arnoult, Thomas R Cech

**Affiliations:** Department of Biochemistry, University of Colorado Boulder, Boulder, CO 80303, United States; BioFrontiers Institute, University of Colorado Boulder, Boulder, CO 80303, United States; Howard Hughes Medical Institute, University of Colorado Boulder, Boulder, CO 80303, United States; Department of Molecular, Cellular, and Developmental Biology, University of Colorado Boulder, Boulder, CO 80303, United States; Department of Biochemistry, University of Colorado Boulder, Boulder, CO 80303, United States; BioFrontiers Institute, University of Colorado Boulder, Boulder, CO 80303, United States; Howard Hughes Medical Institute, University of Colorado Boulder, Boulder, CO 80303, United States; Department of Molecular, Cellular, and Developmental Biology, University of Colorado Boulder, Boulder, CO 80303, United States; Department of Biochemistry, University of Colorado Boulder, Boulder, CO 80303, United States; BioFrontiers Institute, University of Colorado Boulder, Boulder, CO 80303, United States; Howard Hughes Medical Institute, University of Colorado Boulder, Boulder, CO 80303, United States

## Abstract

Regulator of telomere length 1 (RTEL1) helicase facilitates telomere replication by disassembling DNA secondary structures, such as G-quadruplexes and telomeric loops (t-loops), at the ends of the chromosomes. The recruitment of RTEL1 to telomeres occurs during the S-phase of the cell cycle, but the dynamics of the process has not been studied. Here, we utilized CRISPR genome editing and single-molecule imaging to monitor RTEL1 movement within human cell nuclei. RTEL1 utilizes rapid three-dimensional diffusion to search for telomeres and other nuclear targets. Only 5% of the chromatin-bound RTEL1 is associated with telomeres at any time in the S-phase, but the telomere-bound RTEL1 has much more extended associations. This binding is enhanced by the interaction between RTEL1 and the telomeric protein TRF2 but is largely independent of RTEL1 ATPase activity. The absence of RTEL1 catalytic activity leads to severe defects in cell proliferation, slow progression out of S-phase, and chromosome end-to-end fusion events. We propose that the rapid diffusion of RTEL1 allows this low-abundance protein to explore the nucleus, bind TRF2, and be recruited to telomeres.

## Graphical Abstract

**Graphical Abstract:**
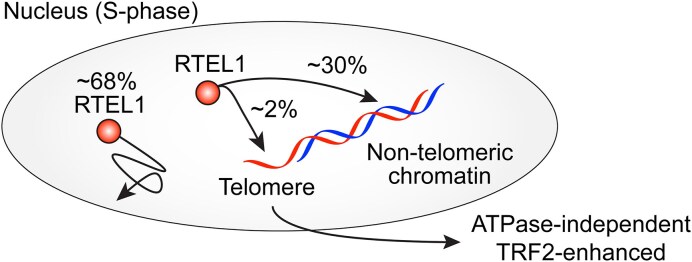

## Introduction

Human telomeres, the ends of chromosomes, play critical roles in cancer and aging [1, 2]. Telomeric DNA comprises guanine (G)-rich tandem repeats of TTAGGG, terminating in a 100–300 nucleotide single-stranded 3′ overhang [3]. The telomere-associated Shelterin protein complex protects chromosome ends from DNA repair activities and nucleolytic degradation [4]. However, due to the end-replication problem, telomeric DNA gradually shortens with each round of cell replication in most somatic cells, eventually leading to cell death or senescence [5]. The Shelterin complex recruits telomerase to extend the single-stranded 3′ DNA overhang [6–8]. Following this, the CST (CTC1–STN1–TEN1) complex loads on the single-stranded DNA and recruits Polα–primase to complete complementary C-strand DNA synthesis [9–12].

Telomeric DNA is particularly difficult to replicate due to its G-rich sequences, which are prone to forming G-quadruplex secondary structures. Single-strand DNA binding proteins, including POT1, RPA, and the CST complex, prevent the folding of these structures [13, 14]. In addition, the Shelterin complex, particularly TRF2, is crucial for releasing the topological stresses in telomeric DNA [15, 16]. TRF2 potentially recruits multiple helicases, such as RTEL1, WRN, and BLM, to help unfold these DNA secondary structures [17–20]. Cells lacking any of these helicases show significant telomere defects [21–23].

RTEL1 is a DEAH-family DNA helicase that plays a critical role in DNA replication, genome stability, DNA repair, and telomere maintenance [22]. Its gene was discovered to be amplified on chromosome 20 in human gastrointestinal tract tumors and later shown to be essential for tumor growth and disease progression [24–27]. When knocked out from mouse cells, *Rtel1* was found to be an essential gene that regulates telomere length and is necessary for DNA repair [28, 29]. Recent research has indicated that a single mutation in *Rtel1* leads to stably short telomeres in mice [30]. In humans, germline homozygous RTEL1 mutations result in severe telomere-related disorders, including Dyskeratosis congenita (DKC) and Hoyeraal-Hreidarsson syndrome (HHS) [31–33], while heterozygous RTEL1 mutations are associated with familial pulmonary fibrosis with no hematologic phenotypes [34, 35]. According to the Orphanet database (https://www.orpha.net/en/disease), DKC and HHS are estimated to affect <1–9 in 1 000 000 individuals, while pulmonary fibrosis affects 1–5 in 10 000, with no cure available for these disorders. A common feature of these diseases is the improper maintenance of telomeres, leading to lung or bone marrow fibrosis and premature death [36].

RTEL1 is a modular protein, with its N-terminus being an ATP-dependent helicase core [22]. Assuming that RTEL1 resembles other DNA helicases, ATP hydrolysis would cause a conformational change of the protein, allowing it to release the nucleic acid product and prepare for the next cycle of catalytic activity [37]. The RTEL1 C-terminal region contains multiple protein binding domains. Specifically, the C4C4 motif directly recognizes the TRFH domain of TRF2 in the S-phase of the cell cycle, which is essential for RTEL1 interactions with telomeres [20]. Mutation of the C4C4 motif or the TRFH domain has been shown to eliminate the RTEL1–TRF2 interaction [20]. However, the dynamic recruitment of RTEL1 to telomeres is still poorly understood. One significant challenge is the simultaneous visualization of RTEL1 and telomeres in living cells, which requires advanced techniques.

We tackled this challenge by combining CRISPR-Cas9 genome editing of the endogenous *RTEL1* and *TRF2* loci with sequences encoding fluorescent tags and single-molecule live-cell imaging. Our results showed that RTEL1 diffuses quickly through the nucleus and can exist in free and bound states. When bound, RTEL1 often localizes at telomeres, especially during the S-phase. Moreover, RTEL1 interacts with telomeres longer than with other nuclear regions. When cells produce only ATPase-dead (K48R) mutant RTEL1, they display various abnormalities, including reduced ability to form colonies, abnormal cell cycle, high morphological heterogeneity, shorter telomeres, and telomere fusions. Surprisingly, the K48R mutation only affects RTEL1’s activity, not its association with telomeres. Our results suggest the previously identified RTEL1-TRF2 interaction is essential for RTEL1 to bind and establish long-lasting interactions with telomeres. Overall, our findings establish a new model for cellular dynamics and telomere recruitment for RTEL1.

## Material and methods

### Cell lines and cell culture

All cell lines were derived from a HeLa-EM2-11ht cell line [38]. The parental HeLa-EM2-11ht cells were cultured in high-glucose Dulbecco's Modified Eagle Medium (DMEM) supplemented with 10% fetal bovine serum (FBS), 1 × GlutaMAX-I, 1 × Penicillin-Streptomycin at 37°C with 5% CO_2_. The doubly genome-edited cell lines were maintained in the same medium. Live cell imaging experiments were conducted in FluoroBrite DMEM supplemented with 10% FBS, 1 × GlutaMAX-I in a humidified imaging chamber heated to 37°C.

### Plasmids and genome editing

All plasmids (Supplementary Figs S1–S3, and Supplementary Donor Vector Sequences) were created using traditional molecular cloning methods and confirmed by whole plasmid sequencing at Plasmidsaurus Inc.

Cell transfections used lipofectamine 3000. For the genome editing, HeLa-EM2-11ht cells were seeded in a 6-well plate on day one. The next morning, the cells were co-transfected with four plasmids: 625 ng *RTEL1* donor vector (including a puromycin-resistance gene), 625 ng *TRF2* donor vector (including a blasticidin-resistance gene), 625 ng plasmid encoding Cas9 and a sgRNA targeting *RTEL1*, and 625 ng plasmid encoding Cas9 and a sgRNA targeting *TRF2* (see Supplementary materials). Guide sequence inserts were incorporated into px330 plasmids as described previously (Supplementary Table S2) [39]. The culture medium was refreshed 6 h after the transfection to allow the cells to recover. The following day, the cells were treated with 1 μg/mL puromycin and 2.5 μg/mL blasticidin for one week and expanded into a T75 flask for another week. After the selection and expansion, cells were labeled with 500 nM JF549 and sorted using a BD FACSAria Fusion Flow Cytometer against JF549 and mEos3.2 fluorophores. Single clones were screened using “In-out” and “Junction” polymerase chain reaction (PCR) approaches (Supplementary Figs S1–S3, and Supplementary Table S2). For homozygous clones and WT clone 15, the “Junction” PCR products were confirmed (Supplementary Figs S1C and D and S3C) by Nanopore sequencing at Plasmidsaurus Inc.

### S-phase cell synchronization and cell cycle analysis

To synchronize the cell cycle using a double thymidine block, cells were first arrested in DMEM containing 2 mM thymidine for 16 h, then released for 9 h, and finally subjected to a second thymidine arrest for 16 h.

For cell cycle analysis using 5-ethynyl-2′-deoxyuridine (EdU), cells were treated with 30 μM EdU for 20 min before harvesting. Downstream analysis utilized the Click-iT EdU Alexa Fluor 488 Flow Cytometry Assay Kit (cat# C10425, ThermoFisher Scientific), following the manufacturer's protocol.

For cell cycle analysis using propidium iodide (PI), cells were harvested at desired time points after the second thymidine release, fixed with 66% ethanol in phosphate-buffered saline (PBS), stained with PI, and analyzed by flow cytometry and FlowJo software.

### Single-molecule live-cell imaging

We followed the previously described imaging method [40] with minor modifications. In brief, cells were seeded in 35 mm imaging dishes (cat# 81 158, Ibidi) and in some cases synchronized to S-phase or G1/S boundary. Cells were then labeled with 0.5 nM JF657 for 15 min and washed with FluoroBrite DMEM three times. A Nikon Ti2-E epifluorescence microscope equipped with an automated TIRF arm and two Andor Ixon 897 EMCCDs coupled via a Cairn TwinCam dual imaging system was used to acquire all images. Acquisition settings and triggering for the lasers and camera were controlled using NIS Elements v5.4.1. Prior to imaging, the cameras were aligned using a beam splitter that included the appropriate Chroma emission filters (525/50 nm, 700/75 nm) for the 488 and 647 nm lasers, respectively. For the kinetic studies, the cells were continuously imaged while being simultaneously illuminated with the 488 nm (6% laser power) and 647 nm (30% laser power) lasers for a duration of 10 s with an effective frame rate of ∼134 frames/s, using a 256 × 128-pixel region of interest. To capture long-lasting static interactions, cells were imaged using 488 nm (1% laser power) and 647 nm (6% laser power) lasers for 90 s at an effective frame rate of five frames/s (100 ms exposure times at 200 ms intervals), using a 256 × 256-pixel region of interest.

### Single-particle tracking and data analysis

Single-particle tracking of RTEL1 used SLIMfast in MATLAB 2024a to analyze TIFF files [41]. The settings used were as follows: Localization error = –5, Emission wavelength = 672 nm, Exposure time = 7.5 ms, Deflation loops = 0, D_max_ = 5 μm^2^/s, Number of gaps allowed = 2, NA = 1.49, Pixel size = 0.16 μm. As described previously [40], the RTEL1 tracks were filtered against TRF2 to classify them into telomere or non-telomere groups. The tracks were then used in SpotOn to determine diffusion coefficients and the percentage of bound versus free particles. The following settings were used in SpotOn: TimeGap = 7.5 ms, dZ = 0.700 μm, GapsAllowed = 2, TimePoints = 9, BinWidth = 0.01 μm, JumpsToConsider = 4, PDF-fitting, D_Free_2State = [0.5 10], D_Bound_2State = [0.0001 0.5]. The single-molecule live-cell imaging experiments were conducted at least three times on different days with >20 cells per experiment.

For the analysis of low-mobility particles, single-particle tracking was conducted at the following settings to exclusively track low-mobility molecules: Localization error = –5, Emission wavelength = 672 nm, Exposure time = 200 ms, Deflation loops = 0, D_max_ = 0.5 μm^2^/s, Number of gaps allowed = 2, NA = 1.49, and Pixel size = 0.16 μm. Track length distributions were plotted as probabilities (1 – Cumulative density function of the track lengths).

### Immunoprecipitation and Western blot

For immunoprecipitation (IP), cells from six 150 mm dishes were harvested and resuspended in CHAPS lysis buffer (10 mM Tris-HCl pH 7.5, 1 mM MgCl_2_, 1 mM EGTA, 0.5% CHAPS, 1.0 mM 2-mercaptoethanol, 10% glycerol, 1 × Pierce protease inhibitor tablets (cat# A32965, ThermoFisher Scientific). RTEL1 IP was performed using a rabbit polyclonal antibody (1:50 dilution, cat# NBP2-22360, Novus Biologicals). FLAG IP was carried out with ANTI-FLAG M2 Affinity Gel (cat# A2220, Sigma-Aldrich).

The protein samples were denatured in 1 × NuPAGE LDS Sample Buffer supplemented with 5% 2-mercaptoethanol at 80°C for 5 min. Proteins were separated on 3–8% Tris-acetate gels. The protein transfer was performed using nitrocellulose membranes with an iBlot 2 Dry Transfer Device. The membrane was probed with the following primary antibodies: polyclonal antibody anti-RTEL1 (1:100 dilution, cat# HPA078328, Sigma-Aldrich), polyclonal antibody anti-TRF2 (1:500 dilution, cat# NB110-57130, Novus Biologicals), monoclonal antibody anti-HA (1:250 dilution, cat# 2367, Cell Signaling), monoclonal antibody anti-β-Actin (1:4000, cat# A5441, Sigma-Aldrich). SuperSignal West Femto Maximum Sensitivity Substrate (ThermoFisher Scientific) was used in the HRP reactions to visualize the bands.

### Telomere length analysis

Telomeric length analysis was carried out using established methods with minor modifications [6]. Genomic DNA was extracted from five million cells using the GenElute Mammalian Genomic DNA Miniprep Kit (Sigma-Aldrich). Then, 1.5 μg of genomic DNA was digested with Hinf1 and RsaI overnight at 37°C and separated on a 0.8% agarose gel in 1 × TBE at 50 V for 22 h. A 5′-end ^32^P-labeled λ DNA-HindIII digest ladder (10 000 c.p.m.) was used as a marker. After the transfer, the membrane was irradiated with UV (260 nm; set at 1 200 μJ × 100 energy) and pre-hybridize in 15 mL PerfectHyb Plus Hybridization Buffer (Sigma-Aldrich) for 30 min at 50°C in a hybridization oven with rotation. Following the pre-hybridization, the buffer was replaced with 15 mL PerfectHyb Plus buffer containing 5′-end ^32^P-labeled (TTAGGG)_4_ probe (100 000 000 c.p.m) and incubated for another 5 h at 50°C in a hybridization oven with rotation. After the incubation, the membrane was washed three times with buffer containing 0.1 × SSC and 0.1% sodium dodecyl sulfate (SDS) and exposed to a phosphorimager screen overnight.

### Cellular abundance of RTEL1 by FACS

0.3 million genome-edited HeLa cells were seeded in six-well plates. Two days after plating, cells were labeled with 500 nM JF646 for 30 min in a tissue culture incubator. Then, the cells were washed three times with PBS, trypsinized, centrifuged, and resuspended in 1 × PBS + 1% FBS before passing through cell strainers. Approximately 20 000 single-cell events were collected using a BD Accuri C6 Plus Flow cytometer for flow cytometry. The results were analyzed using FlowJo software, and the single cells were gated using forward and side scattering. The cells expressing Halo-CTCF were kindly provided by the Darzacq lab (UC Berkeley), and the absolute copy number of Halo-CTCF had been determined previously [42].

The absolute copy number (n) of Halo-RTEL1 per cell was calculated using mean fluorescence intensity (MFI) according to:


\begin{eqnarray*}&&{{{\mathrm{n}}}_{{\mathrm{Halo}} - {\mathrm{RTEL}}1}} = \frac{{{\mathrm{\ }}\left( {{\mathrm{MF}}{{{\mathrm{I}}}_{{\mathrm{Halo}} - {\mathrm{RTEL}}1;{\mathrm{\ }} + {\mathrm{JF}}646}} - {\mathrm{MF}}{{{\mathrm{I}}}_{{\mathrm{Halo}} - {\mathrm{RTEL}}1; - {\mathrm{JF}}646}}} \right) - {\mathrm{\ }}({\mathrm{MF}}{{{\mathrm{I}}}_{{\mathrm{Background}};{\mathrm{\ }} + {\mathrm{JF}}646}} - {\mathrm{MF}}{{{\mathrm{I}}}_{{\mathrm{Background}}; - {\mathrm{JF}}646}})}}{{{\mathrm{\ }}\left( {{\mathrm{MF}}{{{\mathrm{I}}}_{{\mathrm{Halo}} - {\mathrm{CTCF}};{\mathrm{\ }} + {\mathrm{JF}}646}} - {\mathrm{MF}}{{{\mathrm{I}}}_{{\mathrm{Halo}} - {\mathrm{CTCF}}; - {\mathrm{JF}}646}}} \right) - {\mathrm{\ }}({\mathrm{MF}}{{{\mathrm{I}}}_{{\mathrm{Background}};{\mathrm{\ }} + {\mathrm{JF}}646}} - {\mathrm{MF}}{{{\mathrm{I}}}_{{\mathrm{Background}}; - {\mathrm{JF}}646}})}} \times {{{\mathrm{n}}}_{{\mathrm{Halo}} - {\mathrm{CTCF}}}}\end{eqnarray*}

MFI _+JF646_ represents the MFI with JF646-labeling. MFI _-JF646_ represents the MFI without JF646-labeling. MFI _background; +JF646_ represents the background for JF646 labeling, which was determined using parental HeLa cells.

To account for the portion of RTEL1 that had lost its HaloTag, the fluorescence values were corrected by the relative band intensities shown by the western blot to estimate the absolute copy number of total RTEL1 per cell according to:


\begin{eqnarray*}{{{\mathrm{n}}}_{{\mathrm{total\ RTEL}}1}} = \frac{{{\mathrm{Band\ Intensit}}{{{\mathrm{y}}}_{{\mathrm{RTEL}}1}} + {\mathrm{Band\ Intensit}}{{{\mathrm{y}}}_{{\mathrm{Halo}} - {\mathrm{RTEL}}1}}}}{{{\mathrm{Band\ Intensit}}{{{\mathrm{y}}}_{{\mathrm{Halo}} - {\mathrm{RTEL}}1}}}} \times {{{\mathrm{n}}}_{{\mathrm{Halo}} - {\mathrm{RTEL}}1}}\end{eqnarray*}

The band intensities were determined using ImageJ. No correction was needed for the ATPase-dead RTEL1, which showed only the Halo-tagged species.

### Metaphase FISH

Cells were synchronized in metaphase for 3 h with 0.1 mg/mL colcemid (cat# 15212–012, Gibco KaryoMAX). Media and cells were collected, pooled, centrifuged, and subjected to hypotonic shock in 10 mL 75 mM KCl for 8 min at 37°C, then fixed and washed three times in methanol : glacial acetic acid 3:1 (v/v). Metaphases were then dropped onto superfrost microscope slides and dried overnight. For FISH, slides were rehydrated for 10 min in PBS, fixed with 3.7% formaldehyde for 10 min, washed for 5 min in PBS, then treated for 10 min at 37°C with 1 mg/mL pepsin in citric acid pH 2, washed three times in PBS and fixed again in formaldehyde for 10 min. After three PBS washes, the slides were dehydrated in consecutive ethanol baths (75%, 95%, and 100%; 3 min each) and allowed to air dry. Slides were then layered with 40 μL of 0.3 ng/mL Alexa 488-OO- (CCCTAA)_3_ PNA probe (cat# F1001, PNA Bio Inc.) and 0.3 ng/mL CENP-B Cy3 (ATTCGTTGGAAACGGGA) PNA probe (cat# F3002, PNA Bio Inc.) diluted in 70% (v/v) deionized formamide; 0.25% (v/v) blocking reagent (NEN); 10 mM Tris pH 7.5; 4.1 mM Na_2_HPO_4_; 450 mM citric acid; 1.25 mM MgCl_2_, denatured for 5 min at 84°C and incubated for 5 h at room temperature. Slides were then washed twice for 15 min in 70% (v/v) formamide; 10 mM Tris-HCl pH 7.5 and three times for 5 min in 50 mM Tris-HCl pH 7.5; 150 mM NaCl; 0.08% Tween-20. Slides were mounted with Vectashield Plus DAPI (Vector Laboratories H-1900). Metaphases were then imaged using a Nikon Widefield epi-fluorescence inverted microscope and a 100 × objective. Fusions and signal-free ends were counted manually using Image J software. Image acquisition and analysis were conducted while blinded to the experimental conditions to ensure unbiased quantification.

## Results

### Generation and characterization of doubly genome-edited cell lines

The *RTEL1* gene comprises 35 exons, encoding two main protein isoforms of 1219 and 1300 amino acids [43]. It has been shown that the full-length isoform is the primary one involved in telomere length regulation [44]. To specifically visualize this RTEL1 isoform in live cells, we introduced sequences encoding a FLAG-HaloTag along with the cDNA for the isoform at the endogenous *RTEL1* loci in HeLa cells (Supplementary Fig. S1), using a CRISPR-Cas9 genome editing strategy adapted from our prior work [45]. In preliminary studies, we found that both N-terminal and C-terminal Halo-tagged proteins were expressed at the correct molecular weight following transient transfection (Supplementary Fig. S1E). We chose a C-terminal tag because it was more practical for the subsequent CRISPR knock-in of mutations in RTEL1.

**Figure 1.**
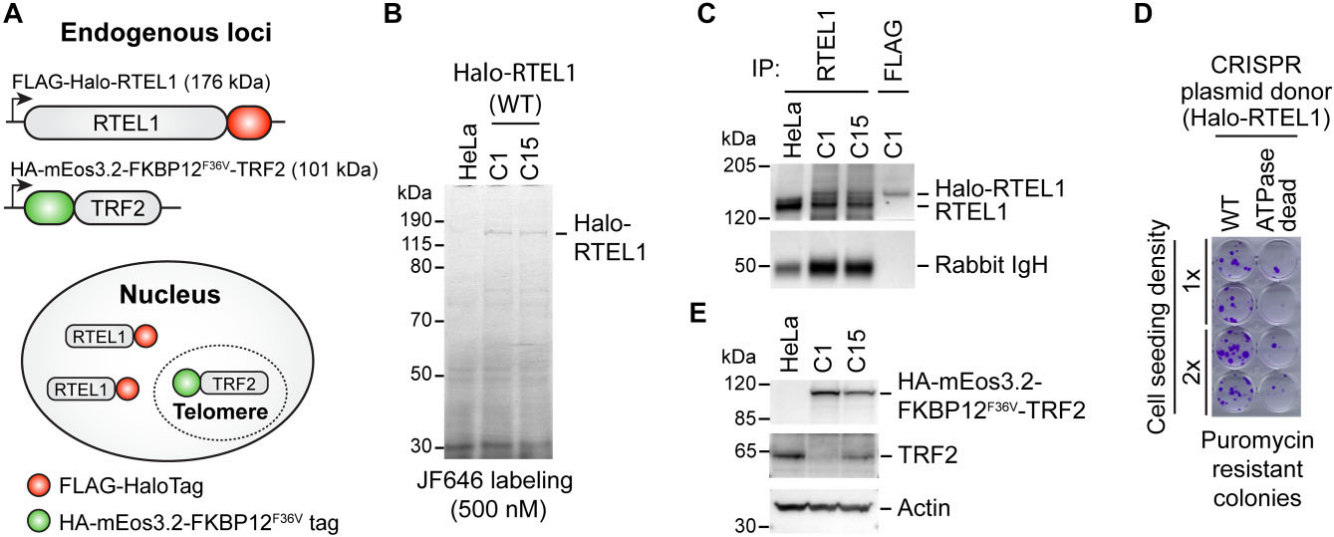
Generation and characterization of cell lines expressing WT FLAG-HaloTag-RTEL1 from its endogenous loci. (**A**) Schematic representation of the doubly genome-edited cell lines used to track single RTEL1 molecules and to visualize telomeres. RTEL1 and TRF2 were fused with FLAG-HaloTag and HA-mEos3.2-FKBP12^F36V^ tag, respectively. (**B**) SDS-PAGE of total cellular proteins from two doubly genome-edited HeLa cell clones (C1 and C15) labeled with JF646 and imaged for fluorescence. (**C**) Western blot of RTEL1 using RTEL1-IP products from parental HeLa and two doubly genome-edited clones. RTEL1-IP and western blot utilized antibodies recognizing different RTEL1 epitopes. Another biological replicate is shown in Supplementary Fig. S12. C1 and C15 are fully genome-edited (Supplementary Figs S1–S3), so bottom RTEL1 band presumably had its FLAG-HaloTag removed by proteolysis in the cell or during purification. (**D**) Colony formation analysis for HeLa cells complemented with a donor plasmid encoding WT or ATPase-dead (K48R) FLAG-HaloTag RTEL1 with a puromycin-resistance gene. Crystal violet-stained plates were photographed after 13 days of puromycin selection. (**E**) Western blot of HA-mEos3.2-FKBP12^F36V^ tagged TRF2 and endogenous TRF2 from parental HeLa and two doubly genome-edited clones, probed with anti-HA and anti-TRF2 antibodies, respectively. C1 is fully genome-edited, while C15 has both edited and unedited TRF2 loci (Supplementary Fig. S3).

The desired genome-edited cell clones were identified through PCR screening (Supplementary Fig. S2). The correct insertion of the FLAG-HaloTag and the absence of any unexpected mutations in RTEL1 were further confirmed through isolation of genomic DNA, PCR amplification of the locus, and Nanopore DNA sequencing following clonal expansion (Supplementary Figs S1B-D). The HaloTag [46], designed to bind fluorescent ligands covalently, allowed us to visualize the Halo-tagged proteins in cells when treated with the fluorescent ligand JF646 (Fig. 1A). SDS-polyacrylamide gel electrophoresis (PAGE) analysis indicated that the fluorescent-labeled FLAG-HaloTag RTEL1 ran at the correct molecular weight (Fig. 1B), showing the specific labeling of the FLAG-HaloTag RTEL1. However, we could not directly detect endogenous or FLAG-HaloTag RTEL1 using Western blot from crude cell extracts.

Subsequently, we enriched total RTEL1 proteins by IP using an RTEL1 antibody and detected RTEL1 in Western blot using a different RTEL1 antibody. The results revealed that genome-edited cell lines express similar amounts of total RTEL1 to their parental HeLa cells (Fig. 1C).

ATP hydrolysis activity is critical for RTEL1’s function [47]. Despite RTEL1’s low abundance, the introduction of ATPase-dead (K48R) Halo-tagged RTEL1 greatly compromised the clonogenic capability of HeLa cells (Fig. 1D), indicating its essential role in these human cells. This result also confirmed the cellular activity of the wild-type (WT) Halo-RTEL1 fusion protein, because it was sufficient to support normal cell growth in edited cell lines.

TRF2 binds double-stranded telomeric DNA and is part of the Shelterin complex. Our previous studies found that fusing the N-terminus of TRF2 with the mEos3.2 fluorescent protein does not affect TRF2’s functions, and the fusion protein can serve as a marker for telomeres [40]. Therefore, we labeled TRF2 with an N-terminal HA-mEos3.2-FKBP12^F36V^ tag using the CRISPR-Cas9 approach to visualize telomeres. We confirmed the proper insertion of the HA-mEos3.2-FKBP12^F36V^ tag by PCR and Nanopore sequencing (Supplementary Fig. S3). Western blot analysis verified the correct expression of TRF2 and fusion proteins (Fig. 1E).

Furthermore, we characterized the telomere lengths in nine double knock-in clones (Supplementary Fig. S4). These clones had correct genomic insertion and expressed the appropriate fusion proteins (Supplementary Figs S4A–D). We observed substantial variation in telomere lengths among these clones (Supplementary Fig. S4E), with no correlation between telomere length and the expression level of the fusion protein (Figs. 1B, C, and E and Supplementary Figs S4C and D). Because multiple clones exhibited similar or even longer telomeres than the parental HeLa cells, we conclude that the expression of RTEL1 fusion proteins was not the underlying cause of the clonal heterogeneity. Instead, the substantial variation in telomere length between different clones may have preceded the genome editing and became apparent upon single-cell isolation using FACS. Indeed, previous studies have shown clonal heterogeneity in telomere length among cancer cell lines, including HeLa [48, 49]. To further validate the functions of Halo-tagged RTEL1 protein, we carried out metaphase spreads using WT C1 and parental HeLa cells. Their similarly low levels of telomere fragility support the normal functions of fused proteins (Supplementary Fig. S5).

### Low abundance of RTEL1 in HeLa cells

To estimate the cellular abundance of RTEL1 protein, we compared the relative fluorescence intensities between cells expressing Halo-tagged RTEL1 and Halo-tagged CTCF, both labeled with JF646, using flow cytometry (Supplementary Fig. S6A). A previous study determined that U2OS cells have ∼0.1 million Halo-CTCF per cell [42], making these cells a convenient standard for measuring the cellular abundance of other proteins. Comparison of the fluorescence intensities of Halo-tagged RTEL1 and CTCF cells by flow cytometry indicated that each HeLa cell expresses only ∼3000 copies of RTEL1 protein (Supplementary Fig. S6B). This low protein abundance is consistent with our finding that we needed to first enrich RTEL1 before it could be detected by Western blot.

### RTEL1 displays freely diffusing and chromatin-bound states

**Figure 2.**
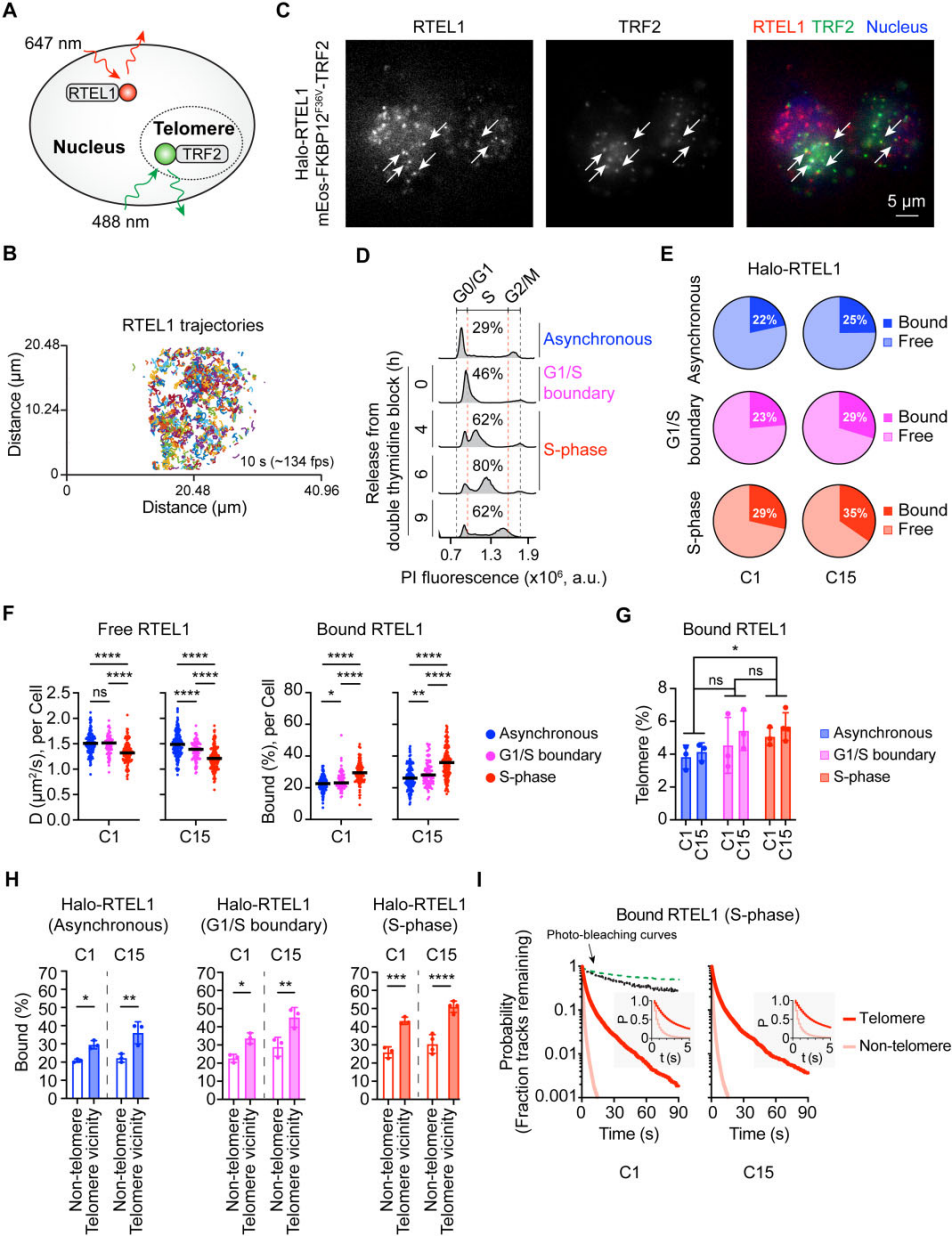
Live cell imaging analysis of WT RTEL1 and its interactions with telomeres. (**A**) Schematic representation of the method used to visualize RTEL1 and telomeres. JF657 labeled HaloTag-RTEL1 and mEos3.2-TRF2 were excited using 647 and 488 nm lasers, respectively, and simultaneously imaged at ∼134 frames per second (fps). (**B**) All RTEL1 trajectories in a 10-second movie were generated by single particle tracking of RTEL1 at ∼134 fps. (**C**) A representative still image showing simultaneous visualization of RTEL1 (HaloTag-JF657) and telomeres (mEos3.2-TRF2). The cell nucleus was visualized using Hoechst dye. White arrows indicate colocalizations of RTEL1 and telomeres. (**D**) Flow cytometry analysis of propidium iodide (PI) stained doubly genome-edited cell lines after release from double thymidine block. (**E**) Pie charts summarize the overall percentages of RTEL1 in bound and free states from asynchronous, G1/S boundary, and S-phase cells, as determined using Spot-on software. Live cell imaging data were collected 4–6 hours (h) after the release for S-phase cells. *n*> 50 cells per condition across 3–4 biological replicates. (**F**) Left: The diffusion coefficients of free RTEL1 from single cells at asynchronous, G1/S boundary, and S-phase states. Right: The percentages of bound RTEL1 from cells at these states. Each data point represents a single cell. The solid line represents the overall median. *n*> 50 cells per condition across 3–4 biological replicates. *P < 0.0332, **P < 0.0021, ****P < 0.0001 was calculated by Fisher's LSD test after one-way ANOVA. (**G**) Comparison of percentages of bound RTEL1 at telomeres between asynchronous, G1/S boundary, and S-phase cells. Bound RTEL1 tracks were assigned to telomeres when they were within 3 pixels (0.48 μm) of the centroid of a TRF2 signal. Error bars represent mean ± SD for 3–4 biological replicates. *n*> 20 cells per replicate. *p< 0.0332 was calculated by Fisher's LSD test after two-way ANOVA. (**H**) Comparison of bound percentages for RTEL1 molecules close to telomeres (telomere vicinity) and those not (non-telomere). Error bars represent mean ± SD for 3–4 biological replicates. *n*> 20 cells per replicate. *P < 0.0332, **P < 0.0021, ***P < 0.0002, ****P < 0.0001 were calculated by Fisher's LSD test after two-way ANOVA. (**I**) The comparison of the residence lifetimes of bound RTEL1 between telomeric and non-telomeric regions as a function of time in S-phase cells. *n*> 50 cells per condition across three biological replicates. The photo-bleaching curves show the overall integrated intensity of the RTEL1 channel (lower dashed line) and the TRF2 channel (upper dashed line) changing over time.

After characterizing the cells expressing FLAG-HaloTag RTEL1 and HA-mEos3.2-FKBP12^F36V^-TRF2, we used clones 1 and 15 for live-cell imaging studies, as they represented two different average telomere lengths (4.5 and 2.4 kb, respectively) (Supplementary Fig. S4E). The high-incline laminated optical sheet (HILO) [50] illumination facilitated high-contrast detection of RTEL1 (Fig. 2A). The TRF2 foci diffused extremely slowly (Supplementary Fig. S7A and Supplementary Movie 1), as expected for a protein bound to telomeres and as measured previously [40]. The majority (75–78%) of RTEL1, on the other hand, diffused quickly through the cell nucleus (Fig. 2B, Supplementary Fig. S7B and Supplementary Movie 1). Each fluorescent particle may represent two RTEL1 molecules, given the evidence that human RTEL1 is a dimer [51]. The jump-length distribution of RTEL1 particles fits well to a two-state model, showing fast and slow diffusion states. This indicates that RTEL1 is similar to many other nuclear proteins, including transcription factors, which are present in freely diffusing and bound states [41]. RTEL1 was often found to colocalize with telomeres (Fig. 2C and Supplementary Movie 1), as described below.

### Increased binding of RTEL1 to telomeres and non-telomeric chromatin in S-phase

RTEL1 is thought to act during telomere replication, which in human cells occurs throughout the S-phase of the cell cycle [52, 53]. Its recruitment to the telomere increases at the G1 to S transition and peaks in the S phase [20]. To determine the cellular binding states of RTEL1 during S-phase, cells were synchronized to the G1/S boundary using a double thymidine block and then released into S-phase (Fig. 2D). Movies were recorded at 4–6 h after the release, when S-phase cells peaked. Analysis of single-molecule tracking data using Spot-On software demonstrated that 29–35% of RTEL1 molecules were bound to chromatin during S-phase, while the remaining RTEL1 molecules were rapidly diffusing and presumably searching for their targets (Fig. 2E and Supplementary Movie 2). Asynchronous cells and cells at the G1/S boundary had a lower bound fraction than those in the S-phase (Fig. 2E and Supplementary Movies 1–3). At the single-cell level, the bound fractions and the diffusion coefficients of RTEL1 molecules varied across the cell population (Fig. 2F), demonstrating cellular heterogeneity. Importantly, the free RTEL1 molecules were less mobile during S-phase, and most cells had a higher bound fraction during S-phase, consistent with different functions of RTEL1 in S-phase than in other phases. These differences are quite substantial, considering that about 20–38% of the cells observed during S-phase data collection are outside the S-phase (Fig. 2D), which therefore limits the ability to see differences between S-phase and other cell cycle phases.

Given that telomeres are one of the cellular targets of RTEL1, we next investigated RTEL1’s interactions with telomeres. We utilized a previously established method to categorize RTEL1 tracks into two groups: those in close proximity to telomeres and those that are not [40]. As these two groups of tracks include both static and dynamic RTEL1 molecules, we analyzed them using Spot-On to determine the bound percentage within each group. Finally, we calculated the percentage of telomere-bound RTEL1 within the total bound RTEL1. Approximately 5% of bound RTEL1 localized at the telomeres during the S-phase, which was higher than in asynchronous cells, and this increased binding had already occurred at the G1/S boundary (Fig. 2G). Furthermore, RTEL1 molecules close to telomeres displayed different binding properties compared to those not close to telomeres (Fig. 2H). Telomeric RTEL1 had a higher bound fraction than non-telomeric RTEL1, and this binding fraction was also higher than that of asynchronous cells.

To directly visualize RTEL1 binding to telomeres as a function of time, we plotted kymographs of RTEL1 associating with TRF2 foci (Supplementary Figs S8A and B). RTEL1 formed two types of interactions with telomeres: short-dynamic and long-static interactions. To record these long-static interactions, we reduced the laser power, increased the exposure time, and decreased the image interval to 5 frames per second (fps). In the time-lapse movies, RTEL1 formed much longer interactions with telomeres (t_1/2_ = 2.2–2.4 s) than non-telomeric regions (t_1/2_ = 0.6–0.8 s) (Fig. 2I, Supplementary Figs S8C and D and Supplementary Movie 4). These observations demonstrate that RTEL1 can specifically bind to telomeres in S-phase, where it resides much longer than when it binds to other genomic regions.

### Inhibition of RTEL1 ATPase activity causes cell cycle defects and telomere defects

**Figure 3.**
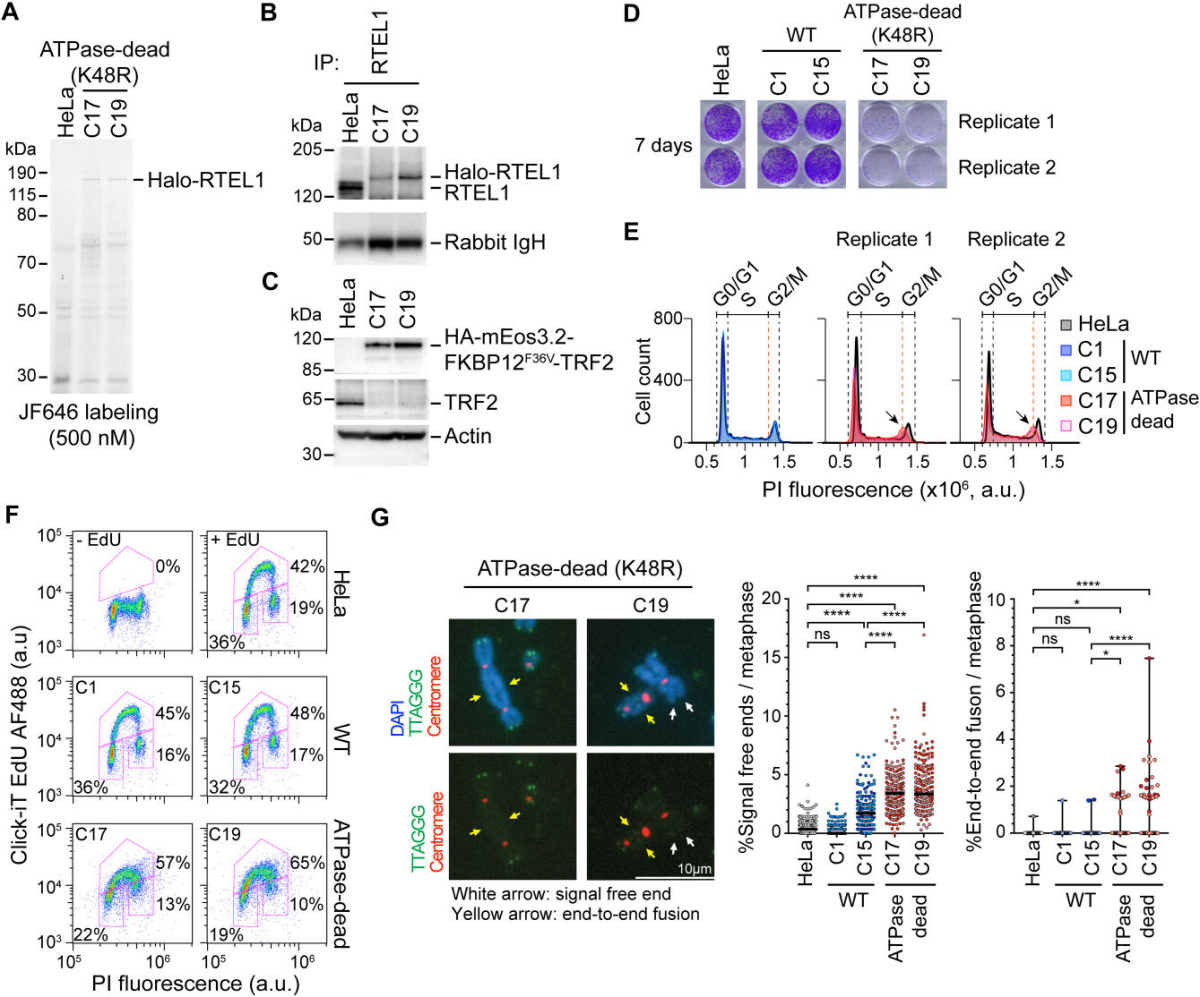
Cell lines expressing ATPase-dead (K48R) RTEL1 have severe proliferation defects, accumulate S-phase cells, and exhibit telomere loss and chromosome fusions. (**A**) SDS-PAGE of fluorescently labeled ATPase-dead (K48R) Halo-RTEL1 from two doubly genome-edited clones (C17 and C19). (**B**) Western blot of ATPase-dead RTEL1 using RTEL1-IP products from parental HeLa and two doubly genome-edited clones. (**C**) Western blot of HA-mEos3.2-FKBP12^F36V^ tagged TRF2 from parental HeLa and two doubly genome-edited clones, probed with anti-HA and anti-TRF2 antibodies. (**D**) Colony formation of HeLa, two WT clones, and two ATPase-dead clones at day 7 after plating. (**E**) Cell cycle comparison of parental HeLa, two WT clones, and two ATPase-dead clones. Left, overlay of HeLa, C1, and C15. Middle and Right, overlay of HeLa, C17, and C19. *n* = 9000–11 000 cells per condition. (**F**) Flow cytometry analysis of HeLa, two WT clones, and two ATPase-dead clones from PI and EdU-AF488 double labeling. HeLa cells that were not subjected to EdU pulse labeling served as a negative control. The duration of EdU pulse labeling was 20 min. *n* = 9000–11 000 cells per condition. (**G**) Representative images (left) and quantifications of signal-free ends (middle) and end-to-end fusions (right) for metaphase spread analysis. Bottom images, DAPI not shown to confirm lack of green signal at chromosome fusion sites and free ends. *n* = 193–201 metaphases per condition across two replicates, N1 = darker coloured and N2 = lighter coloured data points. Line represents median, and bar indicates top quartile range. ns, non-significant, *P < 0.0332, ****P < 0.0001 were calculated by Sidak test after one-way ANOVA.

RTEL1 is an ATP-dependent helicase [47]. We introduced an ATPase-dead mutation (K48R) into the endogenous RTEL1 loci to determine how the loss of ATPase activity affects cellular RTEL1 and cancer cell growth. After puromycin selection and screening over 600 cells, we successfully generated two homozygous clones. PCR and nanopore sequencing confirmed the correct insertion of the K48R mutation in all edited alleles (Supplementary Fig. S1). SDS-PAGE and Western blot analysis showed the proper expression of FLAG-HaloTag K48R mutant RTEL1 (Figs. 3A and B). K48R mutant clones exhibited total RTEL1 levels similar to the levels in WT clones (Supplementary Fig. S6B). To visualize telomeres, TRF2 was marked with the same HA-mEos3.2-FKBP12^F36V^ tag used above (Fig. 3C and Supplementary Fig. S3).

The two RTEL1 K48R homozygous clones showed lower colony formation ability than either parental HeLa cells or cells expressing WT HaloTag-RTEL1 (Fig. 3D), which agreed with the poor colony formation ability during screening. Flow cytometric analyses demonstrated that the two mutant RTEL1 clones displayed higher morphological heterogeneity, with increased cell size and higher granularity (Supplementary Fig. S9), a phenotype associated with senescent cells. Additionally, we observed abnormal cell arrest at late S-phase (Figs. 3E and F). Further analyses revealed that both homozygous clones had significantly shorter telomeres (Supplementary Fig. S10), but it was unclear whether the short telomeres preceded the genome editing or were a consequence of RTEL1 ATPase inactivation.

To further investigate the cellular consequences of RTEL1 ATPase inactivation, we analyzed metaphase spreads. We found striking telomere defects in the mutant RTEL1 clones compared to WT clone 15, including chromosome end-to-end fusions resulting in dicentric chromosomes (Fig. 3G and Supplementary Fig. S11A), as well as sister chromatid fusions (Supplementary Fig. S11B). Additionally, we observed a significant increase in chromosome ends without detectable telomeric DNA hybridization (“signal-free ends”), indicative of critically short or lost telomeres (Fig. 3G and Supplementary Fig. S11A). Notably, these telomeric defects cannot be solely attributed to the short average length of telomeres in these mutant clones, as they were all significantly more pronounced in the mutant clones than in WT HaloTag-RTEL1 clone 15, which has comparably short bulk telomere lengths (Supplementary Figs S4E and S10C). These findings highlight the critical role of RTEL1 ATPase activity in telomere maintenance.

These observations collectively establish the cellular phenotypes associated with ATPase-defective RTEL1 in HeLa cells. The occurrence of signal-free ends is consistent with previous reports of RTEL1 knockout or different RTEL1 mutations in mouse embryonic stem (ES) cells [28], mouse embryonic fibroblasts (MEFs) [54], human HEK293 cells [51], and HHS patient cells [33, 43, 55]. Fragile telomeres have been documented in RTEL1-minus MEFs [29, 54], we however could not quantify fragile telomeres in our mutant RTEL1 cells as telomeres are too short. The end-to-end chromosome fusions seen here upon inactivation of RTEL1 ATPase activity have been previously reported for *Rtel1*^−/−^ mouse ES cells [28] but not to our knowledge for human cells; they are lower in frequency than the end-to-end fusions seen upon knockout of Shelterin components such as TRF2 [56].

### Catalytically inactive RTEL1 retains telomeric localization

**Figure 4.**
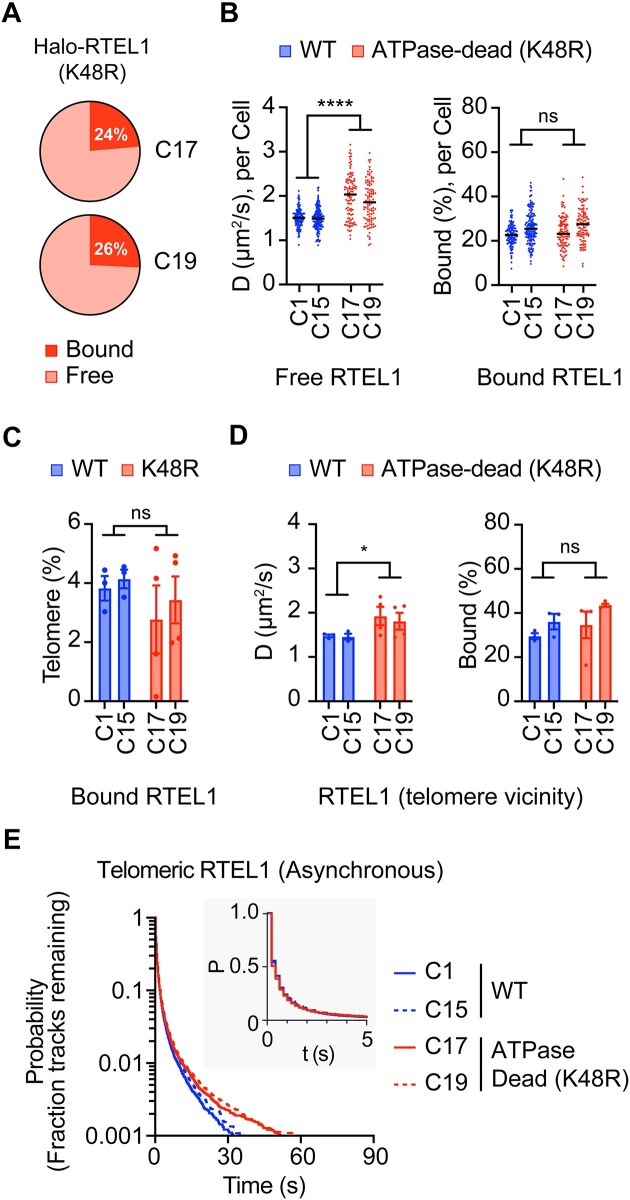
Live cell imaging analysis of ATPase-dead (K48R) RTEL1 and its interactions with telomeres in asynchronous cells. (**A**) Percentages of RTEL1 in bound and free states from two ATPase-dead clones. *n* > 50 cells per condition across four biological replicates. (**B**) Left: Diffusion coefficients of free RTEL1 for WT and ATPase-dead clones. Right: Percentages of bound RTEL1 for WT and ATPase-dead clones. The solid line represents the overall median. Each data point represents a single cell. *n*> 50 cells per condition across 3–4 biological replicates. ****P < 0.0001 was calculated by two-way ANOVA. (**C**) Percentages of bound RTEL1 at telomeres for WT and ATPase-dead clones. Error bars represent mean ± SD for 3–4 biological replicates. *n*> 20 cells per replicate. (**D**) Left: Diffusion coefficients of free telomeric RTEL1 for WT and ATPase-dead clones. Right: Percentages of bound telomeric RTEL1 between WT and ATPase-dead clones. Error bars represent mean ± SD for 3–4 biological replicates. *n*> 20 cells per replicate. *P < 0.0332 was calculated by two-way ANOVA. (**E**) Residence lifetimes for WT and ATPase-dead RTEL1 on telomeres as a function of time in asynchronous cells. *n* > 25 cells per condition, WT across two biological replicates, ATPase-dead across four biological replicates.

We conducted single-molecule live-cell imaging to examine the effects of the K48R mutation on the binding and dynamics of RTEL1. ATP hydrolysis is essential for loading or releasing a helicase from its nucleic acid substrates. Surprisingly, our analysis of K48R mutant RTEL1 revealed dynamics very similar to those of WT RTEL1: 24–26% of ATPase-dead RTEL1 molecules were bound, while the rest moved quickly through the nucleus (Fig. 4A and Supplementary Movie 5). Interestingly, the free ATPase-dead RTEL1 molecules were more mobile while heterogeneous at the single-cell level (Fig. 4B). The investigation of telomeric RTEL1 showed that K48R mutation did not impact the recruitment of RTEL1 to telomeres, although there was an increase in the diffusion coefficient of telomeric RTEL1 (Figs. 4B–D and Supplementary Movie 5). Furthermore, the K48R mutation did not alter the overall half-life of RTEL1 on telomeres, affecting the retention time of less than 1% of RTEL1 on telomeres (Fig. 4E and Supplementary Movies 6–7). These observations suggest that the recruitment and retention of RTEL1 on telomeres are not largely reliant on its ATPase activity.

### TRF2 degradation compromises chromatin-bound RTEL1

**Figure 5.**
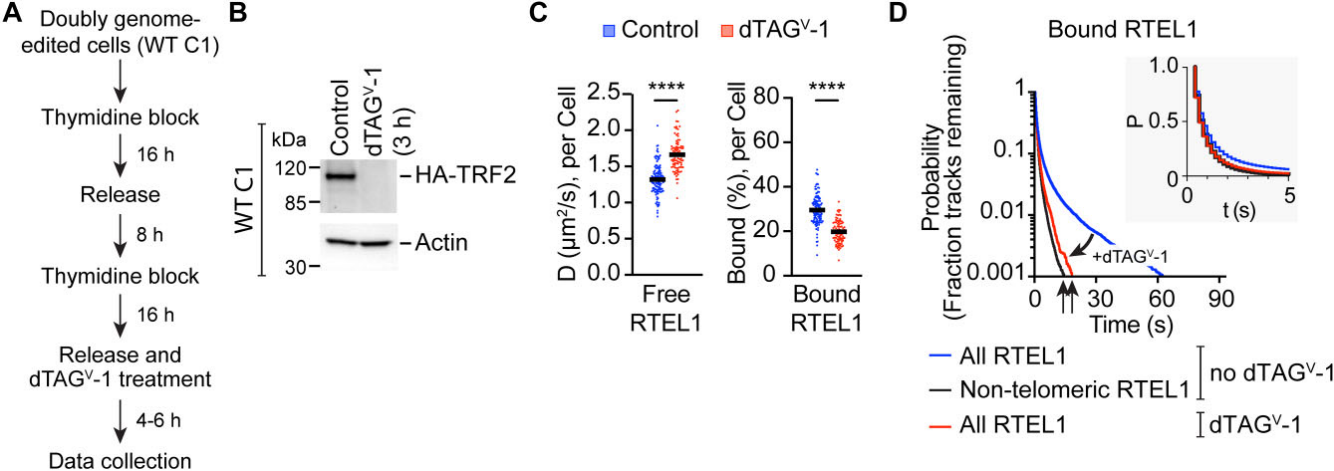
TRF2 mediates the association of RTEL1 and telomeres. (**A**) Schematic representation for the methods used in (C and D). (**B**) Western blot showing the degradation of HA-FKBP12^F36V^-tagged TRF2 following 3 h of dTAG^V^-1 treatment. (**C**) Left: Comparison of the diffusion coefficients of free RTEL1 from single cells with and without dTAG^V^-1 treatment. Right: Comparison of the percentages of bound RTEL1 from single cells with and without dTAG^V^-1 treatment. The solid line represents the overall median. Each data point represents a single cell. *n*> 50 cells per condition across three biological replicates. ****P < 0.0001 was calculated by unpaired t-test. (**D**) Comparison of the residence lifetimes of bound RTEL1 with and without dTAG^V^-1 treatment. The residence lifetime of non-telomeric RTEL1 without dTAG^V^-1 treatment serves as a reference and is shown in the black line. *n* > 25 cells per condition across 2–3 biological replicates.

The telomeric Shelterin protein TRF2 potentially recruits RTEL1 [20]. To better understand how RTEL1 is recruited to telomeres, we synchronized cells to S-phase and degraded TRF2 using dTAG^V^-1, a highly specific degrader targeting FKBP12^F36V^ fusion proteins [57, 58]. Following the treatment, TRF2 was depleted within 3 h (Figs. 5A and B), and fewer stationary RTEL1 molecules were observed (Supplementary Movie 8). When we analyzed the movement of RTEL1, we found a decreased percentage of bound molecules (Fig. 5C). The free RTEL1 molecules showed increased mobility at the individual cell level (Fig. 5C). Furthermore, time-lapse movies demonstrated that after TRF2 degradation, the residence time of bound RTEL1 was significantly reduced, resembling that of non-telomeric RTEL1 (Fig. 5D and Supplementary Movie 9). These findings suggest that the interactions between RTEL1 and TRF2 contribute substantially to forming long-lasting interactions with telomeres during S-phase. However, we cannot exclude the possibility of an indirect effect, where TRF2 degradation activates a DNA-damage response that then affects RTEL1 recruitment to telomeres.

## Discussion

In this study, we investigated the cellular dynamics and telomere recruitment of RTEL1 in cultured human cancer cells. We found that RTEL1 binding to telomeres increases substantially during S-phase, when this helicase is thought to function in telomere replication, but even then only 5% of chromatin-bound RTEL1 is associated with telomeres. Notably, a mutant RTEL1 lacking ATPase activity still binds normally to chromatin and telomeres, indicating separate mechanisms for its cellular recruitment and enzymatic activity. Our data also support the previously identified RTEL1-TRF2 interaction playing a role in recruiting RTEL1 to telomeres. However, we cannot dismiss the possibility that other chromatin-binding sites of RTEL1 contributed to these observations, because we labeled telomeres with TRF2 so they were not visible after TRF2 degradation. Overall, our study presents a quantitative regulatory model for RTEL1 in cells.

We found that each HeLa cell contains about 3 000 RTEL1 molecules, which is consistent with previous proteomic studies showing 500–4400 RTEL1 molecules per HeLa cell [59, 60]. At any single time during S-phase, one-third of these are chromatin-bound and 5% of those are telomere-bound, which amounts to about 50 RTEL1 molecules (or 25 RTEL1 dimers) bound to the 160 telomeres in a HeLa cell. With an average of less than one bound RTEL1 per telomere, if the binding were long-lived then most telomeres would not even acquire a single RTEL1 molecule. It therefore becomes clear that the rapid dynamics we observe for RTEL1 is essential for this small number of molecules to work on all telomeres. HeLa cells exhibit varying levels of telomere-interacting proteins, ranging from roughly 400 molecules of CTC1 to 200 000 molecules of PRIM2 per cell [59–61] (Supplementary Table S1). The low copy number of RTEL1, in conjunction with the multiple defects in cells harboring the catalytically dead mutant, highlights the essential role of low-abundance proteins such as RTEL1 in telomere preservation and the proliferation of cancer cells.

Our previous research revealed that telomerase, present in only roughly 500 copies per HeLa cell, utilizes three-dimensional diffusion to locate telomeres, probing each telomere thousands of times during the S-phase but only rarely forming stable associations [40]. Similarly, our current research indicates that RTEL1, another low-copy protein, employs a similar mechanism to find its target, suggesting that rapid dynamic diffusion and frequent probing could be shared features for low-abundance proteins. Notably, more than 50% of telomere-associated RTEL1 is stably bound, while only about 4% of telomere-associated telomerase remains highly static in a previous study [40]. This difference may be due to different binding kinetics, stoichiometries, or local protein concentrations at telomeres. A single chromosome terminus can accommodate only one telomerase molecule, yet several RTEL1 molecules likely associate with the same telomere. The oligomeric state of RTEL1 at the telomere is not revealed by our experiments due to the sparse labeling of Halo-RTEL1 that is required to achieve single-molecule imaging. If several RTEL1 molecules bind to a single telomere, our measurements may underestimate the amount of RTEL1 bound to telomeres.

The repetitive sequence of single-stranded telomeric DNA naturally folds into numerous G-quadruplex structures [62, 63], which RTEL1 can actively unfold [54, 64]. Prior single-molecule studies have indicated that various helicases share a common mechanism to unfold G-quadruplex structures, involving repetitive cycles of G-quadruplex unfolding in successive runs [65, 66]. Consequently, a greater stable fraction of RTEL1 at telomeres may enable it to unfold these structures continuously. However, it remains uncertain whether the RTEL1 molecules bound to telomeres are functioning, as catalytically inactive proteins are also recruited to these sites. The specific factors influencing RTEL1 and telomerase at telomeres are also not well understood. Further experiments are necessary to investigate these questions.

We have observed that the ATPase-dead mutant RTEL1 remains associated with telomeres. Due to the ATPase and protein-binding domains being located in different regions of RTEL1 [22], this mutation is unlikely to disrupt the protein-binding modules. Our present study shows that TRF2 degradation significantly decreases RTEL1’s residence time at chromatin. Given that telomeres significantly contribute to long static interactions, TRF2 likely plays a critical role in the telomere recruitment of RTEL1, aligning with previous studies [20]. Nonetheless, TRF2 alone cannot explain the cell-cycle-dependent recruitment of RTEL1, suggesting other factors also regulate the recruitment throughout the cell cycle.

The genome-edited HeLa cells with a catalytically inactive RTEL1 mutant recapitulated some phenotypes previously found in RTEL1-minus HEK293T cells [51] and MEFs [29, 54], but there were some differences. The similarities included severe proliferation defects, shortened telomeres, and metaphase chromosomes with telomeric signal-free ends, indicating that the telomeric DNA is missing (or shortened below the detectable length). In addition, our cell-cycle analysis revealed failure to complete S-phase, and metaphase spreads showed end-to-end telomere fusions. The telomere fusion phenotype of RTEL1 knock-out had been previously reported in mouse ES cells but not in human cells. Thus, RTEL1 has critically important functions that are not compensated by other telomeric helicases such as Werner's or Bloom's.

We have also observed RTEL1 bound to additional chromatin regions in addition to telomeres. The proportion of bound RTEL1 increases during the S-phase, supporting its established DNA replication and other functions during this phase of the cell cycle [67, 68]. Previous research has indicated that DNA secondary structure formation increases during DNA replication and is prevalent in repetitive sequences, such as telomeres [69, 70]. Our current studies demonstrate that RTEL1’s binding to other chromatin regions is more transient than at telomeres. Considering the extended repetitive nature of telomeric DNA compared to other genomic regions, our findings are consistent with the tendency for DNA secondary structure formation to be more pronounced at telomeres.

RTEL1 interacts with various proteins, including SLX4, RPA, PCNA, and TRF2 [54, 67, 71, 72]. It is unclear whether RTEL1 is preassembled as a complex in the free state or entirely assembled at its target sites on chromatin. Halo-RTEL1 has a molecular weight of ∼176 kDa. Our single-molecule live-cell imaging shows that the diffusion coefficient of RTEL1 is ∼1.5 μm²/s, which is lower than that of proteins with a similar molecular weight. For example, Halo-CTCF, ∼160 kDa, has a D_free_ of 2.5 μm²/s [42], and Halo-PARP1, ∼146 kDa, has a D_free_ of 2.9 μm²/s [73]. On the other hand, low molecular weight proteins within a large protein complex have a lower diffusion coefficient. For instance, the PRC2 complex has a molecular weight of ∼320 kDa. Its subunits SUZ12 and EZH2 (each 83–86 kDa as free subunits) have a D_free_ of ∼2 μm²/s in cells [74], consistent with their residing in a preassembled complex. The Shieldin complex has a molecular weight of 170 kDa and often interacts with 53BP1–Rif1 at DNA double-strand breaks. The diffusion coefficient of Shieldin subunits varies from 1.6 to 3.7 μm²/s [75], reflecting partially preassembled complexes. Thus, the low diffusion coefficient of RTEL1 suggests that it is preassembled with partner proteins or exists as a dimer in the free state. However, we cannot exclude the possibility that an extended protein shape could contribute to its low D value.

RTEL1 was first identified in gastrointestinal cancer patients [24]. However, no small molecule specifically targeting this protein has been reported thus far. Our study shows that inhibiting RTEL1 ATPase activity can significantly slow cancer cell growth, offering a promising mechanism of action for future RTEL1-targeted treatments. Nevertheless, long-term treatment with such an inhibitor should be approached with caution, given the adverse effects noted in inherited diseases linked to RTEL1 mutations.

In summary, our study presents a new model for the dynamic recruitment of RTEL1 to telomeres, demonstrates the importance of its ATPase activity in cancer cell survival, and proposes a strategy for targeting RTEL1 for cancer intervention.

## Supplementary Material

gkaf177_Supplemental_Files

## Data Availability

The data underlying this article are available in the article and in its online supplementary material. The uncropped gel images are provided in the Supplementary Fig. S12.

## Notes

Present address: University of California San Diego, La Jolla, CA, 92093, USA
