# Supplementary material for: Rapid dynamics allow the low-abundance RTEL1 helicase to promote telomere replication
Source: Nucleic Acids Res. 2025 Mar 13;53(5):gkaf177. doi: 10.1093/nar/gkaf177 (PMC11909005; doi:10.1093/nar/gkaf177)
Supplement: gkaf177_Supplemental_Files [file gkaf177_supplemental_files.zip › RTEL1_Revision_Supplementary Data.pdf]

Supplementary Data for

Rapid dynamics allow the low-abundance RTEL1 helicase to promote telomere replication

Guanhui Wu[1,3,4], Erin Taylor[2], Daniel T. Youmans[1,3,4], Nausica Arnoult[2], and Thomas R. Cech[1,3,4]*

[1] Department of Biochemistry, University of Colorado Boulder, Boulder, CO 80303, USA
[2] Department of Molecular, Cellular, and Developmental Biology, University of Colorado Boulder, Boulder, CO 80303, USA
[3] BioFrontiers Institute, University of Colorado Boulder, Boulder, CO 80303, USA
[4] Howard Hughes Medical Institute, University of Colorado Boulder, Boulder, CO 80303, USA
* To whom correspondence should be addressed. Email: thomas.cech@colorado.edu

Present Address: Daniel T. Youmans, University of California San Diego, La Jolla, CA, 92093, USA

This file includes:

Supplementary Figures 1 to 12

Supplementary Tables 1 to 2

Legends for Supplementary Movies 1 to 9

Supplementary Donor Vector Sequences

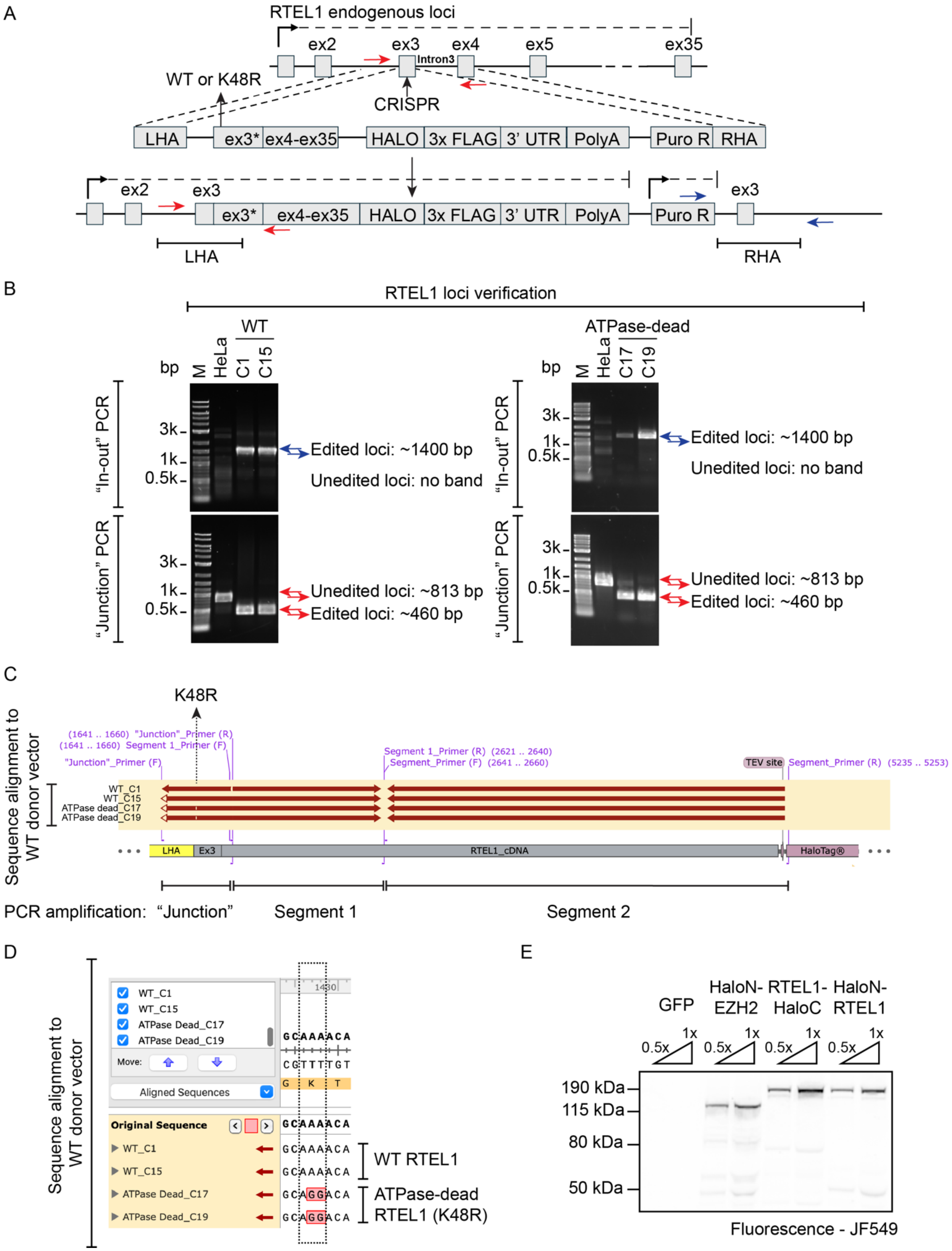
A
RTEL1 endogenous loci
ex2
ex3
ex4
ex5
ex35
Intron3
WT or K48R
CRISPR
LHA
ex3*
ex4-ex35
HALO
3x FLAG
3' UTR
PolyA
Puro R
RHA
B
RTEL1 loci verification
WT
ATPase-dead
bp
M
HeLa
C1
C15
C17
C19
3k
1k
0.5k
"In-out" PCR
"Junction" PCR
Edited loci: ~1400 bp
Unedited loci: no band
Unedited loci: ~813 bp
Edited loci: ~460 bp
C
K48R
Sequence alignment to WT donor vector
WT_C1
WT_C15
ATPase dead_C17
ATPase dead_C19
LHA
Ex3
RTEL1_cDNA
TEV site
HaloTag®
PCR amplification: "Junction"
Segment 1
Segment 2
D
Sequence alignment to WT donor vector
WT_C1
WT_C15
ATPase Dead_C17
ATPase Dead_C19
Move:
Aligned Sequences
Original Sequence
WT RTEL1
ATPase-dead RTEL1 (K48R)
E
GFP
HaloN-EZH2
RTEL1-HaloC
HaloN-RTEL1
0.5x
1x
190 kDa
115 kDa
80 kDa
50 kDa
Fluorescence - JF549

Supplementary Figure 1. Generation and validation of doubly genome-edited cell lines that express WT or ATPase-dead (K48R) RTEL1.

(**A**) Schematic representation of the CRISPR genome-editing and PCR verification strategies for inserting FLAG-HaloTag into the endogenous loci of RTEL1. Red arrows indicate the binding sites of "junction" PCR primers, while blue arrows indicate the binding sites of "in-out" PCR primers. LHA and RHA indicate the left and right homology arms, respectively. ex3*, part of exon 3, which is codon-optimized. (**B**) Agarose gels of PCR products amplified from genomic DNA of unedited HeLa cells and genome-edited clones using the indicated primers. M, DNA molecular weight markers. (**C**) A window obtained from SnapGene displaying multiple PCR products' alignment with the wildtype donor vector. (**D**) DNA sequences obtained from the "junction" PCR products of the indicated clones. The desired mutation is highlighted using rectangle dashed lines. (**E**) SDS-PAGE of fluorescently labeled proteins from HEK293T cells following the transient transfection of specified protein constructs: GFP as a negative control, N-terminal Halo-tagged EZH2 (HaloN-EZH2) as a positive control, C-terminal Halo-tagged RTEL1 (RTEL1-HaloC), and N-terminal Halo-tagged RTEL1 (HaloN-RTEL1).

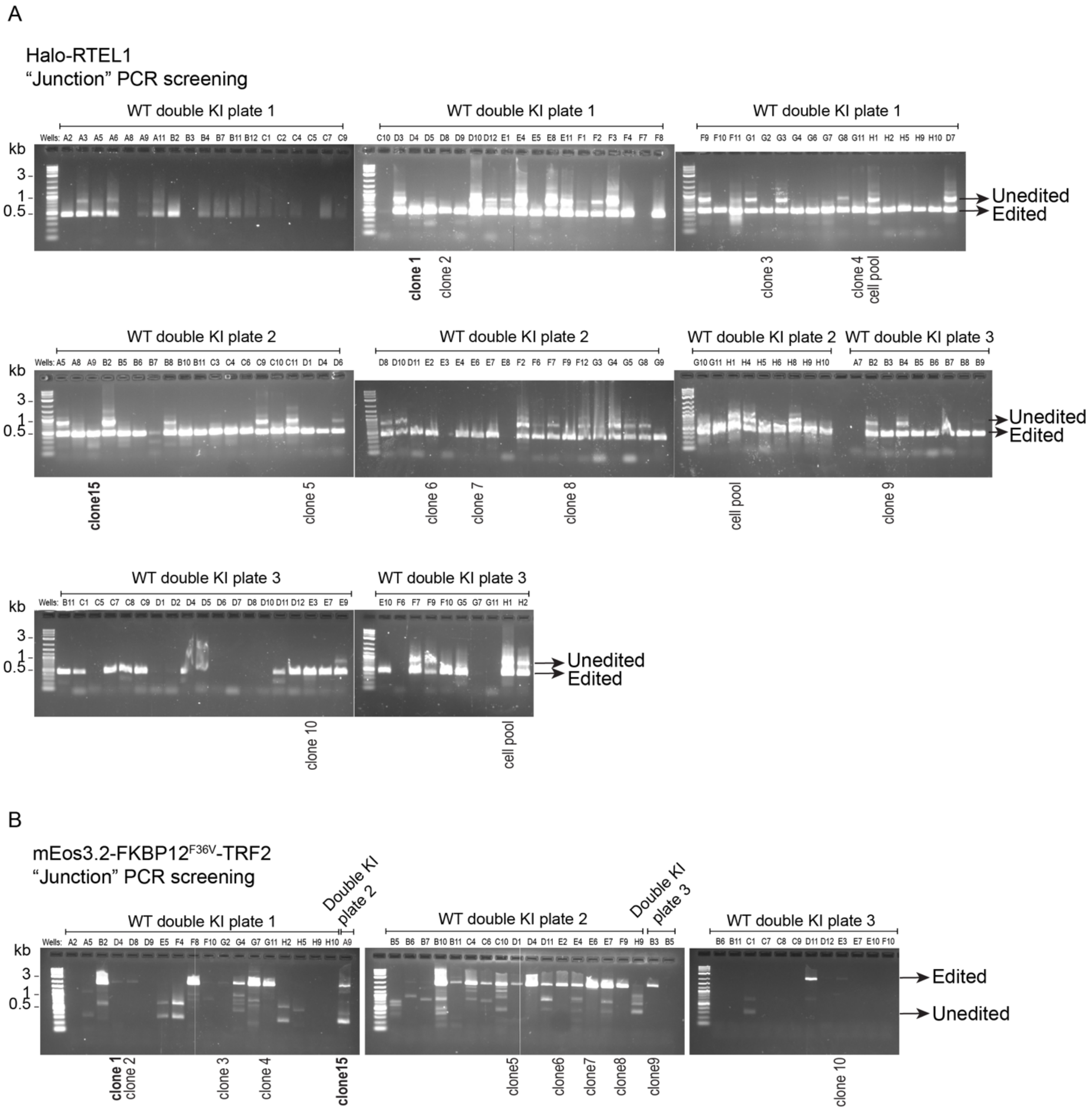

Supplementary Figure 2. Results of "junction" PCR screening for doubly genome-edited cell lines expressing WT Halo-RTEL1 and HA-mEos3.2-FKBP12^{F36V}-TRF2.

(**A**) Agarose gels of PCR products amplified from genome-edited clones using RTEL1 "junction" PCR primers (see Supplementary Figure 1 and Supplementary Table 2 for details). KI, knock in. (**B**) Agarose gels of PCR products amplified from genome-edited clones using TRF2 "junction" PCR primers (see Supplementary Figure 3 and Supplementary Table 2 for details).

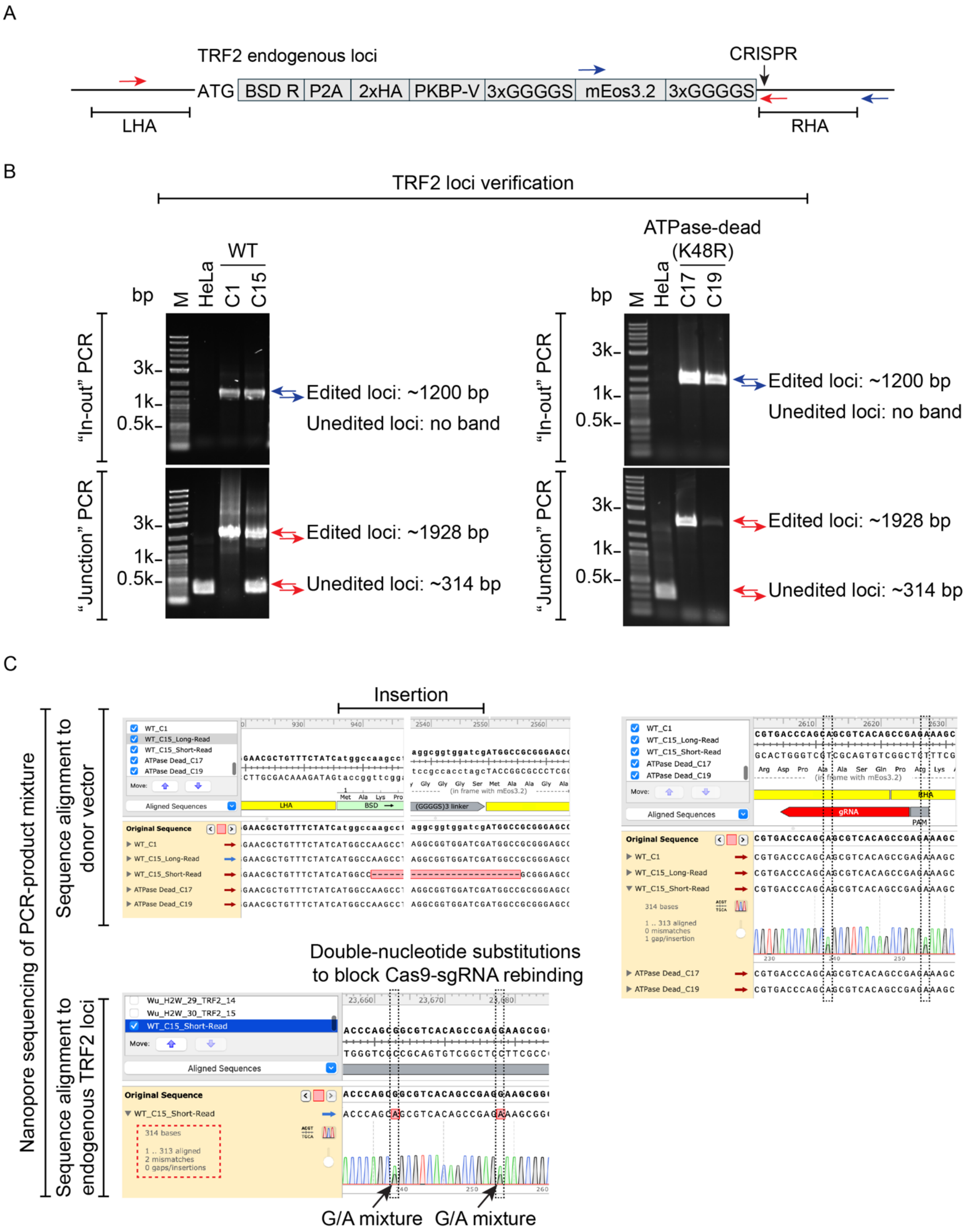
A
TRF2 endogenous loci
CRISPR
ATG
BSD R
P2A
2xHA
PKBP-V
3xGGGGS
mEos3.2
3xGGGGS
LHA
RHA
B
TRF2 loci verification
WT
ATPase-dead (K48R)
bp
M
HeLa
C1
C15
C17
C19
3k
1k
0.5k
"In-out" PCR
"Junction" PCR
Edited loci: ~1200 bp
Unedited loci: no band
Edited loci: ~1928 bp
Unedited loci: ~314 bp
C
Insertion
Nanopore sequencing of PCR-product mixture
Sequence alignment to donor vector
Sequence alignment to endogenous TRF2 loci
Double-nucleotide substitutions to block Cas9-sgRNA rebinding
G/A mixture
G/A mixture

Supplementary Figure 3. Generation and validation of doubly genome-edited cell lines that express HA-mEos3.2-FKBP12^{F36V}-TRF2.

(**A**) Schematic representation of the CRISPR genome-editing and PCR verification strategies for inserting the HA-mEos3.2-FKBP12^{F36V} tag into the endogenous loci of TRF2. Red arrows indicate the binding sites of "junction" PCR primers, while blue arrows indicate the binding sites of "in-out" PCR primers. LHA and RHA indicate the left and right homology arms, respectively. 3×GGGGS is a 15-amino acid flexible linker of the indicated sequence. (**B**) Agarose gels of PCR products amplified from genomic DNA of genome-edited clones using the indicated primers. (**C**) DNA sequences obtained from the "junction" PCR products of the indicated clones. Two silent mutations were added at the sgRNA binding sites to prevent Cas9-sgRNA rebinding and enhance editing efficiency. These mutations are highlighted using black dotted line rectangles. The sequence alignment results for endogenous TRF2 are highlighted using a red dotted line rectangle.

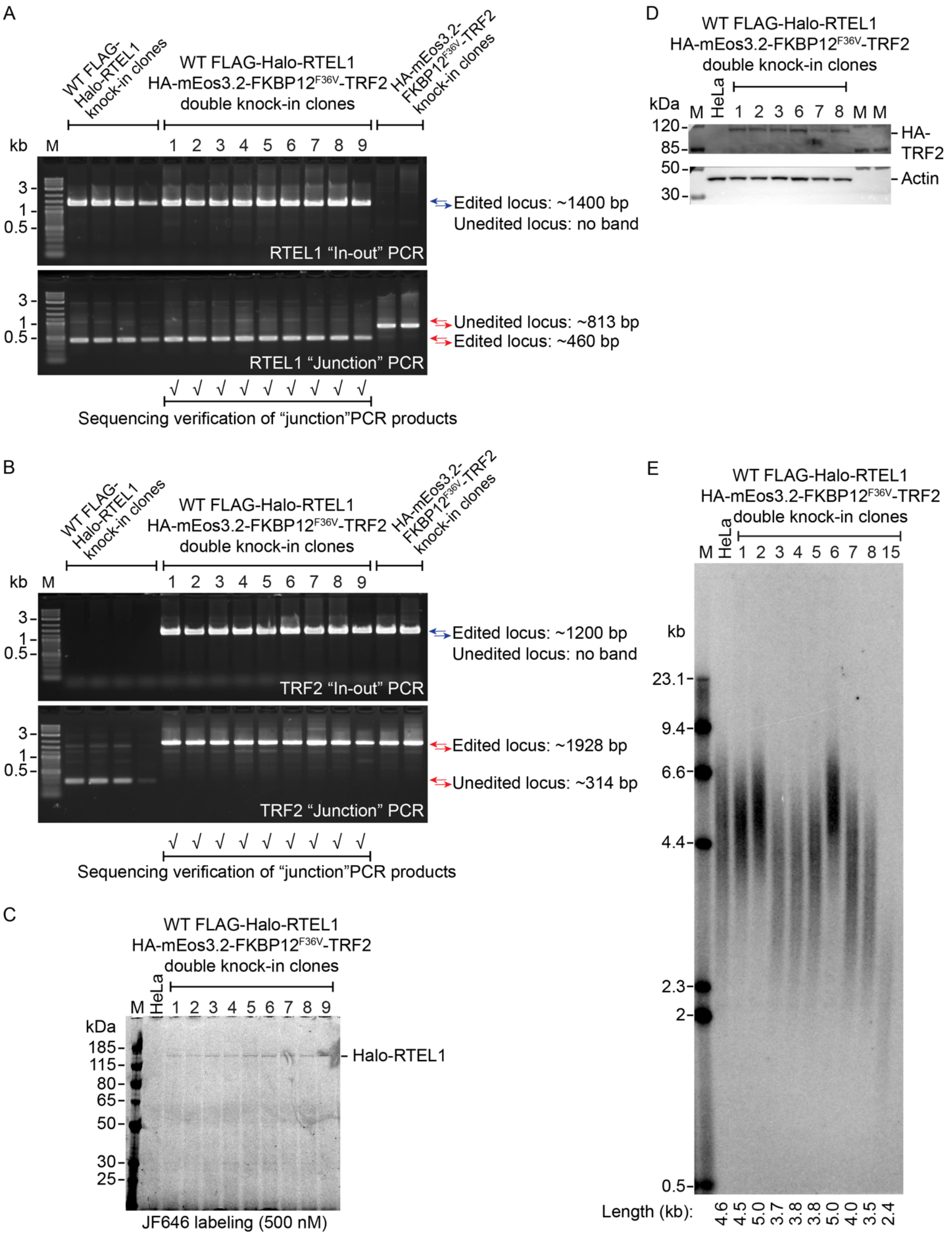
A
WT FLAG-Halo-RTEL1 knock-in clones
WT FLAG-Halo-RTEL1
HA-mEos3.2-FKBP12F36V-TRF2
double knock-in clones
HA-mEos3.2-FKBP12F36V-TRF2 knock-in clones
kb M 1 2 3 4 5 6 7 8 9
3
1
0.5
Edited locus: ~1400 bp
Unedited locus: no band
RTEL1 "In-out" PCR
Unedited locus: ~813 bp
Edited locus: ~460 bp
RTEL1 "Junction" PCR
Sequencing verification of "junction"PCR products
B
Edited locus: ~1200 bp
Unedited locus: no band
TRF2 "In-out" PCR
Edited locus: ~1928 bp
Unedited locus: ~314 bp
TRF2 "Junction" PCR
Sequencing verification of "junction"PCR products
C
M HeLa 1 2 3 4 5 6 7 8 9
kDa
185
115
80
65
50
30
25
Halo-RTEL1
JF646 labeling (500 nM)
D
kDa M HeLa 1 2 3 6 7 8 M M
120
85
50
30
HA-TRF2
Actin
E
M HeLa 1 2 3 4 5 6 7 8 15
kb
23.1
9.4
6.6
4.4
2.3
2
0.5
Length (kb): 4.6 4.5 5.0 3.7 3.8 3.8 5.0 4.0 3.5 2.4

Supplementary Figure 4. Characterization of doubly genome-edited cell lines that express WT FLAG-Halo-RTEL1 and HA-mEos3.2-FKBP12^{F36V}-TRF2.

(**A**) Agarose gels of RTEL1 PCR products amplified from genomic DNA of genome-edited clones using the indicated primers (see Supplementary Figure 1 and Supplementary Table 2 for details). (**B**) Agarose gels of TRF2 PCR products amplified from genomic DNA of genome-edited clones using the indicated primers (see Supplementary Figure 3 and Supplementary Table 2 for details). (**C**) SDS-PAGE of fluorescently labeled Halo-RTEL1 from doubly genome-edited clones. (**D**) Western blot of HA-mEos3.2-FKBP12^{F36V} tagged TRF2 from doubly genome-edited clones using an anti-HA antibody. (**E**) Telomeric restriction fragment Southern blot of genomic DNA from doubly genome-edited clones.

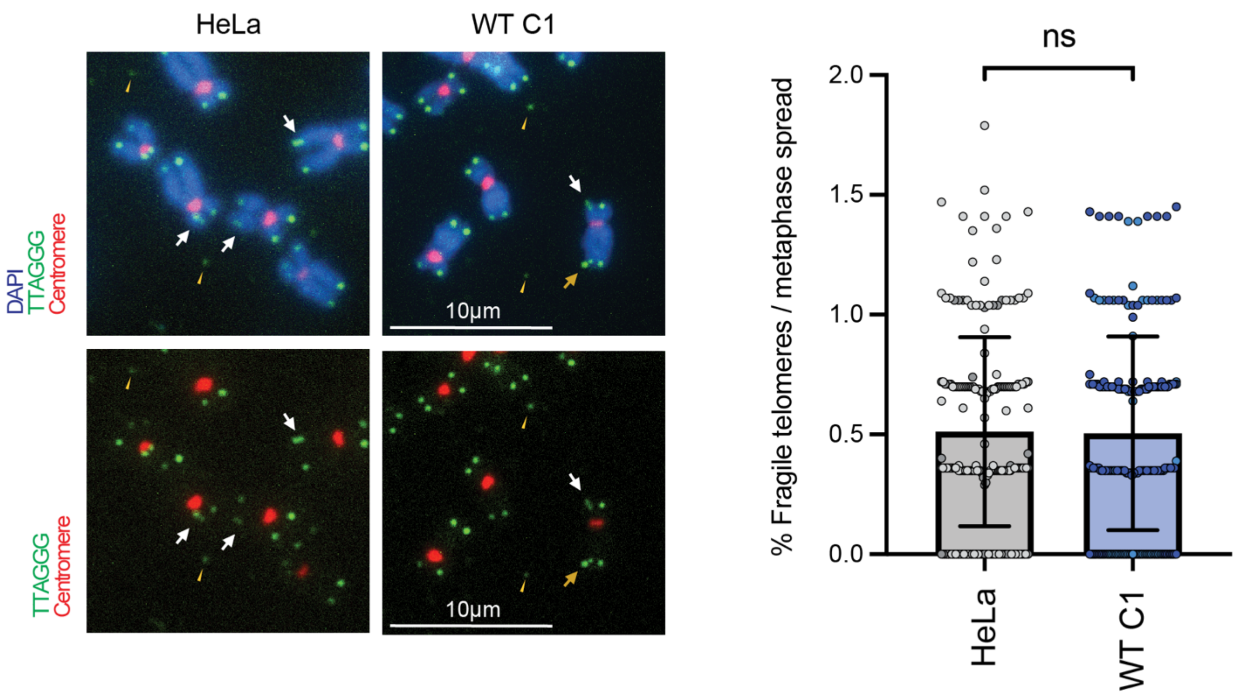

Supplementary Figure 5. Metaphase spreads analysis of doubly genome-edited cell lines for quantifying fragile telomeres.

Representative images and quantifications of fragile telomeres for metaphase spreads from parental HeLa cells and Halo-RTEL1 WT clone 1. Bottom images, DAPI not included to facilitate visualization of distortions or blebbing (white arrows) or multiple distinct puncta (yellow arrows) of telomere-FISH signals that were quantified as fragile telomeres. Note that it cannot be excluded that some percentages of these events are the result of punctate background signal (yellow wedges) colocalizing at chromosome ends, and as such background levels of fragility may be elevated. n=193-200 metaphases across 2 replicates. N1= darker colored data points & N2= lighter colored data points. Error bars are mean +/- SD. ns, non-significant by unpaired t-test (p=0.8824).

A

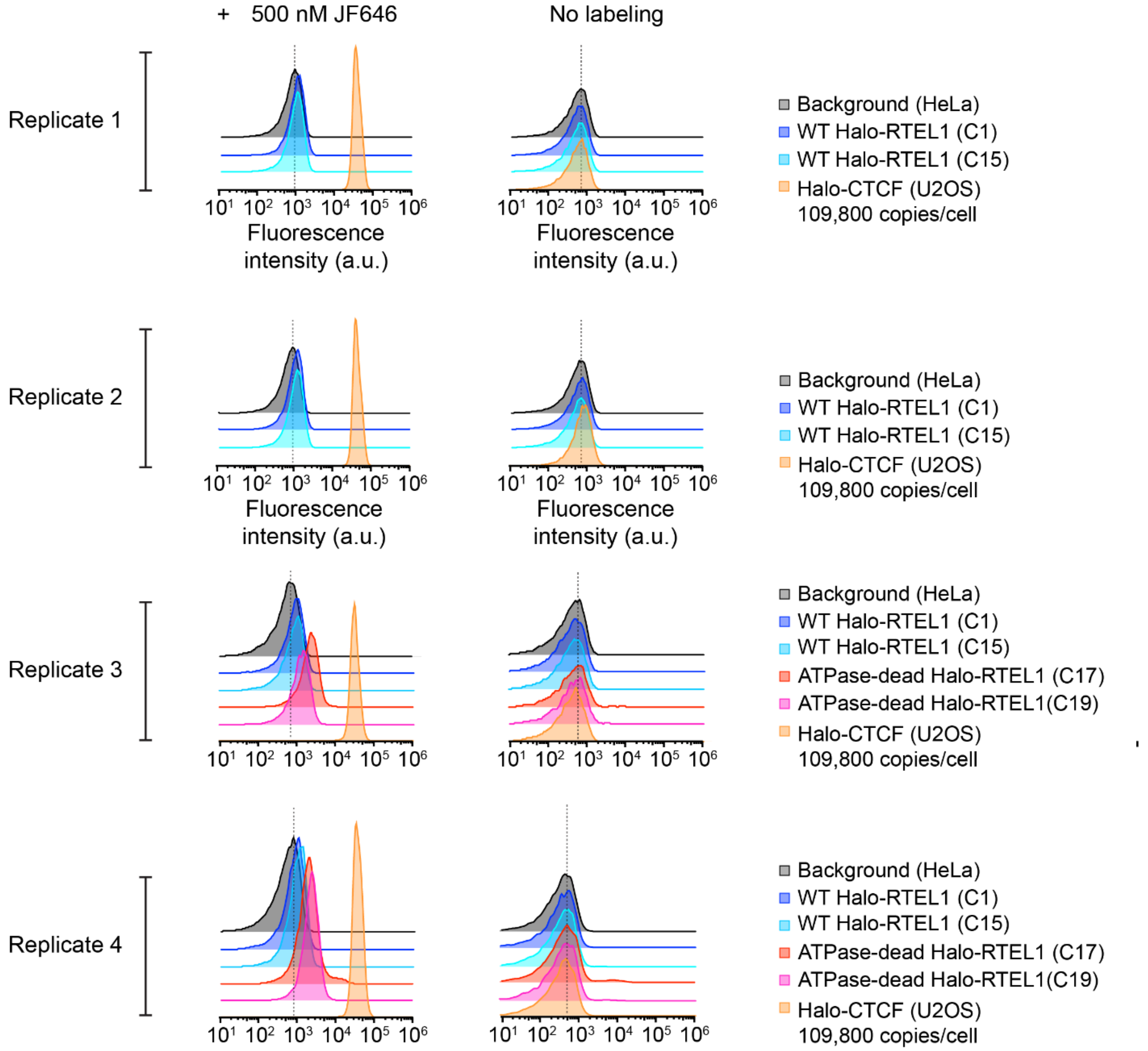

B

	MFI (no labeling)				MFI (500 nM JF646)				MFI (500 nM JF646 - no lableling)				MFI (background subtraction)				Halo (molecules/cell)				Total RTEL1 (molecules/cell, corrected by WB)				AVG
Background (HeLa)	529	542	455	417	845	782	643	641	316	240	188	224	0	0	0	0	0	0	0	0					
WT (C1)	503	556	452	428	1114	1110	944	922	611	554	492	494	295	314	304	270	831	790	1025	773	2866	2724	3533	2664	2947
WT (C15)	508	527	476	438	1037	1150	925	1094	529	623	449	656	213	383	261	432	600	964	880	1236	2055	3300	3013	4233	3150
ATPase-dead (C17)	NA	NA	731	750	NA	NA	2447	2561			1716	1811			1528	1587			5151	4541			5151	4541	4846
ATPase-dead (C19)	NA	NA	584	537	NA	NA	1393	2464			809	1927			621	1703			2094	4873			2094	4873	3483
Halo-CTCF (U2OS)	543	883	437	425	39825	44759	33194	39022	39282	43876	32757	38597	38966	43636	32569	38373	109800 (Reference)								

MFI: mean fluorescence intensity

Supplementary Figure 6. Quantification of the absolute copy number of RTEL1 protein in genome-edited cell lines.

(**A**) Flow cytometry analysis of JF646-labeled cell lines that express WT Halo-RTEL1, ATPase-dead Halo-RTEL1, or Halo-CTCF. The absolute copy number of Halo-CTCF was previously determined to be approximately 109,800 copies per U2OS cell. n > 15000 cells. (**B**) Absolute copy number of WT Halo-RTEL1, ATPase-dead Halo-RTEL1, and total RTEL1 per cell from genome-edited clones. "Corrected by WB" (Western Blot) was performed for the WT Halo-RTEL1 to account for the non-Halo-tagged RTEL1 that migrated below the Halo-RTEL1; this species was not seen with ATPase-dead Halo-RTEL1, so no correction was needed. AVG, average. The detailed calculation method has been included in the methods section.

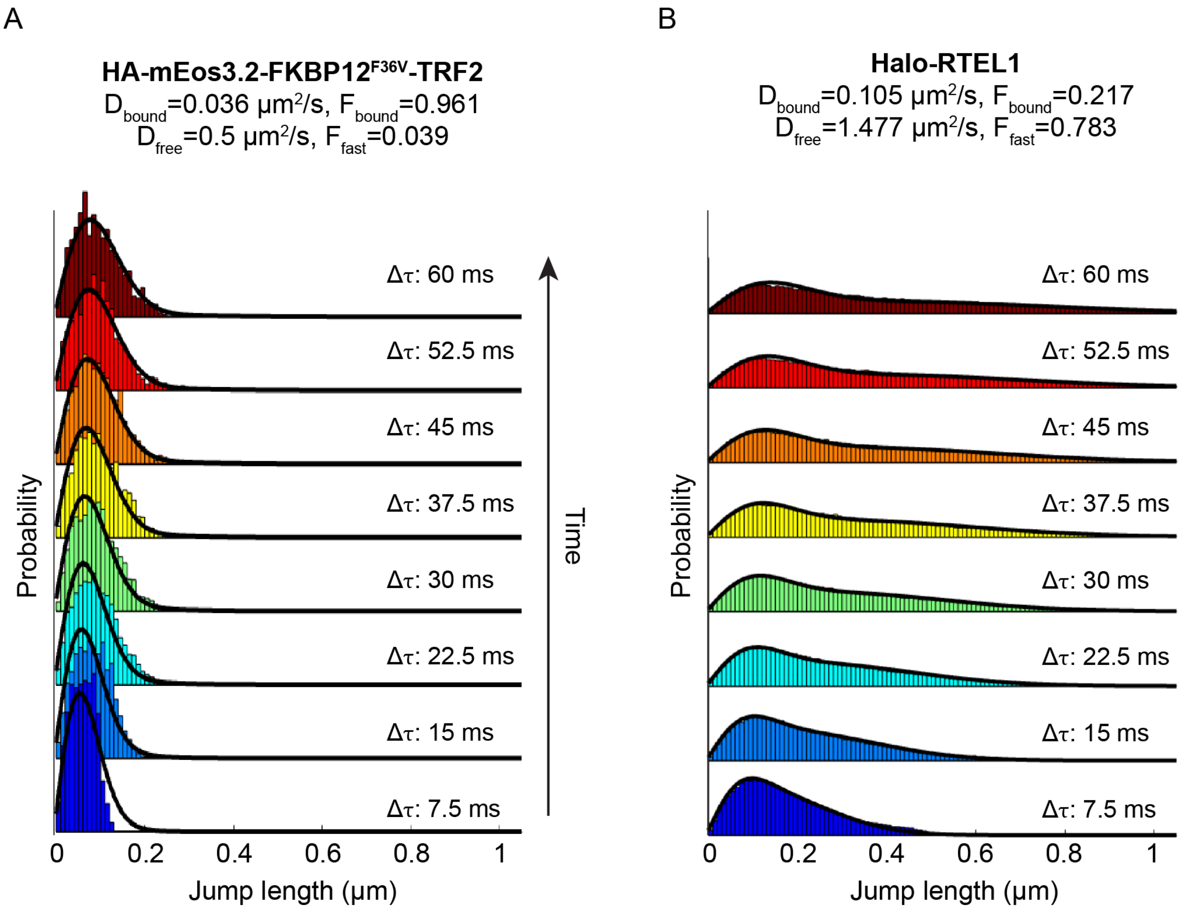

Supplementary Figure 7. Spot-on jump length distribution plots for HA-mEos3.2-FKBP12^{F36V}-TRF2 and Halo-RTEL1.

Probability density functions of the jump lengths derived from HA-mEos3.2-FKBP12^{F36V}-TRF2 (A) and Halo-RTEL1 (B) trajectories and the corresponding two-state model fit using the Spot-On software. WT C1 cell line (asynchronous state) was used in the analyses. *n* >50 cells.

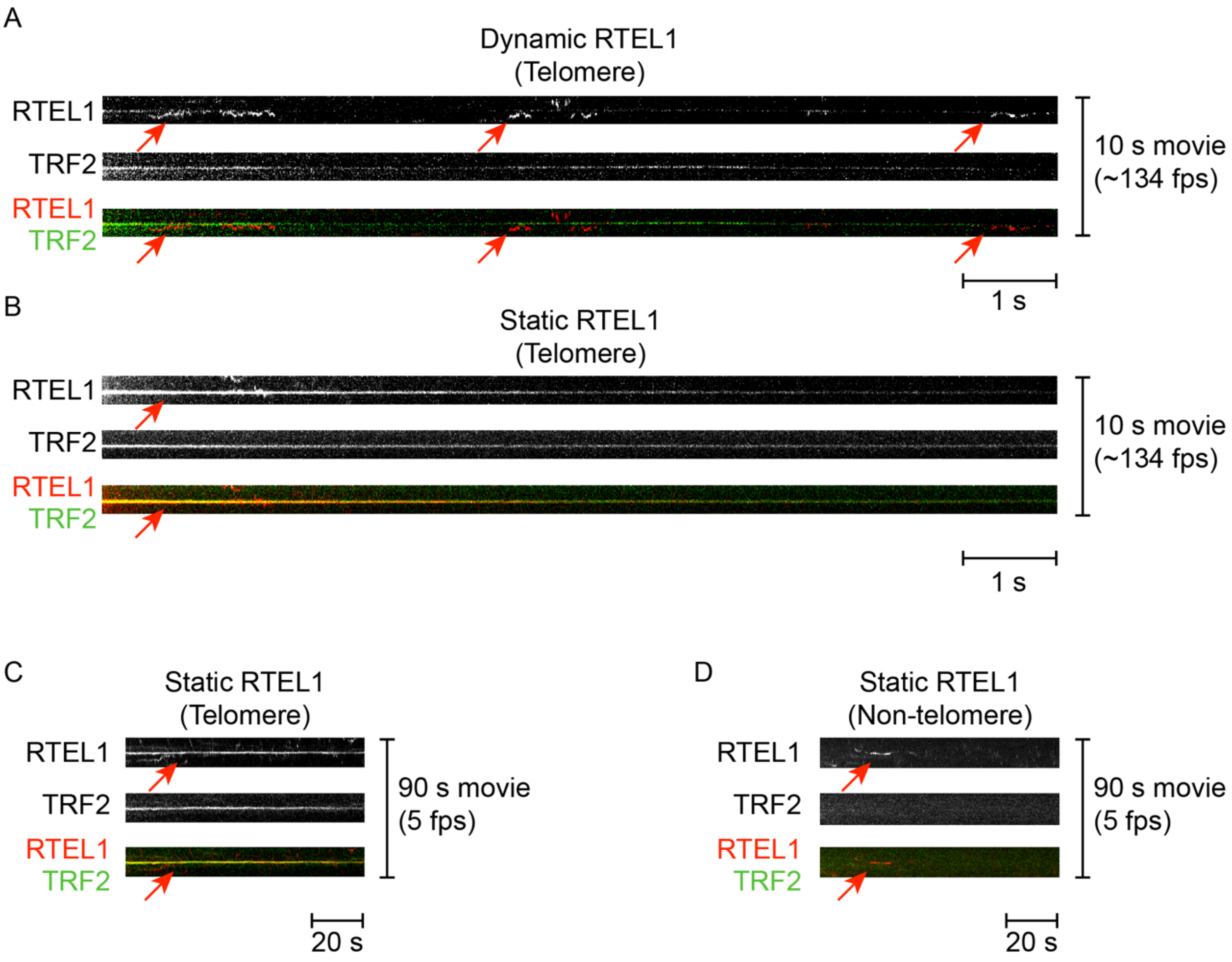

Supplementary Figure 8. Kymographs of dynamic and static RTEL1.

(**A**) Dynamic RTEL1 tracks overlapping with the TRF2 signals. The 10-second video was recorded at approximately 134 fps. Red arrows mark the beginning of binding events. (**B**) A static RTEL1 track overlapping with the TRF2 signals. The 10-second video was recorded at approximately 134 fps. (**C**) A static RTEL1 track overlapping with the TRF2 signals. The 90-second video was recorded at 5 fps. (**D**) A static RTEL1 track not overlapping with the TRF2 signals. The 90-second video was recorded at 5 fps.

Replicate 1:

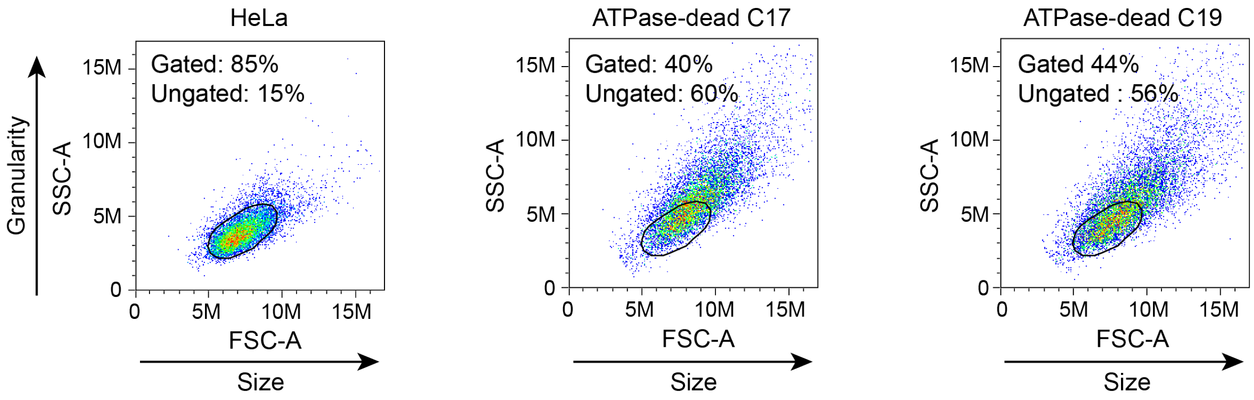

Replicate 2:

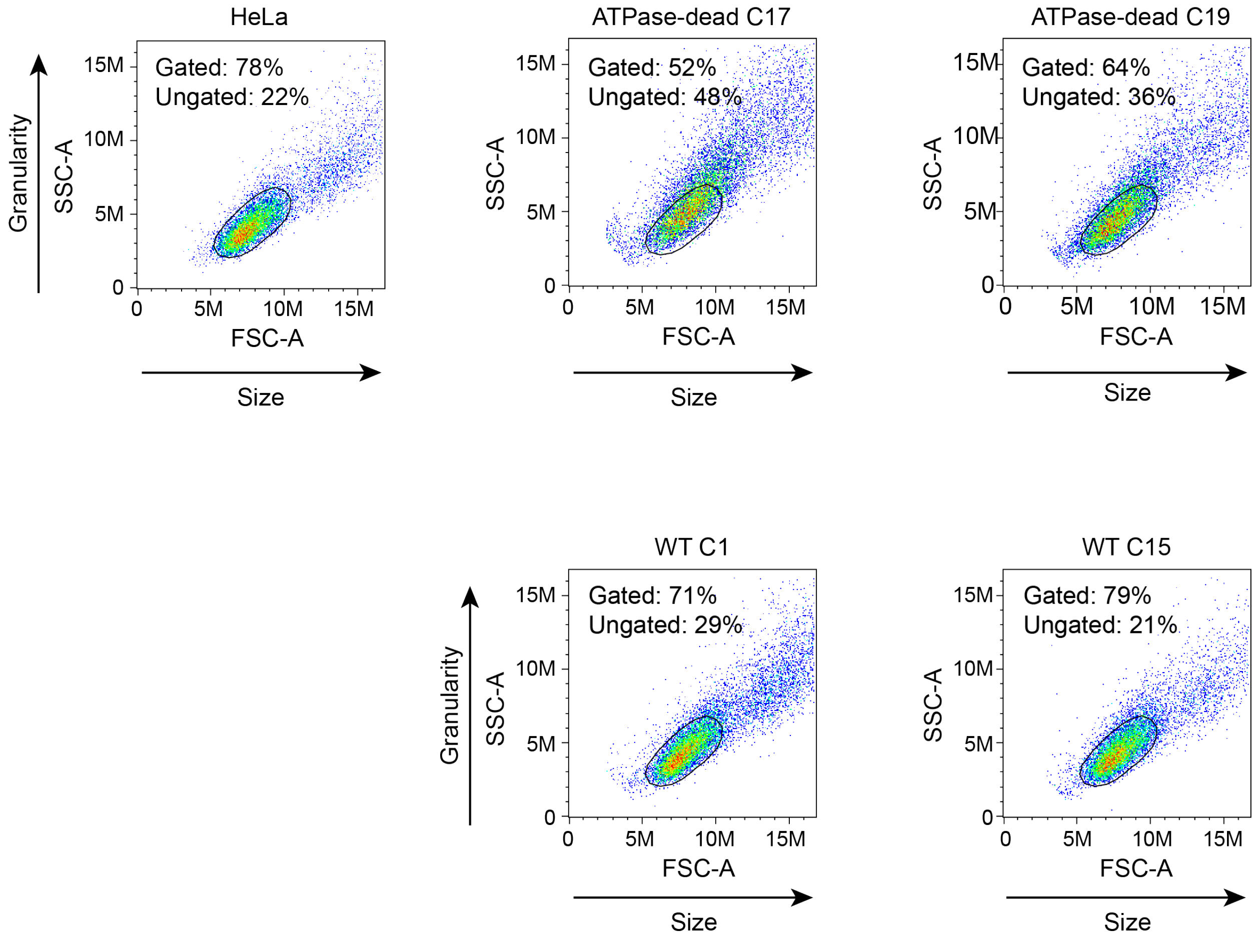

Supplementary Figure 9. Comparison of cell granularity and size of parental HeLa and doubly genome-edited cell lines.

Flow cytometry analysis of cell granularity and size of parental HeLa and doubly genome-edited cell lines. The same gating window was used for each replicate to determine the percentage of cells with similar cell granularity and size. The percentages of the gated and ungated subsets are shown in figures. $n > 10000$ cells per condition.

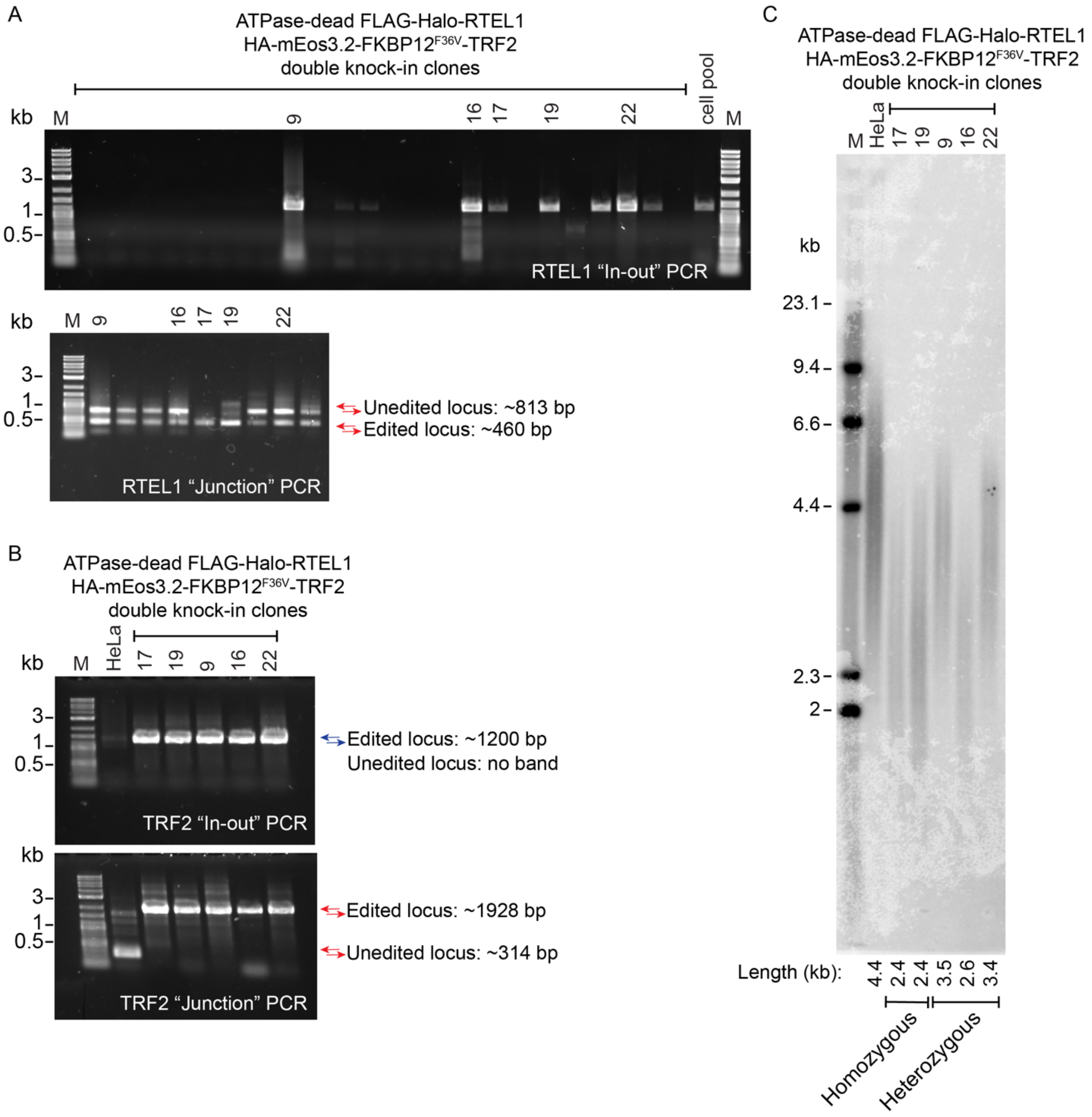

Supplementary Figure 10. Characterization of doubly genome-edited cell lines that express ATPase-dead (K48R) FLAG-Halo-RTEL1 and HA-mEos3.2-FKBP12^{F36V}-TRF2.

(**A**) Agarose gels of RTEL1 PCR products amplified from genomic DNA of genome-edited clones using the indicated primers (see Supplementary Figure 1 and Supplementary Table 2 for details). (**B**) Agarose gels of TRF2 PCR products amplified from genomic DNA of genome-edited clones using the indicated primers (see Supplementary Figure 3 and Supplementary Table 2 for details).

(**C**) Telomeric restriction fragment Southern blot of genomic DNA from the doubly genome-edited clones.

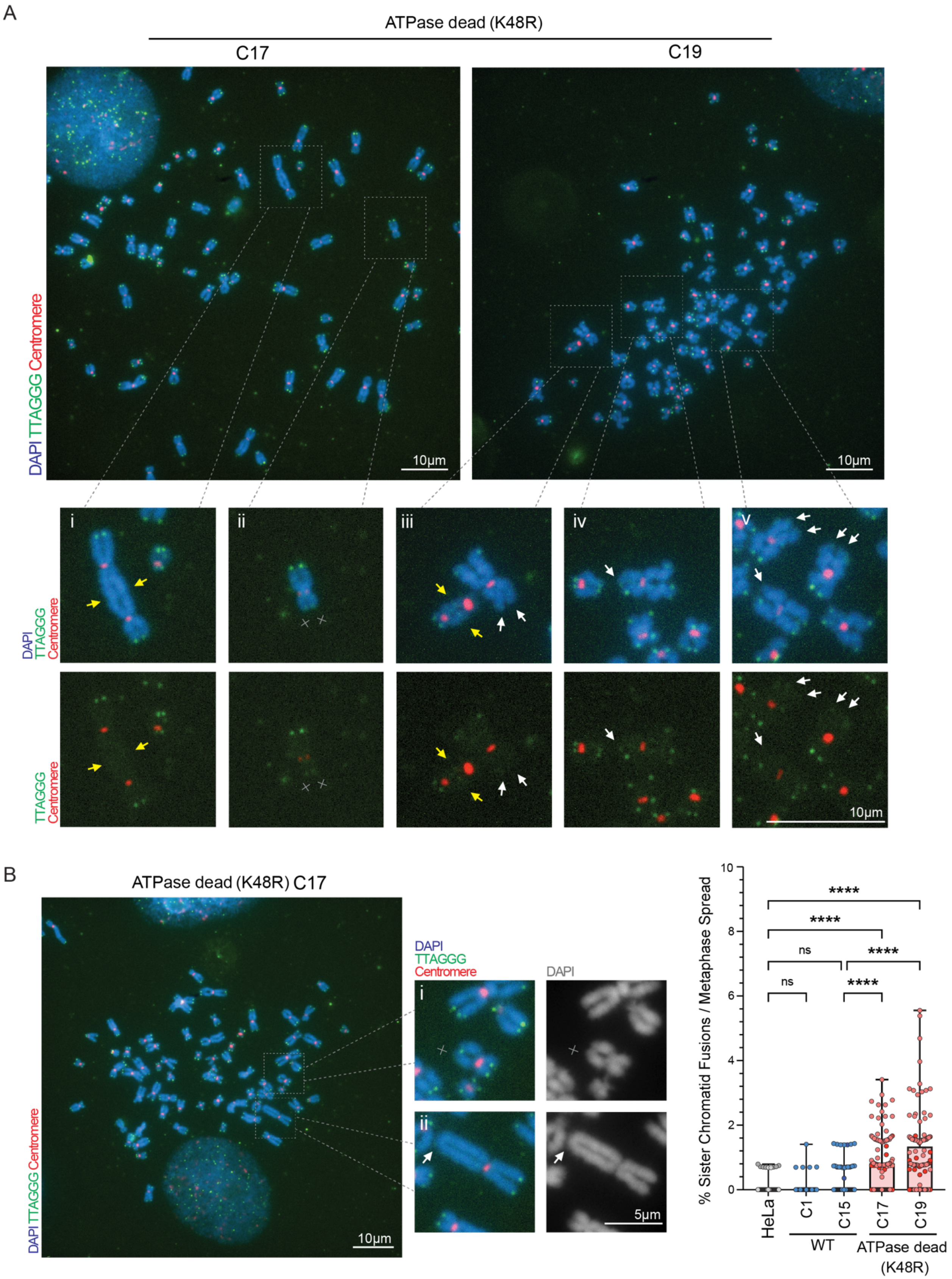

A
ATPase dead (K48R)
C17
C19
DAPI TTAGGG Centromere
10µm
10µm
i
ii
iii
iv
v
DAPI TTAGGG Centromere
TTAGGG Centromere
10µm
B
ATPase dead (K48R) C17
DAPI TTAGGG Centromere
10µm
DAPI
TTAGGG
Centromere
DAPI
i
ii
5µm
% Sister Chromatid Fusions / Metaphase Spread
10
8
6
4
2
0
****
****
ns
****
ns
****
HeLa
C1
C15
C17
C19
WT
ATPase dead (K48R)

Supplementary Figure 11. Metaphase spreads analysis of doubly genome-edited cell lines that express ATPase-dead (K48R) FLAG-Halo-RTEL1 and HA-mEos3.2-FKBP12^{F36V}-TRF2.

(**A**) Representative images of metaphase spreads. End-to-end fusions were identified and counted if they resulted in a dicentric chromosome (yellow arrows in panels i, iii). Chromosome ends were classified as signal-free if no telomeric FISH signal was detectable (white arrows in panels iii, v), or if a very dim signal was significantly lower than the signal on the sister chromatid (white arrow in panel iv). However, dim signals were not considered signal-free ends if they were equivalent between sister chromatids (x's in panel ii). (**B**) Representative images and quantifications of sister-chromatid fusions. Fusion events were identified as bridge-like structures between sister chromatids, with one or no telomeric FISH signal and no detectable gap in the DAPI signal (white arrow in panel ii). Conversely, any evidence of separation between chromatids, as observed in grayscale DAPI images, indicated a signal-free end rather than a fusion event (x in panel i). n=193-201 metaphases per condition across 2 replicates, N1=darker colored and N2=lighter colored data points. The box represents the interquartile range (50th–75th percentiles), while the bars indicate the upper quartile range. ns, non-significant, ****$p <$ 0.0001 was calculated by Sidak test after one-way ANOVA.

Figure 1B

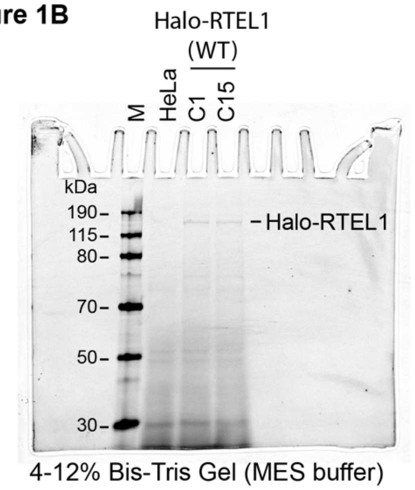

Figure 1D

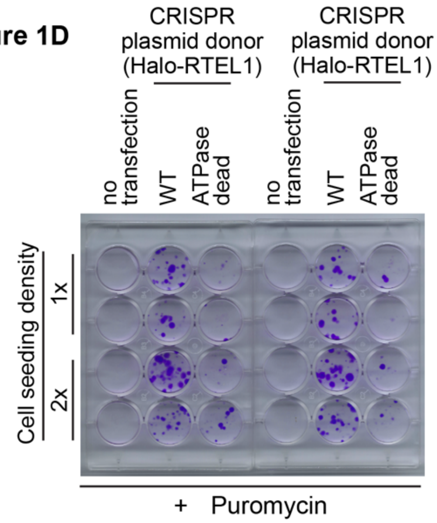

Figure 1C

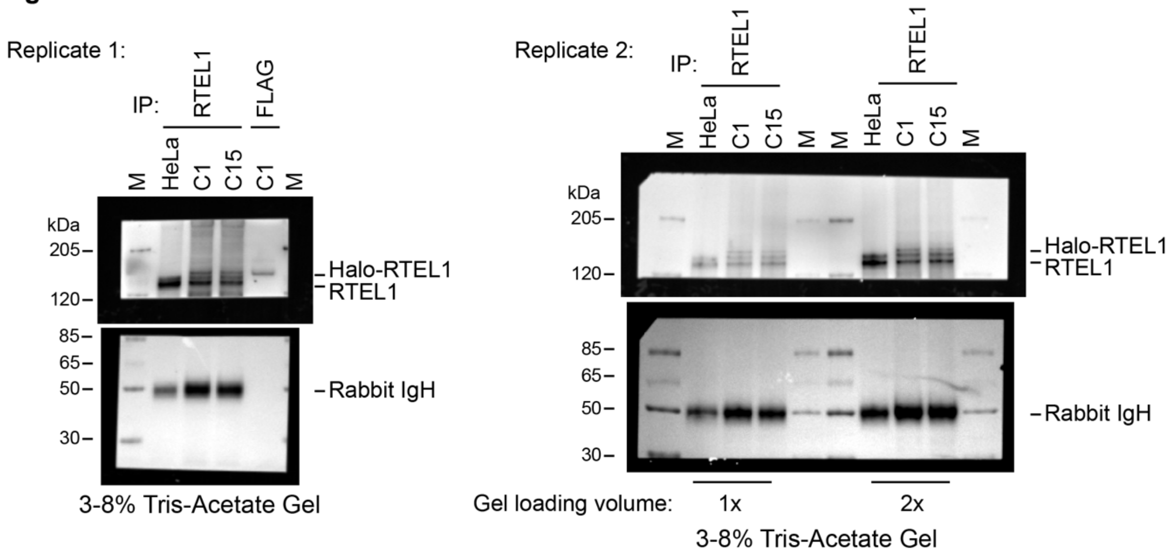

Figure 1E

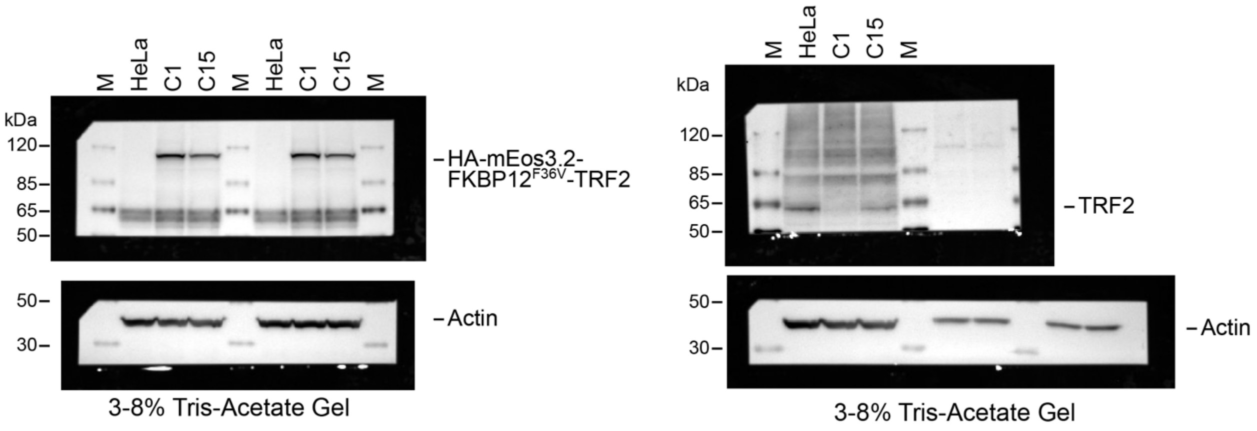

Supplementary Figure 12. Uncropped images

Figure 3A

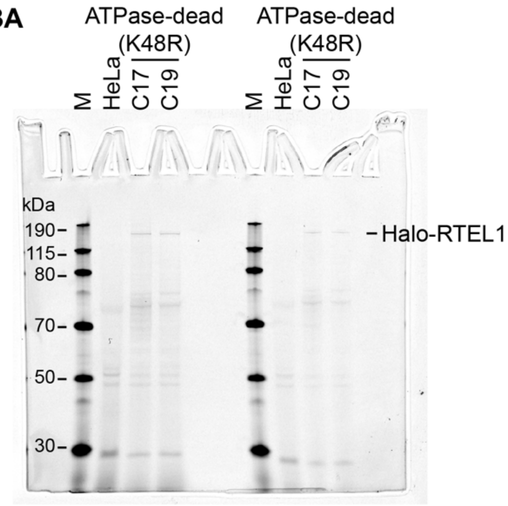

4-12% Bis-Tris Gel (MES buffer)

Figure 3B

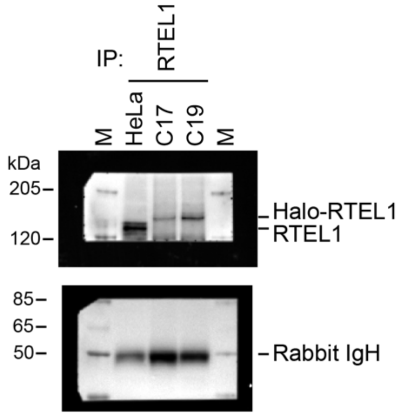

3-8% Tris-Acetate Gel

Figure 3C

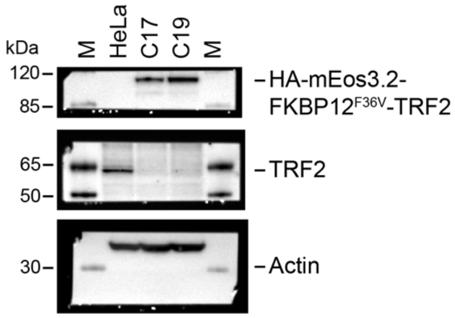

3-8% Tris-Acetate Gel

Figure 3D

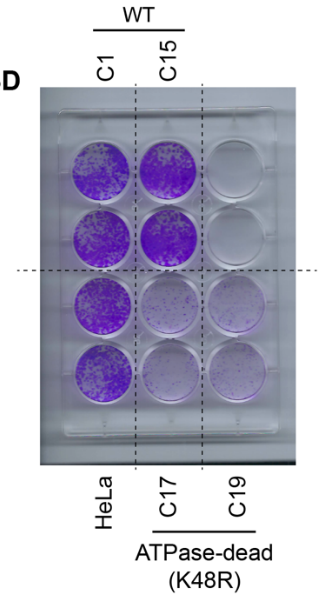

Figure 5B

Replicate 1:

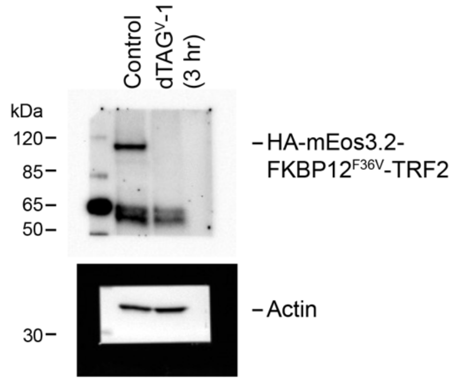

3-8% Tris-Acetate Gel

Replicate 2:

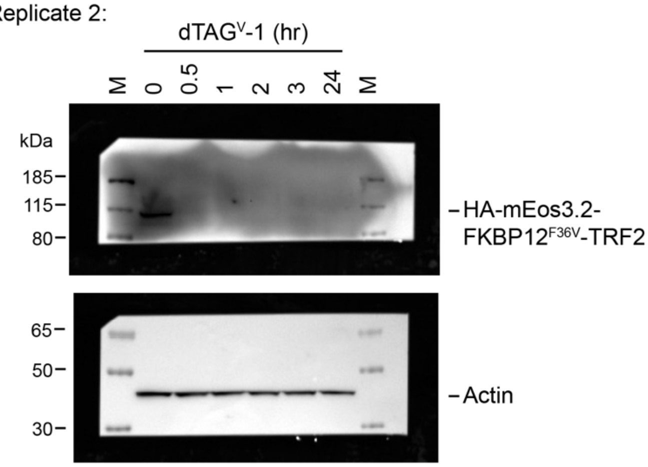

4-12% Bis-Tris Gel (MOPS buffer)

Supplementary Figure 12. Uncropped images (continued)

Gene name	Copy numbers per HeLa cell	
TERT	~500 (1)	Telomerase complex
TERC	~1150 (1)	
TERF1 (TRF1)	1704 (2), 2881 (3)	Shelterin complex
TERF2 (TRF2)	31394 (2), 61285 (3)	
POT1	1438 (2), 11821 (3)	
ACD (TPP1)	6227 (2), 20938 (3)	
TERF2IP (RAP1)	86725 (2), 60692 (3)	
TINF2 (TIN2)	3363 (3)	
CTC1	420 (2), 507 (3)	CST complex
OBFC1 (STN1)	8215 (3)	
TEN1	9458 (3)	
POLA1	35724 (2), 56102 (3)	Polα complex
POLA2	79300 (2), 105356 (3)	
PRIM1	65642 (2), 193031 (3)	Primase complex
PRIM2	96306 (2), 268421 (3)	
WRN	842 (2), 10027 (3)	Telomere-interacting helicases
BLM	6651 (2), 26053 (3)	
RIF1	10182 (2), 64724 (3)	
RTEL1	4434 (2), 543 (3)	
RTEL1	~3000 (this study)	

Supplementary Table 1. Absolute copy numbers of the proteins/RNA found in telomere-interacting complexes and helicases that interact with telomeres

		Sequence (5' to 3')
Guide sequence inserts	RTEL1	GCGTCTTCCCTGTACCCGTA
	TRF2	ACCCAGCGGCGTCACAGCCG
Junction PCR primers	RTEL1	CCTTTAGGGCAAGAGTGGGA
		GCTCGTTGATGACCTGTGTG
	TRF2	GGAGCGAAGTTGACTGCAA
		TCCTCCCGCCATCGTGTC
In-out PCR primers	RTEL1	GCATGACCCGCAAGCCC
		CGAGCGGGGGTACTGGAC
	TRF2	GTGCGATTAAGCCAGACATGAAGA
		AACCCTACGCAAGTAAGCCC
Primers related to Supplementary Figure 1C	Segment1	CACACAGGTCATCAACGAGC
		CACCTTCCCTCGCTTTCTGG
	Segment2	CTGAGCTACTGGTGCTTCAG
		GCCAGTACCGATTTCTGCG

Supplementary Table 2. Sequences of guide inserts within px330 plasmids and PCR primers.

Supplementary Movie 1. A representative 2D movie tracking wild-type Halo-RTEL1 (red) and mEos3.2-FKBP12^{F36V}-TRF2 (green) in live HeLa cell lines at the asynchronous state. The movie was captured with an exposure time of approximately 7.5 ms and an effective frame rate of about 134 fps. The total duration of the movie is 5 seconds.

Supplementary Movie 2. A representative 2D movie tracking wild-type Halo-RTEL1 (red) and mEos3.2-FKBP12F36V-TRF2 (green) in live HeLa cell lines during the S-phase. The movie was captured with an exposure time of approximately 7.5 ms and an effective frame rate of about 134 fps. The total duration of the movie is 5 seconds.

Supplementary Movie 3 A representative 2D movie tracking wild-type Halo-RTEL1 (red) and mEos3.2-FKBP12^{F36V}-TRF2 (green) in live HeLa cell lines at the G1/S boundary. The movie was captured with an exposure time of approximately 7.5 ms and an effective frame rate of about 134 fps. The total duration of the movie is 5 seconds.

Supplementary Movie 4. A representative 2D time-lapse movie tracking wild-type Halo-RTEL1 (red) and mEos3.2-FKBP12^{F36V}-TRF2 (green) in live HeLa cell lines during the S-phase. The movie was captured with an exposure time of 100 ms and an effective frame rate of 5 fps. The total duration of the movie is 90 seconds.

Supplementary Movie 5. A representative 2D movie tracking ATPase-dead (K48R) Halo-RTEL1 (red) and mEos3.2-FKBP12^{F36V}-TRF2 (green) in live HeLa cell lines at the asynchronous state. The movie was captured with an exposure time of approximately 7.5 ms and an effective frame rate of about 134 fps. The total duration of the movie is 5 seconds.

Supplementary Movie 6 A representative 2D time-lapse movie tracking wild-type Halo-RTEL1 (red) and mEos3.2-FKBP12^{F36V}-TRF2 (green) in live HeLa cell lines at the asynchronous state. The movie was captured with an exposure time of 100 ms and an effective frame rate of 5 fps. The total duration of the movie is 90 seconds.

Supplementary Movie 7 A representative 2D time-lapse movie tracking ATPase-dead (K48R) Halo-RTEL1 (red) and mEos3.2-FKBP12^{F36V}-TRF2 (green) in live HeLa cell lines at the

asynchronous state. The movie was captured with an exposure time of 100 ms and an effective frame rate of 5 fps. The total duration of the movie is 90 seconds.

Supplementary Movie 8. A representative 2D movie tracking wild-type Halo-RTEL1 (red) and mEos3.2-FKBP12^{F36V}-TRF2 (green) in live HeLa cell lines during the S-phase following dTAG^{V}-1 treatment. The movie was captured with an exposure time of approximately 7.5 ms and an effective frame rate of about 134 fps. The total duration of the movie is 5 seconds.

Supplementary Movie 9. A representative 2D time-lapse movie tracking wild-type Halo-RTEL1 (red) and mEos3.2-FKBP12^{F36V}-TRF2 (green) in live HeLa cell lines during the S-phase following dTAG^{V}-1 treatment. The movie was captured with an exposure time of 100 ms and an effective frame rate of 5 fps. The total duration of the movie is 90 seconds.

Supplementary Donor Vector Sequences

> WT FLAG-HaloTag-RTEL1_DonorVector

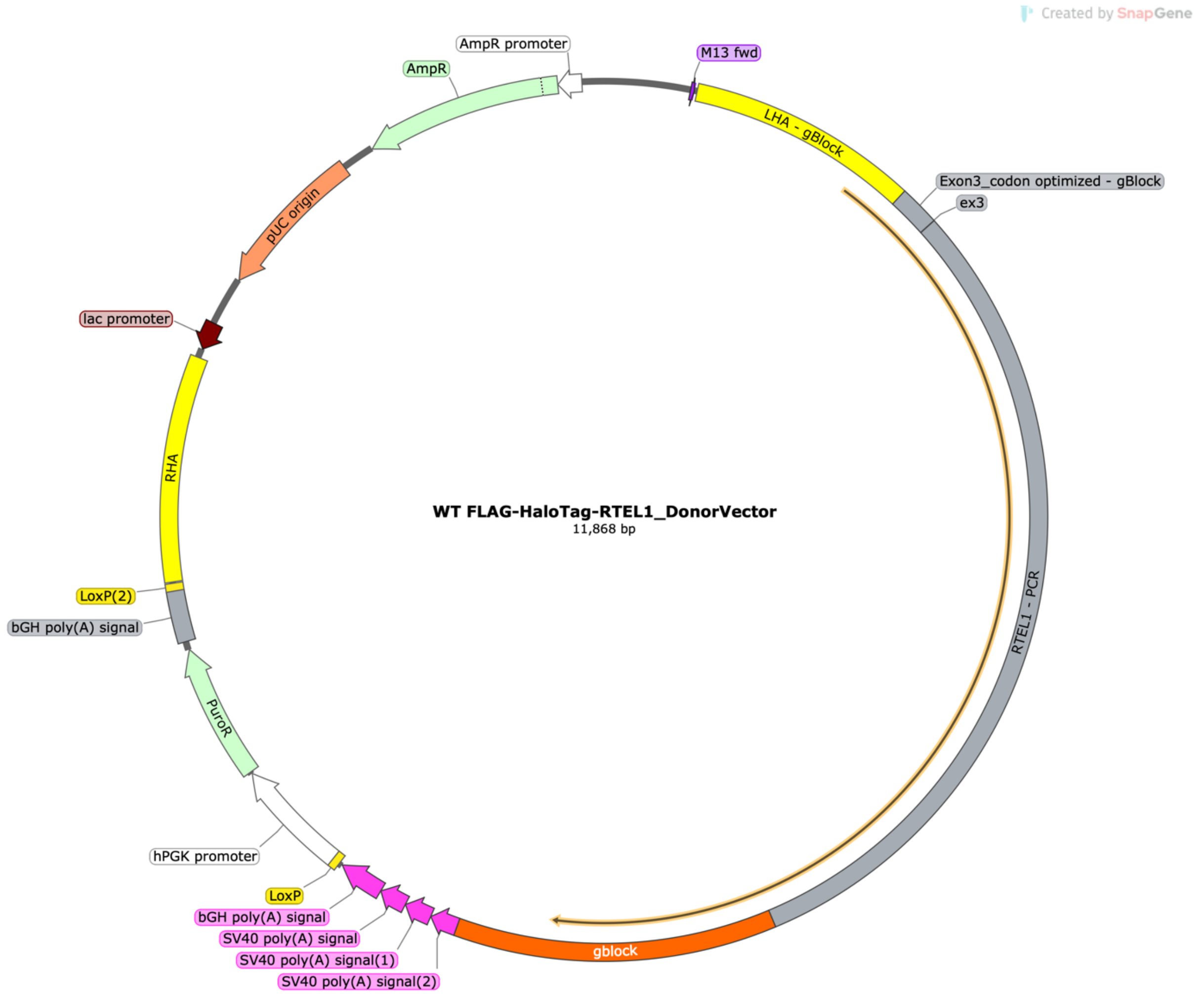

TCGCGCGTTTCGGTGATGACGGTGAAAACCTCTGACACATGCAGCTCCCGGAGACGGTCACAGCTTGTCT

GTAAGCGGATGCCGGGAGCAGACAAGCCCGTCAGGGCGCGTCAGCGGGTGTTGGCGGGTGTCGGGGCT

GGCTTAACTATGCGGCATCAGAGCAGATTGTACTGAGAGTGCACCATATGCGGTGTGAAATACCGCACAG

ATGCGTAAGGAGAAAATACCGCATCAGGCGCCATTCGCCATTCAGGCTGCGCAACTGTTGGGAAGGGCG

ATCGGTGCGGGCCTCTTCGCTATTACGCCAGCTGGCGAAAGGGGGATGTGCTGCAAGGCGATTAAGTTG

GGTAACGCCAGGGTTTTCCCAGTCACGACGTTGTAAAACGACGGCCAGTGAATTCGAGCTCACTATCACA

TGGAGAGAAACCTTGGGCAATACCCGGCTTTCCAGGGCAGAGGTCCCTGCGGCTTTCCGCAGTGCATCG

TGCCCCTGGTTTATCGAGACTGGAGAATGGCGATGACTTTTACCAAGCATACTGCCTGTAAACATATTGTTA

ACAAGGCACGTTCTGCACAGCTCTAGATCCCTTAAACCTTGATTCCATACAACACATGTTTCTGTGAGCTC

AAGGCTGGGGCAAAGTTACAGATTAACAGCATCTTAGGGCAAAGCAATTGTTCAGGGTACAGGTCAAAA
TGGAGTGTGTTATGTCTTCCCTTTCTACATAGACACAGTAACAGTCTGATCTCTCTTTTCCCTACAGTCCTTG
AGGGTGACAGACTTAGGAGTGCCTTGGGGGCCTCTCTGAGGAGCAGCTGATATTCACGGGTCAGGAGG
AAGCATTTCCATTAGAGGGGCAGCCGGTGGCCAGCCTCACTTGGAAGGTCTTTGAACCTCGGGGGTGCA
GGGAGGTGGCAGTGGTGCAGGTTGCCTTCTCCTGGGTTCCTTGAGGTGCCCTCTTGTACCCGGCTCACAC
CCTTCCCCTCCCCGAGTTTCCTGCTCAGGTTCCCGTCTGAGAGCTTGTATGTAGGACGTCAGATAGGACAG
CATAAATGTTTGGATCCAGAAACGCAGAACAGTTTCCTATTTTGAGACTTGACACCTAATTAGTCATCTTAC
TATTTAAGCTGAAAAATAGTGTCGTGTTTTGGGTAACGTTCTGCAAATCGTTTGCTAATGGCGGCTGAGTTG
CTTCACGCCCTTTAGGGCAAGAGTGGGACTTGCCTGTGGACTTCTCCGCGGTCCCACAGGGCTCTCGCCA
CCTGGCAGTGGCCTCTGCATCTGCAAAGAGCTGCCCGCTGGCTGCCGAAGCTTGTCTCAGGGCAGCTTGT
GTGGCCTCGCCTCTTCCTGGCTTCCCCGTAACCCTTGCTCCGAACTCCGTTCAGAAGGTGAATGGCATCCT
GGAGTCACCCACAGGAACTGGCAAAACACTGTGCCTGCTGTGCACAACCCTGGCTTGGAGGGAGCACC
TGAGAGACGGAATCAGCGCCAGAAAAATTGCAGAGAGGGCTCAGGGCGAGCTGTTCCCTGATAGAGCT
CTGTCAAGTTGGGGCAACGCCGCCGCCGCTGCCGGCGATCCTATCGCTTGCTACACGGACATCCCAAAG
ATTATTTACGCCTCCAGGACCCACTCGCAACTCACACAGGTCATCAACGAGCTTCGGAACACCTCCTACC
GGCCTAAGGTGTGTGTGCTGGGCTCCCGGGAGCAGCTGTGCATCCATCCTGAGGTGAAGAAACAAGAGA
GTAACCATCTACAGATCCACTTGTGCCGTAAGAAGGTGGCAAGTCGCTCCTGTCATTTCTACAACAACGTA
GAAGAAAAAAGCCTGGAGCAGGAGCTGGCCAGCCCCATCCTGGACATTGAGGACTTGGTCAAGAGCGG
AAGCAAGCACAGGGTGTGCCCTTACTACCTGTCCCGGAACCTGAAGCAGCAAGCCGACATCATATTCATG
CCGTACAATTACTTGTTGGATGCCAAGAGCCGCAGAGCACACAACATTGACCTGAAGGGGACAGTCGTG
ATCTTTGACGAAGCTCACAACGTGGAGAAGATGTGTGAAGAATCGGCATCCTTTGACCTGACTCCCCATG
ACCTGGCTTCAGGACTGGACGTCATAGACCAGGTGCTGGAGGAGCAGACCAAGGCAGCGCAGCAGGGT
GAGCCCCACCCGGAGTTCAGCGCGGACTCCCCCAGCCCAGGGCTGAACATGGAGCTGGAAGACATTGC
AAAGCTGAAGATGATCCTGCTGCGCCTGGAGGGGGCCATCGATGCTGTTGAGCTGCCTGGAGACGACAG
CGGTGTCACCAAGCCAGGGAGCTACATCTTTGAGCTGTTTGCTGAAGCCCAGATCACGTTTCAGACCAAG
GGCTGCATCCTGGACTCGCTGGACCAGATCATCCAGCACCTGGCAGGACGTGCTGGAGTGTTCACCAAC
ACGGCCGGACTGCAGAAGCTGGCGGACATTATCCAGATTGTGTTCAGTGTGGACCCCTCCGAGGGCAGC
CCTGGTTCCCCAGCAGGGCTGGGGGCCTTACAGTCCTATAAGGTGCACATCCATCCTGATGCTGGTCACC
GGAGGACGGCTCAGCGGTCTGATGCCTGGAGCACCACTGCAGCCAGAAAGCGAGGGAAGGTGCTGAG

CTACTGGTGCTTCAGTCCCGGCCACAGCATGCACGAGCTGGTCCGCCAGGGCGTCCGCTCCCTCATCCTT
ACCAGCGGCACGCTGGCCCCGGTGTCCTCCTTTGCTCTGGAGATGCAGATCCCTTTCCCAGTCTGCCTGG
AGAACCCACACATCATCGACAAGCACCAGATCTGGGTGGGGGTCGTCCCCAGAGGCCCCGATGGAGCCC
AGTTGAGCTCCGCGTTTGACAGACGGTTTTCCGAGGAGTGCTTATCCTCCCTGGGGAAGGCTCTGGGCAA
CATCGCCCGCGTGGTGCCCTATGGGCTCCTGATCTTCTTCCCTTCCTATCCTGTCATGGAGAAGAGCCTGGA
GTTCTGGCGGGCCCGCGACTTGGCCAGGAAGATGGAGGCGCTGAAGCCGCTGTTTGTGGAGCCCAGGA
GCAAAGGCAGCTTCTCCGAGACCATCAGTGCTTACTATGCAAGGGTTGCCGCCCCTGGGTCCACCGGCG
CCACCTTCCTGGCGGTCTGCCGGGGCAAGGCCAGCGAGGGGCTGGACTTCTCAGACACGAATGGCCGT
GGTGTGATTGTCACGGGCCTCCCGTACCCCCCACGCATGGACCCCCGGGTTGTCCTCAAGATGCAGTTCCT
GGATGAGATGAAGGGCCAGGGTGGGGCTGGGGGCCAGTTCCTCTCTGGGCAGGAGTGGTACCGGCAGC
AGGCGTCCAGGGCTGTGAACCAGGCCATCGGGCGAGTGATCCGGCACCGCCAGGACTACGGAGCTGTC
TTCCTCTGTGACCACAGGTTCGCCTTTGCCGACGCAAGAGCCCAACTGCCCTCCTGGGTGCGTCCCCACG
TCAGGGTGTATGACAACTTTGGCCATGTCATCCGAGACGTGGCCCAGTTCTTCCGTGTTGCCGAGCGAACT
ATGCCAGCGCCGGCCCCCCGGGCTACAGCACCCAGTGTGCGTGGAGAAGATGCTGTCAGCGAGGCCAA
GTCGCCTGGCCCCTTCTTCTCCACCAGGAAAGCTAAGAGTCTGGACCTGCATGTCCCCAGCCTGAAGCAG
AGGTCCTCAGGGTCACCAGCTGCCGGGGACCCCGAGAGTAGCCTGTGTGTGGAGTATGAGCAGGAGCCA
GTTCCTGCCCGGCAGAGGCCCAGGGGGCTGCTGGCCGCCCTGGAGCACAGCGAACAGCGGGCGGGGA
GCCCTGGCGAGGAGCAGGCCCACAGCTGCTCCACCCTGTCCCTCCTGTCTGAGAAGAGGCCGGCAGAA
GAACCGCGAGGAGGGAGGAAGAAGATCCGGCTGGTCAGCCACCCGGAGGAGCCCGTGGCTGGTGCAC
AGACGGACAGGGCCAAGCTCTTCATGGTGGCCGTGAAGCAGGAGTTGAGCCAAGCCAACTTTGCCACC
TTCACCCAGGCCCTGCAGGACTACAAGGGTTCCGATGACTTCGCCGCCCTGGCCGCCTGTCTCGGCCCCC
TCTTTGCTGAGGACCCCAAGAAGCACAACCTGCTCCAAGGCTTCTACCAGTTTGTGCGGCCCCACCATAA
GCAGCAGTTTGAGGAGGTCTGTATCCAGCTGACAGGACGAGGCTGTGGCTATCGGCCTGAGCACAGCAT
TCCCCGAAGGCAGCGGGCACAGCCGGTCCTGGACCCCACTGGAAGAACGGCGCCGGATCCCAAGCTGA
CCGTGTCCACGGCTGCAGCCCAGCAGCTGGACCCCCAAGAGCACCTGAACCAGGGCAGGCCCCACCTG
TCGCCCAGGCCACCCCCAACAGGAGACCCTGGCAGCCAACCACAGTGGGGGTCTGGAGTGCCCAGAGC
AGGGAAGCAGGGCCAGCACGCCGTGAGCGCCTACCTGGCTGATGCCCGCAGGGCCCTGGGGTCCGCGG
GCTGTAGCCAACTCTTGGCAGCGCTGACAGCCTATAAGCAAGACGACGACCTCGACAAGGTGCTGGCTG
TGTTGGCCGCCCTGACCACTGCAAAGCCAGAGGACTTCCCCCTGCTGCACAGGTTCAGCATGTTTGTGCG

TCCACACCACAAGCAGCGCTTCTCACAGACGTGCACAGACCTGACCGGCCGGCCCTACCCGGGCATGGA
GCCACCGGGACCCCAGGAGGAGAGGCTTGCCGTGCCTCCTGTGCTTACCCACAGGGCTCCCCAACCAG
GCCCCTCACGGTCCGAGAAGACCGGGAAGACCCAGAGCAAGATCTCGTCCTTCCTTAGACAGAGGCCAG
CAGGGACTGTGGGGGCGGGCGGTGAGGATGCAGGTCCCAGCCAGTCCTCAGGACCTCCCCACGGGCCT
GCAGCATCTGAGTGGGGTGAGCCTCATGGGAGAGACATCGCTGGGCAGCAGGCCACGGGAGCTCCGGG
CGGGCCCCTCTCAGCAGGCTGTGTGTGCCAGGGCTGTGGGGCAGAGGACGTGGTGCCCTTCCAGTGCCC
TGCCTGTGACTTCCAGCGCTGCCAAGCCTGCTGGCAACGGCACCTTCAGGCCTCTAGGATGTGCCCAGCC
TGCCACACCGCCTCCAGGAAGCAGAGCGTCATGCAGGTCTTCTGGCCAGAGCCCCAGGGTACCGAGCCA
ACCACTGAGGATCTGTACTTTCAGAGCGATAACGCGATCGCAGAAATCGGTACTGGCTTTCCATTCGACCC
CCATTATGTGGAAGTCCTGGGCGAGCGCATGCACTACGTCGATGTTGGTCCGCGCGATGGCACCCCTGTG
CTGTTCCTGCACGGTAACCCGACCTCCTCCTACGTGTGGCGCAACATCATCCCGCATGTTGCACCGACCCA
TCGCTGCATTGCTCCAGACCTGATCGGTATGGGCAAATCCGACAAACCAGACCTGGGTTATTTCTTCGACG
ACCACGTCCGCTTCATGGATGCCTTCATCGAAGCCCTGGGTCTGGAAGAGGTCGTCCTGGTCATTCACGAC
TGGGGCTCCGCTCTGGGTTTCCACTGGGCCAAGCGCAATCCAGAGCGCGTCAAAGGTATTGCATTTATGG
AGTTCATCCGCCCTATCCCGACCTGGGACGAATGGCCAGAATTTGCCCGCGAGACCTTCCAGGCCTTCCG
CACCACCGACGTCGGCCGCAAGCTGATCATCGATCAGAACGTTTTTATCGAGGGTACGCTGCCGATGGGT
GTCGTCCGCCCGCTGACTGAAGTCGAGATGGACCATTACCGCGAGCCGTTCCTGAATCCTGTTGACCGCG
AGCCACTGTGGCGCTTCCCAAACGAGCTGCCAATCGCCGGTGAGCCAGCGAACATCGTCGCGCTGGTCG
AAGAATACATGGACTGGCTGCACCAGTCCCCTGTCCCGAAGCTGCTGTTCTGGGGCACCCCAGGCGTTCT
GATCCCACCGGCCGAAGCCGCTCGCCTGGCCAAAAGCCTGCCTAACTGCAAGGCTGTGGACATCGGCCC
GGGTCTGAATCTGCTGCAAGAAGACAACCCGGACCTGATCGGCAGCGAGATCGCGCGCTGGCTGTCAAC
GCTCGAGATTTCCGGCGACTACAAAGACCATGACGGTGATTATAAAGATCATGACATCGATTACAAGGAT
GACGATGACAAGTGAGTGCCCACGGAGGCCCCCAGCACACCCAACGTGGCTTGATCACCTGCCTGTCCA
GCTCTGGTGGGCCAAGAACCCACCCAACAGAATAGGCCAGCCCATGCCAGCCGGCTTGGCCCGCTGCA
GGCCTCAGGCAGGCGGGGCCCATGGTTGGTCCCTGCGGTGGGACCGGATCTGGGCCTGCCTCTGAGAA
GCCCTGAGCTACCTTGGGGTCTGGGGTGGGTTTCTGGGAAAGTGCTTCCCCAGAACTTCCCTGGCTCCTG
GCCTGTGAGTGGTGCCACAGGGGCACCCCAGCTGAGCCCCTCACCGGGAAGGAGGAGACCCCCGTGGG
CACGTGTCCACTTTTAATCAGGGGACAGGGCTCTCTAATAAAGCTGCTGGCAGTGCCCAGGTACCAACTT
GTTTATTGCAGCTTATAATGGTTACAAATAAAGCAATAGCATCACAAATTTCACAAATAAAGCATTTTTTTC

ACTGCATTCTAGTTGTGGTTTGTCCAAACTCATCAATGTATCTTAAACTTGTTTATTGCAGCTTATAATGGTTA
CAAATAAAGCAATAGCATCACAAATTTCACAAATAAAGCATTTTTTTCACTGCATTCTAGTTGTGGTTTGTC
CAAACTCATCAATGTATCTTAAACTTGTTTATTGCAGCTTATAATGGTTACAAATAAAGCAATAGCATCACA
AATTTCACAAATAAAGCATTTTTTTCACTGCATTCTAGTTGTGGTTTGTCCAAACTCATCAATGTATCTTAGA
CTGTGCCTTCTAGTTGCCAGCCATCTGTTGTTTGCCCCTCCCCCGTGCCTTCCTTGACCCTGGAAGGTGCCA
CTCCCACTGTCCTTTCCTAATAAAATGAGGAAATTGCATCGCATTGTCTGAGTAGGTGTCATTCTATTCTGG
GGGGTGGGGTGGGGCAGGACAGCAAGGGGGAGGATTGGGAAGAGAATAGCAGGCATGCGGCCGCGA
TATCATAACTTCGTATAATGTATGCTATACGAAGTTATGGGGTTGGGGTTGCGCCTTTTCCAAGGCAGCCCT
GGGTTTGCGCAGGGACGCGGCTGCTCTGGGCGTGGTTCCGGGAAACGCAGCGGCGCCGACCCTGGGTC
TCGCACATTCTTCACGTCCGTTCGCAGCGTCACCCGGATCTTCGCCGCTACCCTTGTGGGCCCCCCGGCGA
CGCTTCCTGCTCCGCCCCTAAGTCGGGAAGGTTCCTTGCGGTTCGCGGCGTGCCGGACGTGACAAACGG
AAGCCGCACGTCTCACTAGTACCCTCGCAGACGGACAGCGCCAGGGAGCAATGGCAGCGCGCCGACCG
CGATGGGCTGTGGCCAATAGCGGCTGCTCAGCAGGGCGCGCCGAGAGCAGCGGCCGGGAAGGGGCGG
TGCGGGAGGCGGGGTGTGGGGCGGTAGTGTGGGCCCTGTTCCTGCCCGCGCGGTGTTCCGCATTCTGCA
AGCCTCCGGAGCGCACGTCGGCAGTCGGCTCCCTCGTTGACCGAATCACCGACCTCTCTCCCCAGCAATT
CACCATGACCGAGTACAAGCCCACGGTGCGCCTCGCCACCCGCGACGACGTCCCCAGGGCCGTACGCAC
CCTCGCCGCCGCGTTCGCCGACTACCCCGCCACGCGCCACACCGTCGATCCGGACCGCCACATCGAGCG
GGTCACCGAGCTGCAAGAACTCTTCCTCACGCGCGTCGGGCTCGACATCGGCAAGGTGTGGGTCGCGGA
CGACGGCGCCGCGGTGGCGGTCTGGACCACGCCGGAGAGCGTCGAAGCGGGGGCGGTGTTCGCCGAG
ATCGGCCCGCGCATGGCCGAGTTGAGCGGTTCCCGGCTGGCCGCGCAGCAACAGATGGAAGGCCTCCT
GGCGCCGCACCGGCCCAAGGAGCCCGCGTGGTTCCTGGCCACCGTCGGCGTCTCGCCCGACCACCAGG
GCAAGGGTCTGGGCAGCGCCGTCGTGCTCCCCGGAGTGGAGGCGGCCGAGCGCGCCGGGGTGCCCGC
CTTCCTGGAGACCTCCGCGCCCCGCAACCTCCCCTTCTACGAGCGGCTCGGCTTCACCGTCACCGCCGAC
GTCGAGGTGCCCGAAGGACCGCGCACCTGGTGCATGACCCGCAAGCCCGGTGCCTGACTCGAGTCTAGA
CCAATTGGTTTAAACCCTGCAGGCTGTGCCTTCTAGTTGCCAGCCATCTGTTGTTTGCCCCTCCCCCGTGCC
TTCCTTGACCCTGGAAGGTGCCACTCCCACTGTCCTTTCCTAATAAAATGAGGAAATTGCATCGCATTGTCT
GAGTAGGTGTCATTCTATTCTGGGGGGTGGGGTGGGGCAGGACAGCAAGGGGGAGGATTGGGAAGACA
ATAGCAGGCATGCTGGGGATGCGGTGGGCTCTATGGATAACTTCGTATAATGTATGCTATACGAAGTTATCC
TAGGTACAGGGAAGACGCTGTGCCTGCTGTGCACCACGCTGGCCTGGCGAGAACACCTCCGAGACGGC

ATCTCTGCCCGCAAGATTGCCGAGAGGGCGCAAGGAGAGCTTTTCCCGGATCGGGCCTTGTCATCCTGG
GGCAACGCTGCTGCTGCTGCTGGAGACCCCATAGGTGACCCTAGTTCCCAGGCCTCTCCTGGCCTCCTGT
GGGGATGGTTGGCAAGGGATGGCGCTGAGGGTGGGGTGGGCCCATGGGGACTCCTGCCGTCTCTCAAG
CAGAACTCAAGGAGAATTTTTTAGCTGCTGTATAATTTCTCGCCATCGTGGGTGTAAACCTAGGGTTGGGC
TTTTTTGCTGAATTAGGGCACGGCAGATGCCCACTTCACCCATTTTTGATAAACCAGTATCTGGGGTGTCA
GATTCTTGGCTGTCTGCAGGGCCGAGTTAGCCGAATGCCACCTGCCTTTGATACGTGAGAACGTTGTCTGA
GAACCGTGACTTCTGTGCTTGCTTGTGTCTGGTCAGCTTGCTACACGGACATCCCAAAGATTATTTACGCCT
CCAGGACCCACTCGCAACTCACACAGGTCATCAACGAGCTTCGGAACACCTCCTACCGGTGGGTCAGAC
GAGTTTACACCTGTCTCGGGGTCCTCAAGAGAACCAGCTTGGCATGGTGCTGAGTCCACAGCCCCATGCT
GTGCTGTGGTGGAGGGTGGTGGTCTTTCTAGACGCTCCCCCGAAGTGTGCAGAGCGCTGGTGCCCAGGG
GTGGGGTGCGGCCTGGGCTGCCTCCAATGCCCATTACTTGTGAGGAAGCAGCTTTGCATCTGTGTGCTGA
CCTTGGGCGGGCGTCCTGAGCTCCTCGCAGGTGCTGTTGTAGCAGCTGTGCAGTAGGTCAGGGCTGGCC
CCCAGTGCAGCTTTGCACATGAAGTAGGAGGAGGCCCTGCTGCTTGTCAGAGCCCAGCAGAGTCTTGGT
GTTCTGTCGGGTTCCTGGTCGACCTGCAGGCATGCAAGCTTGGCGTAATCATGGTCATAGCTGTTTCCTGT
GTGAAATTGTTATCCGCTCACAATTCCACACAACATACGAGCCGGAAGCATAAAGTGTAAAGCCTGGGGT
GCCTAATGAGTGAGCTAACTCACATTAATTGCGTTGCGCTCACTGCCCGCTTTCCAGTCGGGAAACCTGTC
GTGCCAGCTGCATTAATGAATCGGCCAACGCGCGGGGAGAGGCGGTTTGCGTATTGGGCGCTCTTCCGC
TTCCTCGCTCACTGACTCGCTGCGCTCGGTCGTTCGGCTGCGGCGAGCGGTATCAGCTCACTCAAAGGCG
GTAATACGGTTATCCACAGAATCAGGGGATAACGCAGGAAAGAACATGTGAGCAAAAGGCCAGCAAAA
GGCCAGGAACCGTAAAAAGGCCGCGTTGCTGGCGTTTTTCCATAGGCTCCGCCCCCCTGACGAGCATCA
CAAAAATCGACGCTCAAGTCAGAGGTGGCGAAACCCGACAGGACTATAAAGATACCAGGCGTTTCCCCC
TGGAAGCTCCCTCGTGCGCTCTCCTGTTCCGACCCTGCCGCTTACCGGATACCTGTCCGCCTTTCTCCCTTC
GGGAAGCGTGGCGCTTTCTCATAGCTCACGCTGTAGGTATCTCAGTTCGGTGTAGGTCGTTCGCTCCAAGC
TGGGCTGTGTGCACGAACCCCCCGTTCAGCCCGACCGCTGCGCCTTATCCGGTAACTATCGTCTTGAGTCC
AACCCGGTAAGACACGACTTATCGCCACTGGCAGCAGCCACTGGTAACAGGATTAGCAGAGCGAGGTAT
GTAGGCGGTGCTACAGAGTTCTTGAAGTGGTGGCCTAACTACGGCTACACTAGAAGGACAGTATTTGGTA
TCTGCGCTCTGCTGAAGCCAGTTACCTTCGGAAAAAGAGTTGGTAGCTCTTGATCCGGCAAACAAACCAC
CGCTGGTAGCGGTGGTTTTTTTGTTTGCAAGCAGCAGATTACGCGCAGAAAAAAAGGATCTCAAGAAGA
TCCTTTGATCTTTTCTACGGGGTCTGACGCTCAGTGGAACGAAAACTCACGTTAAGGGATTTTGGTCATGA

GATTATCAAAAAGGATCTTCACCTAGATCCTTTTAAATTAAAAATGAAGTTTTAAATCAATCTAAAGTATATA
TGAGTAAACTTGGTCTGACAGTTACCAATGCTTAATCAGTGAGGCACCTATCTCAGCGATCTGTCTATTTCG
TTCATCCATAGTTGCCTGACTCCCCGTCGTGTAGATAACTACGATACGGGAGGGCTTACCATCTGGCCCCA
GTGCTGCAATGATACCGCGAGACCCACGCTCACCGGCTCCAGATTTATCAGCAATAAACCAGCCAGCCGG
AAGGGCCGAGCGCAGAAGTGGTCCTGCAACTTTATCCGCCTCCATCCAGTCTATTAATTGTTGCCGGGAA
GCTAGAGTAAGTAGTTCGCCAGTTAATAGTTTGCGCAACGTTGTTGCCATTGCTACAGGCATCGTGGTGTC
ACGCTCGTCGTTTGGTATGGCTTCATTCAGCTCCGGTTCCCAACGATCAAGGCGAGTTACATGATCCCCCA
TGTTGTGCAAAAAAGCGGTTAGCTCCTTCGGTCCTCCGATCGTTGTCAGAAGTAAGTTGGCCGCAGTGTTA
TCACTCATGGTTATGGCAGCACTGCATAATTCTCTTACTGTCATGCCATCCGTAAGATGCTTTTCTGTGACTG
GTGAGTACTCAACCAAGTCATTCTGAGAATAGTGTATGCGGCGACCGAGTTGCTCTTGCCCGGCGTCAATA
CGGGATAATACCGCGCCACATAGCAGAACTTTAAAAGTGCTCATCATTGGAAAACGTTCTTCGGGGCGAA
AACTCTCAAGGATCTTACCGCTGTTGAGATCCAGTTCGATGTAACCCACTCGTGCACCCAACTGATCTTCA
GCATCTTTTACTTTCACCAGCGTTTCTGGGTGAGCAAAAACAGGAAGGCAAAATGCCGCAAAAAAGGGA
ATAAGGGCGACACGGAAATGTTGAATACTCATACTCTTCCTTTTTCAATATTATTGAAGCATTTATCAGGGT
TATTGTCTCATGAGCGGATACATATTTGAATGTATTTAGAAAAATAAACAAATAGGGGTTCCGCGCACATTT
CCCCGAAAAGTGCCACCTGACGTCTAAGAAACCATTATTATCATGACATTAACCTATAAAAATAGGCGTATC
ACGAGGCCCTTTCGTC

> K48R FLAG-HaloTag-RTEL1_DonorVector (K48R mutation is labeled in red)

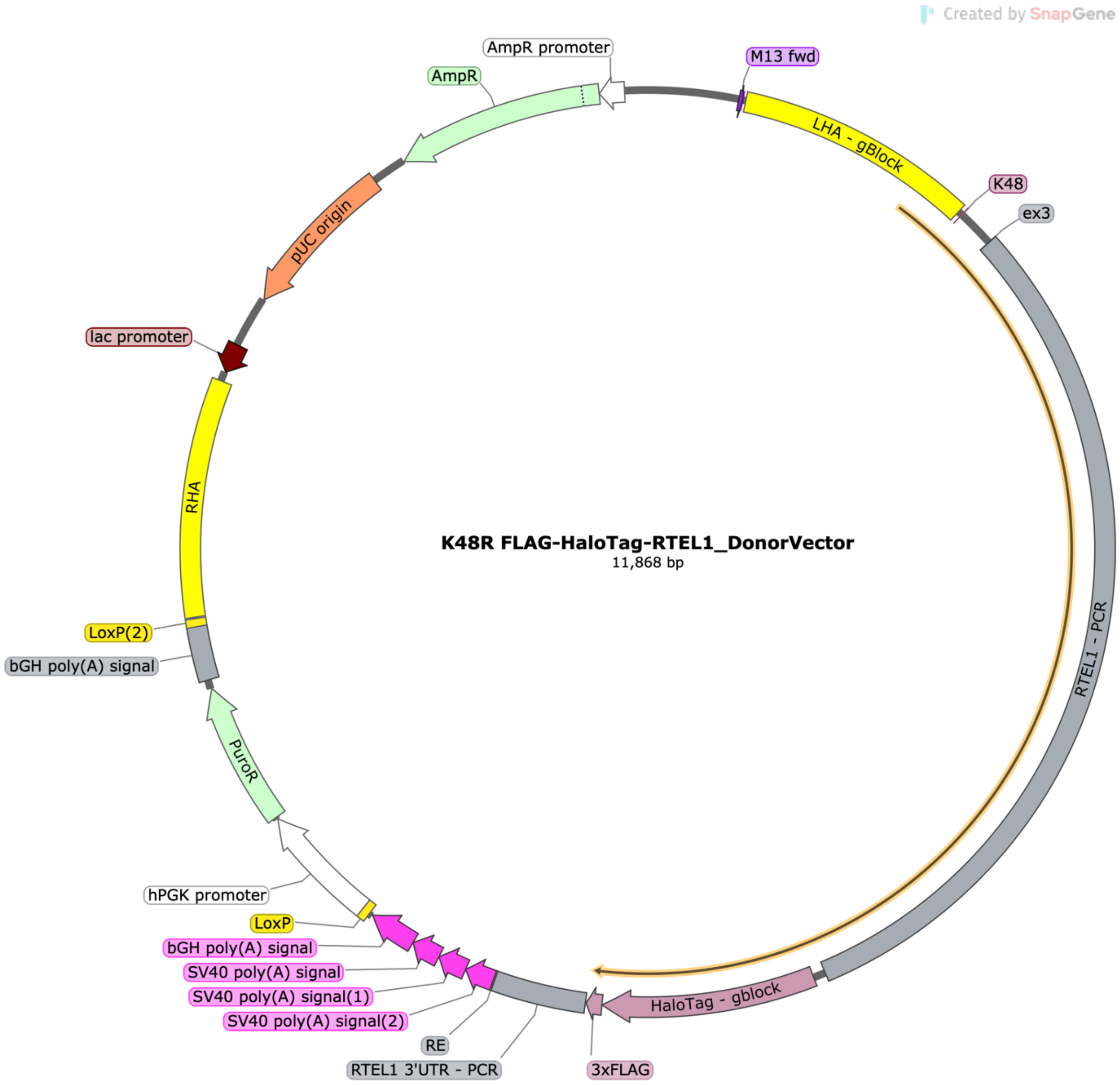

TCGCGCGTTTCGGTGATGACGGTGAAAACCTCTGACACATGCAGCTCCCGGAGACGGTCACAGCTTGTCT

GTAAGCGGATGCCGGGAGCAGACAAGCCCGTCAGGGCGCGTCAGCGGGTGTTGGCGGGTGTCGGGGCT

GGCTTAACTATGCGGCATCAGAGCAGATTGTACTGAGAGTGCACCATATGCGGTGTGAAATACCGCACAG

ATGCGTAAGGAGAAAATACCGCATCAGGCGCCATTCGCCATTCAGGCTGCGCAACTGTTGGGAAGGGCG

ATCGGTGCGGGCCTCTTCGCTATTACGCCAGCTGGCGAAAGGGGGATGTGCTGCAAGGCGATTAAGTTG

GGTAACGCCAGGGTTTTCCCAGTCACGACGTTGTAAAACGACGGCCAGTGAATTCGAGCTCACTATCACA

TGGAGAGAAACCTTGGGCAATACCCGGCTTTCCAGGGCAGAGGTCCCTGCGGCTTTCCGCAGTGCATCG

TGCCCCTGGTTTATCGAGACTGGAGAATGGCGATGACTTTTACCAAGCATACTGCCTGTAAACATATTGTTA

ACAAGGCACGTTCTGCACAGCTCTAGATCCCTTAAACCTTGATTCCATACAACACATGTTTCTGTGAGCTC

AAGGCTGGGGCAAAGTTACAGATTAACAGCATCTTAGGGCAAAGCAATTGTTCAGGGTACAGGTCAAAA

TGGAGTGTGTTATGTCTTCCCTTTCTACATAGACACAGTAACAGTCTGATCTCTCTTTTCCCTACAGTCCTTG

AGGGTGACAGACTTAGGAGTGCCTTGGGGGCCTCTCTGAGGAGCAGCTGATATTCACGGGTCAGGAGG

AAGCATTTCCATTAGAGGGGCAGCCGGTGGCCAGCCTCACTTGGAAGGTCTTTGAACCTCGGGGGTGCA

GGGAGGTGGCAGTGGTGCAGGTTGCCTTCTCCTGGGTTCCTTGAGGTGCCCTCTTGTACCCGGCTCACAC

CCTTCCCCTCCCCGAGTTTCCTGCTCAGGTTCCCGTCTGAGAGCTTGTATGTAGGACGTCAGATAGGACAG

CATAAATGTTTGGATCCAGAAACGCAGAACAGTTTCCTATTTTGAGACTTGACACCTAATTAGTCATCTTAC

TATTTAAGCTGAAAAATAGTGTCGTGTTTTGGGTAACGTTCTGCAAATCGTTTGCTAATGGCGGCTGAGTTG

CTTCACGCCCTTTAGGGCAAGAGTGGGACTTGCCTGTGGACTTCTCCGCGGTCCCACAGGGCTCTCGCCA

CCTGGCAGTGGCCTCTGCATCTGCAAAGAGCTGCCCGCTGGCTGCCGAAGCTTGTCTCAGGGCAGCTTGT

GTGGCCTCGCCTCTTCCTGGCTTCCCCGTAACCCTTGCTCCGAACTCCGTTCAGAAGGTGAATGGCATCCT

GGAGTCACCCACAGGAACTGGCAGGACACTGTGCCTGCTGTGCACAACCCTGGCTTGGAGGGAGCACC

TGAGAGACGGAATCAGCGCCAGAAAAATTGCAGAGAGGGCTCAGGGCGAGCTGTTCCCTGATAGAGCT

CTGTCAAGTTGGGGCAACGCCGCCGCCGCTGCCGGCGATCCTATCGCTTGCTACACGGACATCCCAAAG

ATTATTTACGCCTCCAGGACCCACTCGCAACTCACACAGGTCATCAACGAGCTTCGGAACACCTCCTACC

GGCCTAAGGTGTGTGTGCTGGGCTCCCGGGAGCAGCTGTGCATCCATCCTGAGGTGAAGAAACAAGAGA

GTAACCATCTACAGATCCACTTGTGCCGTAAGAAGGTGGCAAGTCGCTCCTGTCATTTCTACAACAACGTA

GAAGAAAAAAGCCTGGAGCAGGAGCTGGCCAGCCCCATCCTGGACATTGAGGACTTGGTCAAGAGCGG

AAGCAAGCACAGGGTGTGCCCTTACTACCTGTCCCGGAACCTGAAGCAGCAAGCCGACATCATATTCATG

CCGTACAATTACTTGTTGGATGCCAAGAGCCGCAGAGCACACAACATTGACCTGAAGGGGACAGTCGTG

ATCTTTGACGAAGCTCACAACGTGGAGAAGATGTGTGAAGAATCGGCATCCTTTGACCTGACTCCCCATG

ACCTGGCTTCAGGACTGGACGTCATAGACCAGGTGCTGGAGGAGCAGACCAAGGCAGCGCAGCAGGGT

GAGCCCCACCCGGAGTTCAGCGCGGACTCCCCCAGCCCAGGGCTGAACATGGAGCTGGAAGACATTGC

AAAGCTGAAGATGATCCTGCTGCGCCTGGAGGGGGCCATCGATGCTGTTGAGCTGCCTGGAGACGACAG

CGGTGTCACCAAGCCAGGGAGCTACATCTTTGAGCTGTTTGCTGAAGCCCAGATCACGTTTCAGACCAAG

GGCTGCATCCTGGACTCGCTGGACCAGATCATCCAGCACCTGGCAGGACGTGCTGGAGTGTTCACCAAC

ACGGCCGGACTGCAGAAGCTGGCGGACATTATCCAGATTGTGTTCAGTGTGGACCCCTCCGAGGGCAGC

CCTGGTTCCCCAGCAGGGCTGGGGGCCTTACAGTCCTATAAGGTGCACATCCATCCTGATGCTGGTCACC
GGAGGACGGCTCAGCGGTCTGATGCCTGGAGCACCACTGCAGCCAGAAAGCGAGGGAAGGTGCTGAG
CTACTGGTGCTTCAGTCCCGGCCACAGCATGCACGAGCTGGTCCGCCAGGGCGTCCGCTCCCTCATCCTT
ACCAGCGGCACGCTGGCCCCGGTGTCCTCCTTTGCTCTGGAGATGCAGATCCCTTTCCCAGTCTGCCTGG
AGAACCCACACATCATCGACAAGCACCAGATCTGGGTGGGGGTCGTCCCCAGAGGCCCCGATGGAGCCC
AGTTGAGCTCCGCGTTTGACAGACGGTTTTCCGAGGAGTGCTTATCCTCCCTGGGGAAGGCTCTGGGCAA
CATCGCCCGCGTGGTGCCCTATGGGCTCCTGATCTTCTTCCCTTCCTATCCTGTCATGGAGAAGAGCCTGGA
GTTCTGGCGGGCCCGCGACTTGGCCAGGAAGATGGAGGCGCTGAAGCCGCTGTTTGTGGAGCCCAGGA
GCAAAGGCAGCTTCTCCGAGACCATCAGTGCTTACTATGCAAGGGTTGCCGCCCCTGGGTCCACCGGCG
CCACCTTCCTGGCGGTCTGCCGGGGCAAGGCCAGCGAGGGGCTGGACTTCTCAGACACGAATGGCCGT
GGTGTGATTGTCACGGGCCTCCCGTACCCCCCACGCATGGACCCCCGGGTTGTCCTCAAGATGCAGTTCCT
GGATGAGATGAAGGGCCAGGGTGGGGCTGGGGGCCAGTTCCTCTCTGGGCAGGAGTGGTACCGGCAGC
AGGCGTCCAGGGCTGTGAACCAGGCCATCGGGCGAGTGATCCGGCACCGCCAGGACTACGGAGCTGTC
TTCCTCTGTGACCACAGGTTCGCCTTTGCCGACGCAAGAGCCCAACTGCCCTCCTGGGTGCGTCCCCACG
TCAGGGTGTATGACAACTTTGGCCATGTCATCCGAGACGTGGCCCAGTTCTTCCGTGTTGCCGAGCGAACT
ATGCCAGCGCCGGCCCCCCGGGCTACAGCACCCAGTGTGCGTGGAGAAGATGCTGTCAGCGAGGCCAA
GTCGCCTGGCCCCTTCTTCTCCACCAGGAAAGCTAAGAGTCTGGACCTGCATGTCCCCAGCCTGAAGCAG
AGGTCCTCAGGGTCACCAGCTGCCGGGGACCCCGAGAGTAGCCTGTGTGTGGAGTATGAGCAGGAGCCA
GTTCCTGCCCGGCAGAGGCCCAGGGGGCTGCTGGCCGCCCTGGAGCACAGCGAACAGCGGGCGGGGA
GCCCTGGCGAGGAGCAGGCCCACAGCTGCTCCACCCTGTCCCTCCTGTCTGAGAAGAGGCCGGCAGAA
GAACCGCGAGGAGGGAGGAAGAAGATCCGGCTGGTCAGCCACCCGGAGGAGCCCGTGGCTGGTGCAC
AGACGGACAGGGCCAAGCTCTTCATGGTGGCCGTGAAGCAGGAGTTGAGCCAAGCCAACTTTGCCACC
TTCACCCAGGCCCTGCAGGACTACAAGGGTTCCGATGACTTCGCCGCCCTGGCCGCCTGTCTCGGCCCCC
TCTTTGCTGAGGACCCCAAGAAGCACAACCTGCTCCAAGGCTTCTACCAGTTTGTGCGGCCCCACCATAA
GCAGCAGTTTGAGGAGGTCTGTATCCAGCTGACAGGACGAGGCTGTGGCTATCGGCCTGAGCACAGCAT
TCCCCGAAGGCAGCGGGCACAGCCGGTCCTGGACCCCACTGGAAGAACGGCGCCGGATCCCAAGCTGA
CCGTGTCCACGGCTGCAGCCCAGCAGCTGGACCCCCAAGAGCACCTGAACCAGGGCAGGCCCCACCTG
TCGCCCAGGCCACCCCCAACAGGAGACCCTGGCAGCCAACCACAGTGGGGGTCTGGAGTGCCCAGAGC
AGGGAAGCAGGGCCAGCACGCCGTGAGCGCCTACCTGGCTGATGCCCGCAGGGCCCTGGGGTCCGCGG

GCTGTAGCCAACTCTTGGCAGCGCTGACAGCCTATAAGCAAGACGACGACCTCGACAAGGTGCTGGCTG
TGTTGGCCGCCCTGACCACTGCAAAGCCAGAGGACTTCCCCCTGCTGCACAGGTTCAGCATGTTTGTGCG
TCCACACCACAAGCAGCGCTTCTCACAGACGTGCACAGACCTGACCGGCCGGCCCTACCCGGGCATGGA
GCCACCGGGACCCCAGGAGGAGAGGCTTGCCGTGCCTCCTGTGCTTACCCACAGGGCTCCCCAACCAG
GCCCCTCACGGTCCGAGAAGACCGGGAAGACCCAGAGCAAGATCTCGTCCTTCCTTAGACAGAGGCCAG
CAGGGACTGTGGGGGCGGGCGGTGAGGATGCAGGTCCCAGCCAGTCCTCAGGACCTCCCCACGGGCCT
GCAGCATCTGAGTGGGGTGAGCCTCATGGGAGAGACATCGCTGGGCAGCAGGCCACGGGAGCTCCGGG
CGGGCCCCTCTCAGCAGGCTGTGTGTGCCAGGGCTGTGGGGCAGAGGACGTGGTGCCCTTCCAGTGCCC
TGCCTGTGACTTCCAGCGCTGCCAAGCCTGCTGGCAACGGCACCTTCAGGCCTCTAGGATGTGCCCAGCC
TGCCACACCGCCTCCAGGAAGCAGAGCGTCATGCAGGTCTTCTGGCCAGAGCCCCAGGGTACCGAGCCA
ACCACTGAGGATCTGTACTTTCAGAGCGATAACGCGATCGCAGAAATCGGTACTGGCTTTCCATTCGACCC
CCATTATGTGGAAGTCCTGGGCGAGCGCATGCACTACGTCGATGTTGGTCCGCGCGATGGCACCCCTGTG
CTGTTCCTGCACGGTAACCCGACCTCCTCCTACGTGTGGCGCAACATCATCCCGCATGTTGCACCGACCCA
TCGCTGCATTGCTCCAGACCTGATCGGTATGGGCAAATCCGACAAACCAGACCTGGGTTATTTCTTCGACG
ACCACGTCCGCTTCATGGATGCCTTCATCGAAGCCCTGGGTCTGGAAGAGGTCGTCCTGGTCATTCACGAC
TGGGGCTCCGCTCTGGGTTTCCACTGGGCCAAGCGCAATCCAGAGCGCGTCAAAGGTATTGCATTTATGG
AGTTCATCCGCCCTATCCCGACCTGGGACGAATGGCCAGAATTTGCCCGCGAGACCTTCCAGGCCTTCCG
CACCACCGACGTCGGCCGCAAGCTGATCATCGATCAGAACGTTTTTATCGAGGGTACGCTGCCGATGGGT
GTCGTCCGCCCGCTGACTGAAGTCGAGATGGACCATTACCGCGAGCCGTTCCTGAATCCTGTTGACCGCG
AGCCACTGTGGCGCTTCCCAAACGAGCTGCCAATCGCCGGTGAGCCAGCGAACATCGTCGCGCTGGTCG
AAGAATACATGGACTGGCTGCACCAGTCCCCTGTCCCGAAGCTGCTGTTCTGGGGCACCCCAGGCGTTCT
GATCCCACCGGCCGAAGCCGCTCGCCTGGCCAAAAGCCTGCCTAACTGCAAGGCTGTGGACATCGGCCC
GGGTCTGAATCTGCTGCAAGAAGACAACCCGGACCTGATCGGCAGCGAGATCGCGCGCTGGCTGTCAAC
GCTCGAGATTTCCGGCGACTACAAAGACCATGACGGTGATTATAAAGATCATGACATCGATTACAAGGAT
GACGATGACAAGTGAGTGCCCACGGAGGCCCCCAGCACACCCAACGTGGCTTGATCACCTGCCTGTCCA
GCTCTGGTGGGCCAAGAACCCACCCAACAGAATAGGCCAGCCCATGCCAGCCGGCTTGGCCCGCTGCA
GGCCTCAGGCAGGCGGGGCCCATGGTTGGTCCCTGCGGTGGGACCGGATCTGGGCCTGCCTCTGAGAA
GCCCTGAGCTACCTTGGGGTCTGGGGTGGGTTTCTGGGAAAGTGCTTCCCCAGAACTTCCCTGGCTCCTG
GCCTGTGAGTGGTGCCACAGGGGCACCCCAGCTGAGCCCCTCACCGGGAAGGAGGAGACCCCCGTGGG

CACGTGTCCACTTTTAATCAGGGGACAGGGCTCTCTAATAAAGCTGCTGGCAGTGCCCAGGTACCAACTT
GTTTATTGCAGCTTATAATGGTTACAAATAAAGCAATAGCATCACAAATTTCACAAATAAAGCATTTTTTTC
ACTGCATTCTAGTTGTGGTTTGTCCAAACTCATCAATGTATCTTAAACTTGTTTATTGCAGCTTATAATGGTTA
CAAATAAAGCAATAGCATCACAAATTTCACAAATAAAGCATTTTTTTCACTGCATTCTAGTTGTGGTTTGTC
CAAACTCATCAATGTATCTTAAACTTGTTTATTGCAGCTTATAATGGTTACAAATAAAGCAATAGCATCACA
AATTTCACAAATAAAGCATTTTTTTCACTGCATTCTAGTTGTGGTTTGTCCAAACTCATCAATGTATCTTAGA
CTGTGCCTTCTAGTTGCCAGCCATCTGTTGTTTGCCCCTCCCCCGTGCCTTCCTTGACCCTGGAAGGTGCCA
CTCCCACTGTCCTTTCCTAATAAAATGAGGAAATTGCATCGCATTGTCTGAGTAGGTGTCATTCTATTCTGG
GGGGTGGGGTGGGGCAGGACAGCAAGGGGGAGGATTGGGAAGAGAATAGCAGGCATGCGGCCGCGA
TATCATAACTTCGTATAATGTATGCTATACGAAGTTATGGGGTTGGGGTTGCGCCTTTTCCAAGGCAGCCCT
GGGTTTGCGCAGGGACGCGGCTGCTCTGGGCGTGGTTCCGGGAAACGCAGCGGCGCCGACCCTGGGTC
TCGCACATTCTTCACGTCCGTTCGCAGCGTCACCCGGATCTTCGCCGCTACCCTTGTGGGCCCCCCGGCGA
CGCTTCCTGCTCCGCCCCTAAGTCGGGAAGGTTCCTTGCGGTTCGCGGCGTGCCGGACGTGACAAACGG
AAGCCGCACGTCTCACTAGTACCCTCGCAGACGGACAGCGCCAGGGAGCAATGGCAGCGCGCCGACCG
CGATGGGCTGTGGCCAATAGCGGCTGCTCAGCAGGGCGCGCCGAGAGCAGCGGCCGGGAAGGGGCGG
TGCGGGAGGCGGGGTGTGGGGCGGTAGTGTGGGCCCTGTTCCTGCCCGCGCGGTGTTCCGCATTCTGCA
AGCCTCCGGAGCGCACGTCGGCAGTCGGCTCCCTCGTTGACCGAATCACCGACCTCTCTCCCCAGCAATT
CACCATGACCGAGTACAAGCCCACGGTGCGCCTCGCCACCCGCGACGACGTCCCCAGGGCCGTACGCAC
CCTCGCCGCCGCGTTCGCCGACTACCCCGCCACGCGCCACACCGTCGATCCGGACCGCCACATCGAGCG
GGTCACCGAGCTGCAAGAACTCTTCCTCACGCGCGTCGGGCTCGACATCGGCAAGGTGTGGGTCGCGGA
CGACGGCGCCGCGGTGGCGGTCTGGACCACGCCGGAGAGCGTCGAAGCGGGGGCGGTGTTCGCCGAG
ATCGGCCCGCGCATGGCCGAGTTGAGCGGTTCCCGGCTGGCCGCGCAGCAACAGATGGAAGGCCTCCT
GGCGCCGCACCGGCCCAAGGAGCCCGCGTGGTTCCTGGCCACCGTCGGCGTCTCGCCCGACCACCAGG
GCAAGGGTCTGGGCAGCGCCGTCGTGCTCCCCGGAGTGGAGGCGGCCGAGCGCGCCGGGGTGCCCGC
CTTCCTGGAGACCTCCGCGCCCCGCAACCTCCCCTTCTACGAGCGGCTCGGCTTCACCGTCACCGCCGAC
GTCGAGGTGCCCGAAGGACCGCGCACCTGGTGCATGACCCGCAAGCCCGGTGCCTGACTCGAGTCTAGA
CCAATTGGTTTAAACCCTGCAGGCTGTGCCTTCTAGTTGCCAGCCATCTGTTGTTTGCCCCTCCCCCGTGCC
TTCCTTGACCCTGGAAGGTGCCACTCCCACTGTCCTTTCCTAATAAAATGAGGAAATTGCATCGCATTGTCT
GAGTAGGTGTCATTCTATTCTGGGGGGTGGGGTGGGGCAGGACAGCAAGGGGGAGGATTGGGAAGACA

ATAGCAGGCATGCTGGGGATGCGGTGGGCTCTATGGATAACTTCGTATAATGTATGCTATACGAAGTTATCC

TAGGTACAGGGAAGACGCTGTGCCTGCTGTGCACCACGCTGGCCTGGCGAGAACACCTCCGAGACGGC

ATCTCTGCCCGCAAGATTGCCGAGAGGGCGCAAGGAGAGCTTTTCCCGGATCGGGCCTTGTCATCCTGG

GGCAACGCTGCTGCTGCTGCTGGAGACCCCATAGGTGACCCTAGTTCCCAGGCCTCTCCTGGCCTCCTGT

GGGGATGGTTGGCAAGGGATGGCGCTGAGGGTGGGGTGGGCCCATGGGGACTCCTGCCGTCTCTCAAG

CAGAACTCAAGGAGAATTTTTTAGCTGCTGTATAATTTCTCGCCATCGTGGGTGTAAACCTAGGGTTGGGC

TTTTTTGCTGAATTAGGGCACGGCAGATGCCCACTTCACCCATTTTTGATAAACCAGTATCTGGGGTGTCA

GATTCTTGGCTGTCTGCAGGGCCGAGTTAGCCGAATGCCACCTGCCTTTGATACGTGAGAACGTTGTCTGA

GAACCGTGACTTCTGTGCTTGCTTGTGTCTGGTCAGCTTGCTACACGGACATCCCAAAGATTATTTACGCCT

CCAGGACCCACTCGCAACTCACACAGGTCATCAACGAGCTTCGGAACACCTCCTACCGGTGGGTCAGAC

GAGTTTACACCTGTCTCGGGGTCCTCAAGAGAACCAGCTTGGCATGGTGCTGAGTCCACAGCCCCATGCT

GTGCTGTGGTGGAGGGTGGTGGTCTTTCTAGACGCTCCCCCGAAGTGTGCAGAGCGCTGGTGCCCAGGG

GTGGGGTGCGGCCTGGGCTGCCTCCAATGCCCATTACTTGTGAGGAAGCAGCTTTGCATCTGTGTGCTGA

CCTTGGGCGGGCGTCCTGAGCTCCTCGCAGGTGCTGTTGTAGCAGCTGTGCAGTAGGTCAGGGCTGGCC

CCCAGTGCAGCTTTGCACATGAAGTAGGAGGAGGCCCTGCTGCTTGTCAGAGCCCAGCAGAGTCTTGGT

GTTCTGTCGGGTTCCTGGTCGACCTGCAGGCATGCAAGCTTGGCGTAATCATGGTCATAGCTGTTTCCTGT

GTGAAATTGTTATCCGCTCACAATTCCACACAACATACGAGCCGGAAGCATAAAGTGTAAAGCCTGGGGT

GCCTAATGAGTGAGCTAACTCACATTAATTGCGTTGCGCTCACTGCCCGCTTTCCAGTCGGGAAACCTGTC

GTGCCAGCTGCATTAATGAATCGGCCAACGCGCGGGGAGAGGCGGTTTGCGTATTGGGCGCTCTTCCGC

TTCCTCGCTCACTGACTCGCTGCGCTCGGTCGTTCGGCTGCGGCGAGCGGTATCAGCTCACTCAAAGGCG

GTAATACGGTTATCCACAGAATCAGGGGATAACGCAGGAAAGAACATGTGAGCAAAAGGCCAGCAAAA

GGCCAGGAACCGTAAAAAGGCCGCGTTGCTGGCGTTTTTCCATAGGCTCCGCCCCCCTGACGAGCATCA

CAAAAATCGACGCTCAAGTCAGAGGTGGCGAAACCCGACAGGACTATAAAGATACCAGGCGTTTCCCCC

TGGAAGCTCCCTCGTGCGCTCTCCTGTTCCGACCCTGCCGCTTACCGGATACCTGTCCGCCTTTCTCCCTTC

GGGAAGCGTGGCGCTTTCTCATAGCTCACGCTGTAGGTATCTCAGTTCGGTGTAGGTCGTTCGCTCCAAGC

TGGGCTGTGTGCACGAACCCCCCGTTCAGCCCGACCGCTGCGCCTTATCCGGTAACTATCGTCTTGAGTCC

AACCCGGTAAGACACGACTTATCGCCACTGGCAGCAGCCACTGGTAACAGGATTAGCAGAGCGAGGTAT

GTAGGCGGTGCTACAGAGTTCTTGAAGTGGTGGCCTAACTACGGCTACACTAGAAGGACAGTATTTGGTA

TCTGCGCTCTGCTGAAGCCAGTTACCTTCGGAAAAAGAGTTGGTAGCTCTTGATCCGGCAAACAAACCAC

CGCTGGTAGCGGTGGTTTTTTTGTTTGCAAGCAGCAGATTACGCGCAGAAAAAAAGGATCTCAAGAAGA
TCCTTTGATCTTTTCTACGGGGTCTGACGCTCAGTGGAACGAAAACTCACGTTAAGGGATTTTGGTCATGA
GATTATCAAAAAGGATCTTCACCTAGATCCTTTTAAATTAAAAATGAAGTTTTAAATCAATCTAAAGTATATA
TGAGTAAACTTGGTCTGACAGTTACCAATGCTTAATCAGTGAGGCACCTATCTCAGCGATCTGTCTATTTCG
TTCATCCATAGTTGCCTGACTCCCCGTCGTGTAGATAACTACGATACGGGAGGGCTTACCATCTGGCCCCA
GTGCTGCAATGATACCGCGAGACCCACGCTCACCGGCTCCAGATTTATCAGCAATAAACCAGCCAGCCGG
AAGGGCCGAGCGCAGAAGTGGTCCTGCAACTTTATCCGCCTCCATCCAGTCTATTAATTGTTGCCGGGAA
GCTAGAGTAAGTAGTTCGCCAGTTAATAGTTTGCGCAACGTTGTTGCCATTGCTACAGGCATCGTGGTGTC
ACGCTCGTCGTTTGGTATGGCTTCATTCAGCTCCGGTTCCCAACGATCAAGGCGAGTTACATGATCCCCCA
TGTTGTGCAAAAAAGCGGTTAGCTCCTTCGGTCCTCCGATCGTTGTCAGAAGTAAGTTGGCCGCAGTGTTA
TCACTCATGGTTATGGCAGCACTGCATAATTCTCTTACTGTCATGCCATCCGTAAGATGCTTTTCTGTGACTG
GTGAGTACTCAACCAAGTCATTCTGAGAATAGTGTATGCGGCGACCGAGTTGCTCTTGCCCGGCGTCAATA
CGGGATAATACCGCGCCACATAGCAGAACTTTAAAAGTGCTCATCATTGGAAAACGTTCTTCGGGGCGAA
AACTCTCAAGGATCTTACCGCTGTTGAGATCCAGTTCGATGTAACCCACTCGTGCACCCAACTGATCTTCA
GCATCTTTTACTTTCACCAGCGTTTCTGGGTGAGCAAAAACAGGAAGGCAAAATGCCGCAAAAAAGGGA
ATAAGGGCGACACGGAAATGTTGAATACTCATACTCTTCCTTTTTCAATATTATTGAAGCATTTATCAGGGT
TATTGTCTCATGAGCGGATACATATTTGAATGTATTTAGAAAAATAAACAAATAGGGGTTCCGCGCACATTT
CCCCGAAAAGTGCCACCTGACGTCTAAGAAACCATTATTATCATGACATTAACCTATAAAAATAGGCGTATC
ACGAGGCCCTTTCGTC

> HA-mEos3.2-FKBP12^{F36V}-TRF2_DonorVector

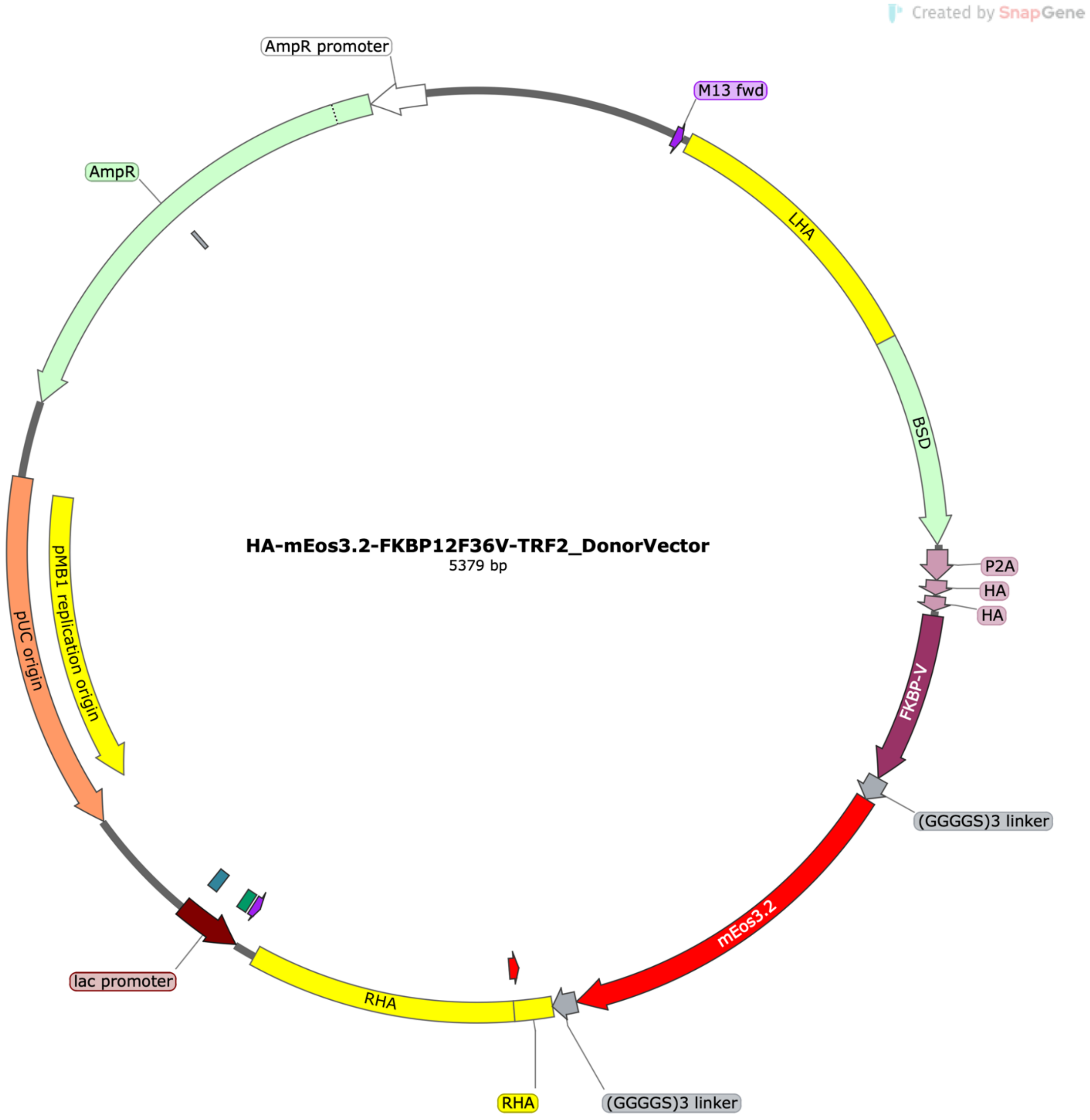

TCGCGCGTTTCGGTGATGACGGTGAAAACCTCTGACACATGCAGCTCCCGGAGACGGTCACAGCTTGTCT

GTAAGCGGATGCCGGGAGCAGACAAGCCCGTCAGGGCGCGTCAGCGGGTGTTGGCGGGTGTCGGGGCT

GGCTTAACTATGCGGCATCAGAGCAGATTGTACTGAGAGTGCACCATATGCGGTGTGAAATACCGCACAG

ATGCGTAAGGAGAAAATACCGCATCAGGCGCCATTCGCCATTCAGGCTGCGCAACTGTTGGGAAGGGCG

ATCGGTGCGGGCCTCTTCGCTATTACGCCAGCTGGCGAAAGGGGGATGTGCTGCAAGGCGATTAAGTTG

GGTAACGCCAGGGTTTTCCCAGTCACGACGTTGTAAAACGACGGCCAGTGAATTCGAGCTCTTTTTTGAG

ACGGAGTTTCGCTCTTGTTGCCCAGACTGGAGTGCAATGGCGCGATCTCGGCTCACCGCAACCTCCGCCT
CGCGGGTTCAAGCGAGTCTCCTGCCTCAGCCTCCTGAGTAGCTAGGATTACAGGCATGCGCCACCACGTC
TGGCTAATTTTGTATTTTTAGTAGAGATGGGGTTTCTCCATGTTGGTCAGGCTGGTCTCGAACTCCCGACCT
CAGGTGATCCACCCGCCTCAGCCTCCCAAAGTGCTGGGATTACAGGCGTAGAGCCACCGATCCCGGCCTG
TTTTTCAGTCTCTAATACACTGTTCCGCGTCCCTAGCATAGTAGCTGTTTTCTGTAAATTCGGAGCGAAGTT
GACTGCAAAACCCGCCGGGTTCACCGGCCTGGGCGGTGTGGACTTCAAGCCCCAGGAGGCATTGCGGC
CGGCACATCGGGAACTACGGCGTCTGAGAAGGGGGCCGGGCCCTCCGGTCCTTTCCCTCCCAGAAGCCC
GCGGGCGGCTCGGAACGCTGTTTCTATCATGGCCAAGCCTTTGTCTCAAGAAGAATCCACCCTCATTGAA
AGAGCAACGGCTACAATCAACAGCATCCCCATCTCTGAAGACTACAGCGTCGCCAGCGCAGCTCTCTCTA
GCGACGGCCGCATCTTCACTGGTGTCAATGTATATCATTTTACTGGGGGACCTTGTGCAGAACTCGTGGTG
CTGGGCACTGCTGCTGCTGCGGCAGCTGGCAACCTGACTTGTATCGTCGCGATCGGAAATGAGAACAGG
GGCATCTTGAGCCCCTGCGGACGGTGCCGACAGGTGCTTCTCGATCTGCATCCTGGGATCAAAGCCATAG
TGAAGGACAGTGATGGACAGCCGACGGCAGTTGGGATTCGTGAATTGCTGCCCTCTGGTTATGTGTGGGA
GGGCGGAAGCGGAGCTACTAACTTCAGCCTGCTGAAGCAGGCTGGAGACGTGGAGGAGAACCCTGGA
CCTTACCCCTACGACGTGCCCGACTACGCCGGCTATCCGTATGATGTCCCGGACTATGCAGGAAGCGGAG
GAGTGCAGGTGGAAACCATCTCCCCAGGAGACGGGCGCACCTTCCCCAAGCGCGGCCAGACCTGCGTG
GTGCACTACACCGGGATGCTTGAAGATGGAAAGAAAGTTGATTCCTCCCGGGACAGAAACAAGCCCTTT
AAGTTTATGCTAGGCAAGCAGGAGGTGATCCGAGGCTGGGAAGAAGGGGTTGCCCAGATGAGTGTGGG
TCAGAGAGCCAAACTGACTATATCTCCAGATTATGCCTATGGTGCCACTGGGCACCCAGGCATCATCCCAC
CACATGCCACTCTCGTCTTCGATGTGGAGCTTCTAAAACTGGAAGGAGGCGGTGGATCGGGAGGCGGTG
GATCGGGAGGCGGTGGATCGAGTGCGATTAAGCCAGACATGAAGATCAAACTCCGTATGGAAGGCAACG
TAAACGGGCACCACTTTGTGATCGACGGAGATGGTACAGGCAAGCCTTTTGAGGGAAAACAGAGTATGG
ATCTTGAAGTCAAAGAGGGCGGACCTCTGCCTTTTGCCTTTGATATCCTGACCACTGCATTCCATTACGGC
AACAGGGTATTCGCCAAATATCCAGACAACATACAAGACTATTTTAAGCAGTCGTTTCCTAAGGGGTATTC
GTGGGAACGAAGCTTGACTTTCGAAGACGGGGGCATTTGCAACGCCAGAAACGACATAACAATGGAAG
GGGACACTTTCTATAATAAAGTTCGATTTTATGGTACCAACTTTCCCGCCAATGGTCCAGTTATGCAGAAGA
AGACGCTGAAATGGGAGCCCTCCACTGAGAAAATGTATGTGCGTGATGGAGTGCTGACGGGTGATATTGA
GATGGCTTTGTTGCTTGAAGGAAATGCCCATTACCGATGTGACTTCAGAACTACTTACAAAGCTAAGGAG
AAGGGTGTCAAGTTACCAGGCGCCCACTTTGTGGACCACTGCATTGAGATTTTAAGCCATGACAAAGATT

ACAACAAGGTTAAGCTGTATGAGCATGCTGTTGCTCATTCTGGATTGCCTGACAATGCCAGACGAGGAGG
CGGTGGATCGGGAGGCGGTGGATCGGGAGGCGGTGGATCGATGGCCGCGGGAGCCGGGACGGCGGGC
CCCGCTTCCGGCCCGGGCGTCGTGCGTGACCCAGCAGCGTCACAGCCGAGAAAGCGGCCCGGCCGGGA
GGGCGGGGAGGGCGCGCGGCGATCGGACACGATGGCGGGAGGAGGCGGGAGTAGCGACGGCAGCG
GGCGGGCAGCTGGCAGGCGGGCGTCCCGCAGTAGCGGGCGGGCCCGGCGGGGGCGCCACGAGCCGG
GGCTGGGGGGCCCGGCGGAGCGCGGCGCGGGGGAGGCACGGCTGGAAGAGGCAGTCAATCGCTGGG
TGCTCAAGTTCTACTTCCACGAGGCGCTGCGGGCCTTTCGGGGTAGCCGGTACGGGGACTTCAGACAGA
TCCGGGACATCATGCAGGGTGAGGGCCGGGCCGGGGGAGGGGGGTTGGAGCGCCGGGGAAGGGGGT
CCGGGGGACCGGGAGTCGGGACTGGGGGTCGCCCAGCCAACGATGTCTTCTCTCCCAGCTTTGCTTGTC
AGGCCCTTGGGGAAGGAGCACACCGTGTCCCGATTGCTGCGGGTTATGCAGTGTCTGTCGCGGATTGAA
GAAGGGGGTCGACCTGCAGGCATGCAAGCTTGGCGTAATCATGGTCATAGCTGTTTCCTGTGTGAAATTG
TTATCCGCTCACAATTCCACACAACATACGAGCCGGAAGCATAAAGTGTAAAGCCTGGGGTGCCTAATGA
GTGAGCTAACTCACATTAATTGCGTTGCGCTCACTGCCCGCTTTCCAGTCGGGAAACCTGTCGTGCCAGCT
GCATTAATGAATCGGCCAACGCGCGGGGAGAGGCGGTTTGCGTATTGGGCGCTCTTCCGCTTCCTCGCTC
ACTGACTCGCTGCGCTCGGTCGTTCGGCTGCGGCGAGCGGTATCAGCTCACTCAAAGGCGGTAATACGGT
TATCCACAGAATCAGGGGATAACGCAGGAAAGAACATGTGAGCAAAAGGCCAGCAAAAGGCCAGGAA
CCGTAAAAAGGCCGCGTTGCTGGCGTTTTTCCATAGGCTCCGCCCCCCTGACGAGCATCACAAAAATCGA
CGCTCAAGTCAGAGGTGGCGAAACCCGACAGGACTATAAAGATACCAGGCGTTTCCCCCTGGAAGCTCC
CTCGTGCGCTCTCCTGTTCCGACCCTGCCGCTTACCGGATACCTGTCCGCCTTTCTCCCTTCGGGAAGCGT
GGCGCTTTCTCATAGCTCACGCTGTAGGTATCTCAGTTCGGTGTAGGTCGTTCGCTCCAAGCTGGGCTGTG
TGCACGAACCCCCCGTTCAGCCCGACCGCTGCGCCTTATCCGGTAACTATCGTCTTGAGTCCAACCCGGTA
AGACACGACTTATCGCCACTGGCAGCAGCCACTGGTAACAGGATTAGCAGAGCGAGGTATGTAGGCGGT
GCTACAGAGTTCTTGAAGTGGTGGCCTAACTACGGCTACACTAGAAGGACAGTATTTGGTATCTGCGCTCT
GCTGAAGCCAGTTACCTTCGGAAAAAGAGTTGGTAGCTCTTGATCCGGCAAACAAACCACCGCTGGTAG
CGGTGGTTTTTTTGTTTGCAAGCAGCAGATTACGCGCAGAAAAAAAGGATCTCAAGAAGATCCTTTGATC
TTTTCTACGGGGTCTGACGCTCAGTGGAACGAAAACTCACGTTAAGGGATTTTGGTCATGAGATTATCAAA
AAGGATCTTCACCTAGATCCTTTTAAATTAAAAATGAAGTTTTAAATCAATCTAAAGTATATATGAGTAAACT
TGGTCTGACAGTTACCAATGCTTAATCAGTGAGGCACCTATCTCAGCGATCTGTCTATTTCGTTCATCCATAG
TTGCCTGACTCCCCGTCGTGTAGATAACTACGATACGGGAGGGCTTACCATCTGGCCCCAGTGCTGCAATG

ATACCGCGAGACCCACGCTCACCGGCTCCAGATTTATCAGCAATAAACCAGCCAGCCGGAAGGGCCGAG
CGCAGAAGTGGTCCTGCAACTTTATCCGCCTCCATCCAGTCTATTAATTGTTGCCGGGAAGCTAGAGTAAG
TAGTTCGCCAGTTAATAGTTTGCGCAACGTTGTTGCCATTGCTACAGGCATCGTGGTGTCACGCTCGTCGTT
TGGTATGGCTTCATTCAGCTCCGGTTCCCAACGATCAAGGCGAGTTACATGATCCCCCATGTTGTGCAAAA
AAGCGGTTAGCTCCTTCGGTCCTCCGATCGTTGTCAGAAGTAAGTTGGCCGCAGTGTTATCACTCATGGTT
ATGGCAGCACTGCATAATTCTCTTACTGTCATGCCATCCGTAAGATGCTTTTCTGTGACTGGTGAGTACTCA
ACCAAGTCATTCTGAGAATAGTGTATGCGGCGACCGAGTTGCTCTTGCCCGGCGTCAATACGGGATAATAC
CGCGCCACATAGCAGAACTTTAAAAGTGCTCATCATTGGAAAACGTTCTTCGGGGCGAAAACTCTCAAG
GATCTTACCGCTGTTGAGATCCAGTTCGATGTAACCCACTCGTGCACCCAACTGATCTTCAGCATCTTTTAC
TTTCACCAGCGTTTCTGGGTGAGCAAAAACAGGAAGGCAAAATGCCGCAAAAAAGGGAATAAGGGCGA
CACGGAAATGTTGAATACTCATACTCTTCCTTTTTCAATATTATTGAAGCATTTATCAGGGTTATTGTCTCATG
AGCGGATACATATTTGAATGTATTTAGAAAAATAAACAAATAGGGGTTCCGCGCACATTTCCCCGAAAAGT
GCCACCTGACGTCTAAGAAACCATTATTATCATGACATTAACCTATAAAAATAGGCGTATCACGAGGCCCTT
TCGTC

References

1. Xi, L. and Cech, T.R. (2014) Inventory of telomerase components in human cells reveals multiple subpopulations of hTR and hTERT. *Nucleic Acids Res.*, **42**, 8565-8577.
2. Nagaraj, N., Wisniewski, J.R., Geiger, T., Cox, J., Kircher, M., Kelso, J., Pääbo, S. and Mann, M. (2011) Deep proteome and transcriptome mapping of a human cancer cell line. *Mol. Syst. Biol.*, **7**, 548.
3. Hein, M.Y., Hubner, N.C., Poser, I., Cox, J., Nagaraj, N., Toyoda, Y., Gak, I.A., Weisswange, I., Mansfeld, J., Buchholz, F. *et al.* (2015) A human interactome in three quantitative dimensions organized by stoichiometries and abundances. *Cell*, **163**, 712-723.
